# Hirshfeld Atom Refinement of Metal–Organic Complexes: Treatment of Hydrogen Atoms Bonded to Transition Metals

**DOI:** 10.1021/acs.jpca.2c06998

**Published:** 2023-03-22

**Authors:** Magdalena Woińska, Sylwia Pawlędzio, Michał L. Chodkiewicz, Krzysztof Woźniak

**Affiliations:** Biological and Chemical Research Centre, Chemistry Department, University of Warsaw, Żwirki i Wigury 101, 02-089 Warszawa, Poland

## Abstract

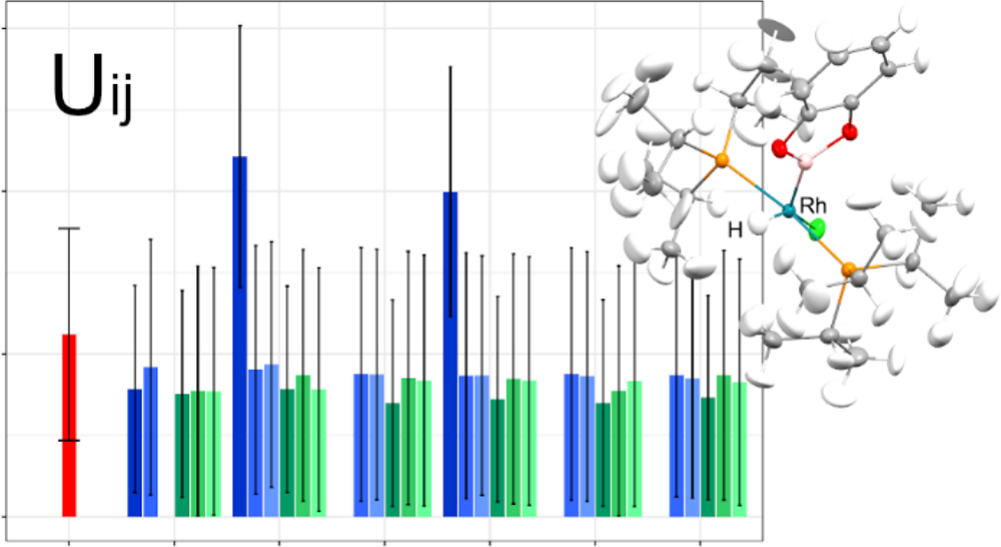

Hydrogen positions in hydrides play a key role in hydrogen storage materials and high-temperature superconductors. Our recently published study of five crystal structures of transition-metal-bound hydride complexes showed that using aspherical atomic scattering factors for Hirshfeld atom refinement (HAR) resulted in a systematic elongation of metal–hydrogen bonds compared to using spherical scattering factors with the Independent Atom Model (IAM). Even though only standard-resolution X-ray data was used, for the highest-quality data, we obtained excellent agreement between the X-ray and the neutron-derived bond lengths. We present an extended version of this study including 10 crystal structures of metal–organic complexes containing hydrogen atoms bonded to transition-metal atoms for which both X-ray and neutron data are available. The neutron structures were used as a benchmark, and the X-ray structures were refined by applying Hirshfeld atom refinement using various basis sets and DFT functionals in order to investigate the influence of the technical aspects on the length of metal–hydrogen bonds. The result of including relativistic effects in the Hamiltonian and using a cluster of multipoles simulating interactions with a crystal environment during wave function calculations was examined. The effect of the data quality on the final result was also evaluated. The study confirms that a high quality of experimental data is the key factor allowing us to obtain significant improvement in transition metal (TM)–hydrogen bond lengths from HAR in comparison with the IAM. Individual adjustments and better choices of the basis set can improve hydrogen positions. Average differences between TM–H bond lengths obtained with various DFT functionals upon including relativistic effects or between double-ζ and triple-ζ basis sets were not statistically significant. However, if all bonds formed by H atoms were considered, significant differences caused by different refinement strategies were observed. Finally, we examined the refinement of atomic thermal motions. Anisotropic refinement of hydrogen thermal motions with HAR was feasible only in some cases, and isotropically refined hydrogen thermal motions were in similar agreement with neutron values whether obtained with HAR or with the IAM.

## Introduction

Hydrogen positions are absolutely essential in the most important biological substances: H_2_O, proteins, DNA, and polysaccharides, DNA base pairing, and other phenomena crucial to life. The hydrogen position is also absolutely crucial in the case of hydrogen bonding, which is one of the most important interactions in modern biology, medicine and pharmacology, and materials science. Hydrogen positions in hydrides determine the properties of important hydrogen storage materials and high-temperature superconductors. The Independent Atom Model (IAM),^1^ the most commonly applied model of electron density in the refinement of X-ray diffraction data, is a simplified approach that treats atomic electron densities as spherical densities of isolated atoms. It does not provide a correct description of aspherical electron density deformations arising from the presence of lone electron pairs, bond formation, and other interactions between molecules in crystals. The lack of asphericity in the IAM substantially affects the description of the electron density of hydrogen atoms, which possess only one valence electron and the density of which is usually strongly shifted toward the atom they are bonded to. The most important consequence of this is underestimation of the bond lengths formed by hydrogen atoms (on average by 0.12 Å in X–H bond types, which are common in crystals of organic compounds^2^). Furthermore, the IAM does not allow for refinement of anisotropic displacement parameters (ADPs) for hydrogen atoms. These two problematic aspects of the IAM are alleviated by Hirshfeld atom refinement (HAR),^3,4^ which implements a more advanced electron density model, namely, the Hirshfeld model.^5^ For hydrogen atoms bonded to lighter chemical elements, forming X–H bonds typical for crystals of organic compounds, the mean X–H bond lengths obtained with HAR are underestimated by 0.01–0.02 Å compared to the neutron values^2^ (on average by only 0.014 Å^6^). Such an improvement can be obtained with standard-resolution, good-quality X-ray data. Anisotropic treatment of hydrogen atom thermal motions is generally possible with HAR if one allows for an expected lower accuracy compared to ADPs derived from neutron diffraction or estimated with other methods^2,7^ as well as the fact that, if the data quality is not sufficient, only isotropic refinement can be successful.^7,8^

Transition metal (TM)-bound hydrides are of particular interest in many fields of chemistry. Many metals and alloys composed of transition metals can reversibly absorb large amounts of hydrogen and thus can be used as hydrogen storage materials.^9−11^ Many transition-metal hydrides are also known as superconductors and high-temperature superconductors^12,13^ with the best-known examples of Pd and Pd-Ni hydrides^14^ and also V,^15^ Cr,^16^ Nb, Ta,^17^ and Th.^18^ Transition-metal hydrides are catalysts or intermediate compounds in numerous chemical processes, such as energy conversion,^19−21^ catalytic hydrogenation, and reactions involving C–H bond activation.^22−25^ X-ray crystallography is a very helpful technique supporting the research of TM hydrides; however, the already described problematic aspect of establishing positions of hydrogen atoms is even more exacerbated in this group of compounds. One reason why the position of the hydrogen atom in the vicinity of a transition-metal atom is particularly difficult to determine is strong screening from the electron-rich metal atom. Another problem is that truncation of the Fourier summation based on X-ray diffraction data due to the limited resolution may manifest itself in the resulting electron density in the region of the hydrogen atom neighboring the TM, and therefore collecting high-resolution data would be recommended.^26^ On the other hand, obtaining high-quality, let alone high-resolution, data for this group of compounds is difficult since their crystals are vulnerable to problems such as absorption and radiation-damage effects. Lastly, modeling the electron density of transition metals is a challenging task as it may require very accurate and time-consuming quantum mechanical calculations, including relativistic effects if HAR is to be performed. On top of that, the validation of hydrogen positions determined with the given X-ray data analysis method requires a suitable benchmark, that is, a neutron structure obtained for the same system, which is not easily attainable for transition-metal hydrides.

Due to the enumerated obstacles, up until our study, which was presented in a short communication,^27^ only six structures of complexes with TM–H bonds have been successfully refined with HAR;^2,26,28^ complementary neutron data is available for only two of these. Our preliminary study extended the list of successful HARs by four structures with both X-ray and neutron diffraction data available. In order to make HAR of TM hydrides attainable for a larger number of structures, suitable software had to be developed or created so that HAR could become more flexible in the choice of the computational method in order to decrease the computational cost and account for poor data quality. The first step was integration of the basic capabilities of HAR as implemented in the TONTO program^29^ with Olex2,^30^ a popular crystal structure refinement program. As a result, a version called HARt^31^ was made available in Olex2, providing a limited choice of basis sets and restricted Hartree-Fock or DFT calculations. Subsequently, TONTO was combined with an external quantum chemistry program, which allowed for a broader range of basis sets for wave function calculations and added the option of using MP2 and CCSD.^32^ Then, two TONTO-independent approaches making HAR available from Olex2 were developed: NoSPherA2 (Non-spherical Atom-Form-Factors in Olex2)^26^ and DiSCaMB (Densities in Structural Chemistry and Molecular Biology).^33^ Both of these allow for a variety of options provided by Olex2 (of which, particularly useful are restraints and constraints, weighting scheme selection, and easy visualization of the results) and the use of an external quantum mechanical program (ORCA^34,35^ or Gaussian16^36^) for performing wave function calculations. NoSpherA2 on its own does not enable surrounding the central molecule with a cluster of multipoles to model interactions with other molecules in the crystal (although this option is still available when TONTO is called via the NoSpherA2 interface); instead, it makes various solvent models available for this purpose. DiSCaMB, in turn, enables using a cluster of multipoles to model the influence of the crystal environment similar to TONTO. A more computationally expensive workaround, which is available both in NoSpherA2 and DiSCaMB, is performing quantum mechanical calculations of the wave function of the selected cluster of molecules surrounding the molecule of interest. Additionally, DiSCaMB introduces a choice of electron-density partitions and computational methods such as CCSD or MP2, increasing the number of options that can be explored to model the given system more accurately.^37^

Both NoSpherA2 and DiSCaMB enable including relativistic effects during molecular wave function calculations—a Douglas–Kroll–Hess second-order scalar relativistic Hamiltonian^38−41^ is accessible via NoSpherA2, and any relativistic approach available in Gaussian^36^ or ORCA^34,35^ can theoretically be used via DiSCaMB. Relativistic effects become particularly important for TMs starting from period VI since, for heavier elements, electrons attain higher velocities, which are a significant fraction of the velocity of light. A few X-ray structures containing TM atoms have been refined with HAR and Hamiltonian including relativistic effects,^26,28,42−45^ and only one of them was a TM-bound hydride.^26^ The latter compound was an osmium hexahydride compound for which high-resolution X-ray data was collected; however, high-resolution data did not bring any improvement compared to the data cut to the standard resolution for the Os–H bonds. To our current knowledge, the question of how important relativistic effects are for modeling correctly the positions of TM-bonded hydrogen atoms based on X-ray diffraction data has not been studied.

Our preliminary study based on five metal organic transition metal hydrides^27^ showed that non-relativistic HAR performed for such structures at the B3LYP level of theory and for standard resolution data can bring substantial improvement in terms of TM–H bond lengths provided with sufficient quality diffraction data. Herein, we present an extension of this study in which the previously mentioned five and five additional structures of TM-bound hydrides were studied both with X-ray and neutron diffraction. We explore various versions of HAR performed with different quantum mechanical functionals and basis sets (double-ζ and triple-ζ). The issue of the presence of a cluster of multipoles during wave function calculations or including relativistic effects in the Hamiltonian is also investigated.

## Experimental Data and Optimized Geometry

**Figure 1:**
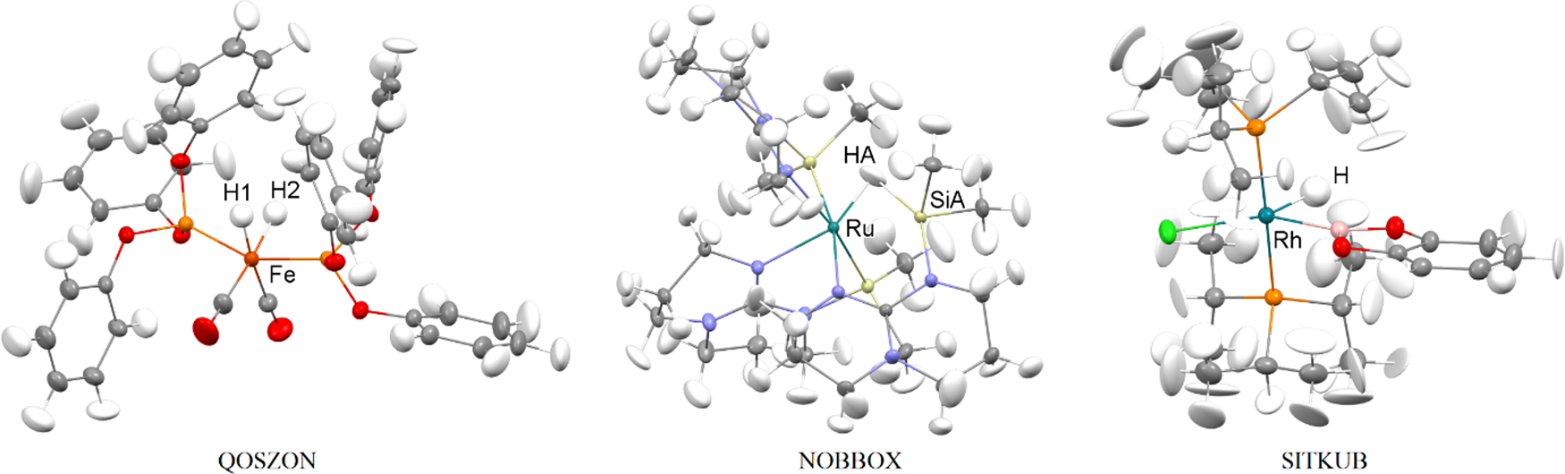
Structures obtained in the course of anisotropic DiSCaMB-HAR/B3LYP with the names of TMs, TM-bound H, and Si atoms marked. Certain H atoms were refined isotropically: QOSZON (H1 and H2) and SITKUB (H, H9A, H10, and H13). In all cases, the TM–H bond lengths were improved with DiSCaMB-HAR compared to the IAM.

**Figure 2:**
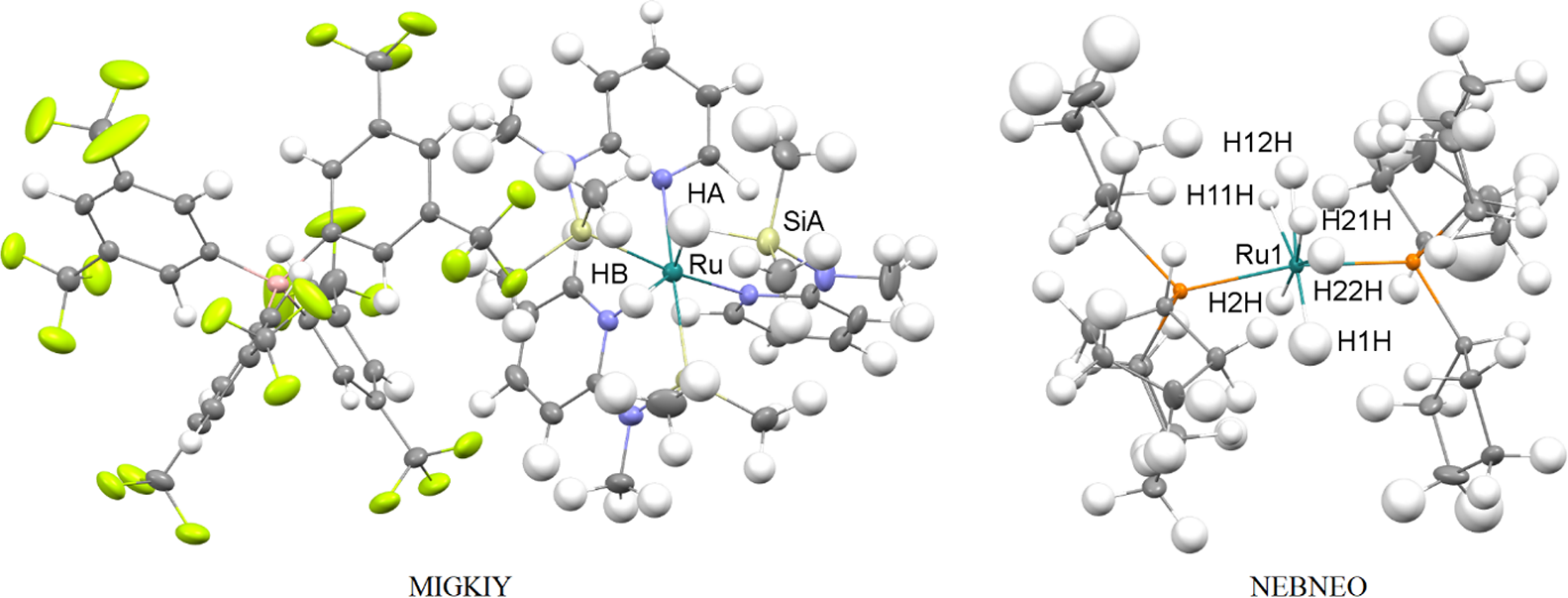
Structures obtained in the course of isotropic DiSCaMB-HAR/B3LYP with the names of TMs, TM-bound H, and Si atoms marked. In both cases, the TM–H bond lengths were improved with DiSCaMB-HAR compared to the IAM.

**Figure 3:**
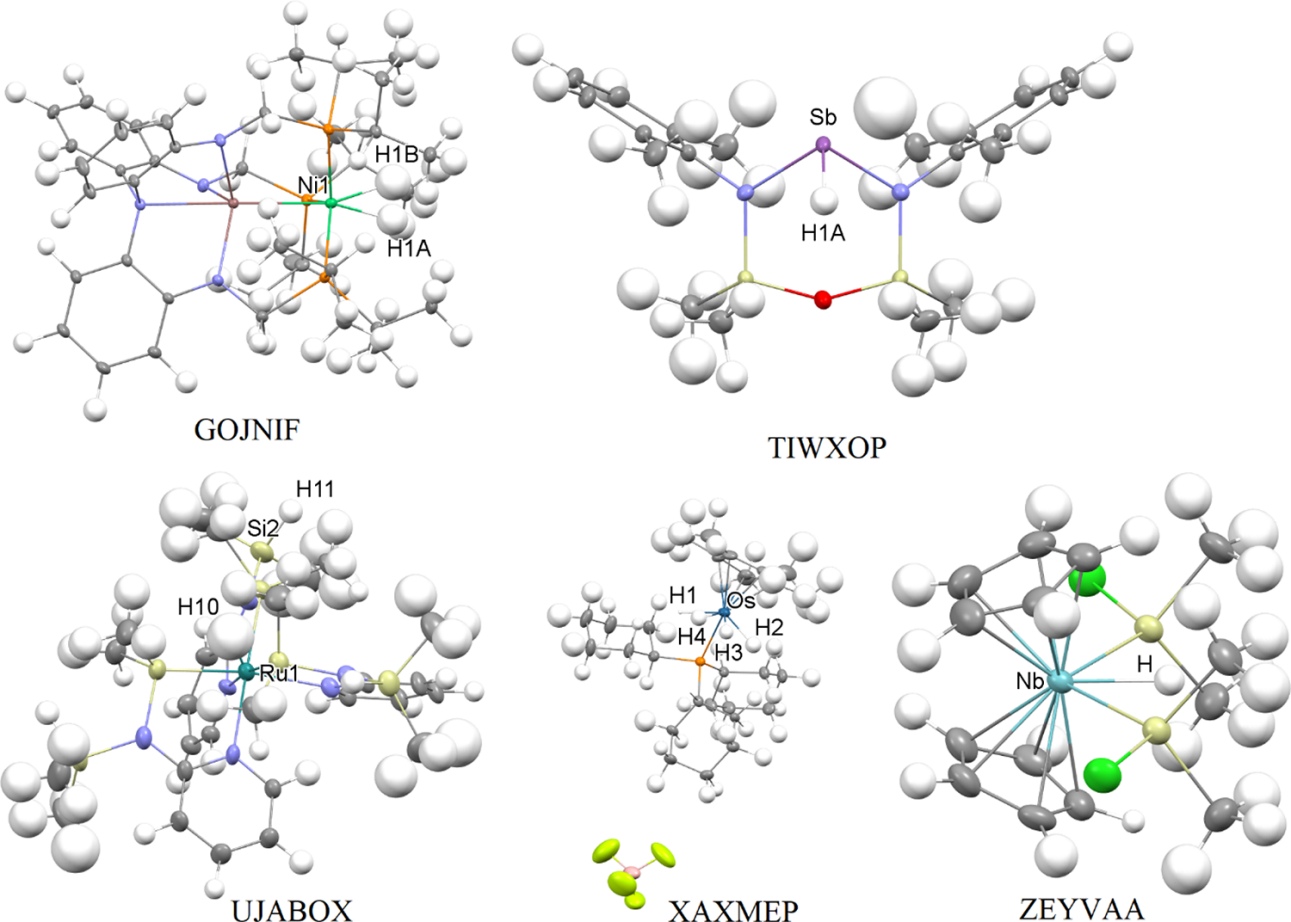
Structures obtained in the course of isotropic DiSCaMB-HAR/B3LYP with the names of TMs, TM-bound H, and Si atoms marked. In all cases, the TM–H bond lengths deteriorated with DiSCaMB-HAR compared to the IAM.

We performed a search of the Cambridge Structural Database (CSD) in order to find metal−organic complexes containing TM–hydrogen bonds for which both X-ray and neutron structures had been determined and X-ray data was available. Structures with significant disorder or other unresolved problems were excluded. As a result, 10 crystalline structures were obtained (see Figures 1, 2, and 3), which can be identified by the following REFCODEs: QOSZON,^46^^(neutron),^^47^^(X-ray)^ NEBNEO,^48^ MIGKIY,^49^ NOBBOX,^50^ SITKUB,^51^ UJABOX,^52^ ZEYVAA,^53^^(neutron),^^54^^(X-ray)^ GOJNIF,^55^ TIWXOP,^56^ and XAXMEP.^57^^(neutron),^^58^^(X-ray)^ The transition metals bonded to hydrogen atoms represent three periods of the periodic table: IV: Fe and Ni; V: Nb, Ru and Rh; and VI: Os. Among the selected structures, there is also one containing Sb, which is a metalloid from period V. Si–H bonds are also present in some of the structures. In the case of GOJNIF, the In atom, which is not bonded to any H atom, is present. This allowed us to investigate the question of how the increasing number of electrons affected data collection and what role the model of electron density used for refinement played in the final results. As an additional benchmark for hydrogen positions, geometry optimization in the gas phase was performed using Gaussian16 at the DFT/B3LYP level of theory combined with the jorge-DZP basis set.^59,60^ Geometry optimization failed for NOBBOX and MIGKIY. Geometry optimization was performed for complexes in the singlet and triplet state since, in the related literature, there was no information about the spin state of the investigated compounds. In each case in which the optimization procedure was successful, the energy of the complex in the triplet state was higher than in the singlet state; therefore, only the singlet state was taken into account during HAR.

## Refinement Types

All the deposited X-ray structures were re-refined with the IAM against the full set of reflections using olex2.refine^30^ to obtain the IAM geometries, which were substantially improved in some cases compared to the deposited structures. All the versions of HAR were derived from the deposited IAM X-ray structures. Various versions of HAR were performed in order to take into account the following factors, which have an influence on refinement results:

(a) Interactions with the crystal environment: In the first version of refinement, interactions with the crystal environment were taken into account by using a cluster of multipoles during molecular wave function calculations. The central molecule(s) was surrounded by atomic charges and dipoles centered on the nuclei of the atoms belonging to the surrounding molecules with at least one atom within 8 Å of the central molecule. Charges and dipoles were calculated in a self-consistent procedure. HAR including a cluster of charges and dipoles was performed with the DiSCaMB library.^33^ A second version of HAR, which did not include interactions with the crystal environment, was performed using NoSpherA2 software.^26^
(b) Three DFT functionals were tested: B3LYP,^61,62^ PBE,^63,64^ and M06-2X.^65,66^ B3LYP is a very popular hybrid functional with empirically determined parameters, which performs well against a variety of chemical problems, including HAR. PBE is a functional that is devoid of any empirically derived parameters and provides a higher calculation speed while preserving the resulting quality of more sophisticated functionals. The M06-2X functional is a highly parameterized functional, which is created to provide a balanced model of both elements from the main group and transition metals.
(c) Non-relativistic and relativistic versions of each HAR were performed. Relativistic effects were introduced into wave function calculations via the Douglas–Kroll–Hess second-order scalar relativistic Hamiltonian.^38−40^
(d) Various basis sets were tested. The basis sets, which could be used in HAR for given structures, were limited by the heavy element present. For some of the structures (QOSZON, GOJNIF, SITKUB, TIWXOP, and XAXMEP), performing HAR with double-ζ and triple-ζ versions of the same basis set was possible, which enables a comparison between the results obtained with double-ζ and triple-ζ basis sets. Of the investigated TMs, only Fe belongs to the group of elements with *Z* ≤ 36 for which cc-pVDZ and cc-pVTZ basis sets^67^ are available. Nb, Ru, and Rh are elements with *Z* ≤ 46 for which the cc-pVTZ-DK basis set^68^ has been defined (the double-ζ basis set cc-pVDZ-DK is not available for the fifth-row heavy elements). The remaining structures (GOJNIF, TIWXOP, and XAXMEP) contain either heavy (half)metals from the 5p group (In and Sb) or the sixth-row heavy metal Os for which, from the available basis sets, the jorge-DZP and jorge-TZP basis sets^59,60^ were selected for calculations. Moreover, since, for In and Sb, the cc-pVTZ-DK3 basis set^69,70^ is available, calculations for GOJNIF and TIWXOP were also performed with the cc-pVTZ-DK3 basis set for In or Sb and the cc-pVTZ-DK basis set for the remaining atoms. These basis sets were used in non-relativistic refinements. In the relativistic HARs, the versions of the same basis sets obtained with the Douglass–Kroll–Hess Hamiltonian were used: cc-pVDZ-DK and cc-pVTZ-DK instead of cc-pVDZ and cc-pVTZ; jorge-DZP-DKH and jorge-TZP-DKH^69^ instead of jorge-DZP and jorge-TZP. The structures and basis sets used for relativistic and non-relativistic HARs are given in Table 1.

**Table 1:** Basis Sets Used for Relativistic and Non-relativistic HARs for Each of the Structures

	basis set		
REFCODE	non-relativistic	relativistic	TM–H bonds
QOSZON	cc-pVDZ	cc-pVDZ-DK	2 Fe–H
	cc-pVTZ	cc-pVTZ-DK	
GOJNIF	jorge-DZP	jorge-DZP-DKH	2 Ni–H, In
	jorge-TZP	jorge-TZP-DKH	
	cc-pVTZ-DK^a^	cc-pVTZ-DK^a^	
ZEYVAA	cc-pVTZ-DK	cc-pVTZ-DK	1 Nb–H
NEBNEO	cc-pVTZ-DK	cc-pVTZ-DK	6 Ru–H
MIGKIY	cc-pVTZ-DK	cc-pVTZ-DK	2 Ru–H
NOBBOX	cc-pVTZ-DK	cc-pVTZ-DK	1 Ru–H
UJABOX	cc-pVTZ-DK	cc-pVTZ-DK	1 Ru–H
SITKUB	cc-pVTZ-DK	cc-pVTZ-DK	1 Rh–H
	jorge-DZP	jorge-DZP-DKH	
	jorge-TZP	jorge-TZP-DKH	
TIWXOP	jorge-DZP	jorge-DZP-DKH	1 Sb–H
	jorge-TZP	jorge-TZP-DKH	
	cc-pVTZ-DK^a^	cc-pVTZ-DK^a^	
XAXMEP	jorge-DZP	jorge-DZP-DKH	4 Os–H
	jorge-TZP	jorge-TZP-DKH	

a cc-pVTZ-DK3 for In in GOJNIF and Sb in TIWXOP.

In the case of the refinements carried out with DiSCaMB, wave function calculations were performed with Gaussian16,^36^ whereas in the case of the refinements carried out with NoSpherA2, wave function calculations were performed with ORCA.^34,35^ All the basis sets used for calculations that were not available in the software used were downloaded from the Basis Set Exchange webpage.^71^ During DiSCaMB-HAR, HORTON^72^ was used to partition the electron density into atomic contributions. In all refinement cases, the aspherical atomic scattering factors based on the Hirshfeld partition were used in the least-squares refinement performed with Olex2. NoSpherA2 uses a maximum parameter shift/sigma < 0.01 for the convergence threshold between the geometries obtained with scattering factors from subsequent cycles. In the case of DiSCaMB-HAR, calculations with a cluster of charges and dipoles are so time-consuming (we used 24 CPUs of an HPC cluster to perform quantum mechanical calculations and obtain atomic scattering factors) and may take up to a few weeks in that the most reasonable convergence criterion seems to be a maximum parameter shift/sigma < 1 as it usually allows us to achieve stable values of bond lengths. This criterion for the metal organic compounds considered in this study was met within 3–6 cycles of the DiSCaMB-HAR procedure. The NoSpherA2 and DiSCaMB version of HAR performed for the analyzed structures differed in many technical aspects, and for this reason, refinement strategies applied in both cases were also different to a certain extent. The NoSpherA2 version of HAR was performed in a more automated way, and for this reason, the SHELX weighting scheme was used in every refinement. In the case of NoSpherA2-HAR, removing g-functions from the basis sets used in quantum mechanical calculations for some of the structures made wave function calculations or refinement convergence attainable. In the case of DiSCaMB, least-squares refinement was usually started with SHELX weights and then it was switched to statistical weights unless it caused problems such as deteriorated positions of hydrogen atoms, convergence issues, or collapsing crystal structures. In some cases, starting least-squares refinement with atomic scattering factors calculated in the new cycle from the same values of SHELX weights as in the previous cycle or using only statistical weights was necessary for the refinement to be feasible.

## Crystal Environment, Relativistic Effects, Functional, and Basis Set Comparison

In total, 216 refinements were attempted and 108 (100%) NoSPherA2-HARs were successful, whereas 105 (97%) successful DiSCaMB-HARs were accomplished (the reason for failure of three refinements performed for GOJNIF was failure of wave function calculations; see Table S8). The number of results in terms of TM–H bond lengths determined allows for using statistical methods in order to verify the performance of HAR when the following parameters/conditions were varied: (a) including (or not) in-crystal interactions via a cluster of multipoles, (b) relativistic effects, (c) different DFT functionals, and (d) the difference between double-ζ and triple-ζ basis sets. Each bond length obtained with HAR was referred to the benchmark neutron bond length, which means that differences and absolute differences between the HAR and neutron TM–H bond length were calculated along with the respective compound standard deviations (, where σ_X_ is the standard deviation of the X-ray(HAR) bond length and σ_N_ is the standard deviation of the neutron bond length). Our strategy to investigate the importance of each factor (a–d) on TM–H bond lengths was the following: for example, for one, the mean differences (MDs) between bond lengths obtained from HAR with the cluster of multipoles and the neutron values are calculated and compared to the MDs between the bond lengths obtained with HAR without the cluster of multipoles and the neutron values. In this way, we compare MDs between HAR and neutron bond lengths including the effect of the crystal environment to ones that exclude this effect. In order to check whether interactions with the crystal environment have an effect on the TM–H bond lengths, we performed a statistical test of equality of MDs. We selected the two-sided Welch’s t-test^73^ to be suitable for comparing means of two populations with unequal variances. The populations of bond length differences are usually not normally distributed; however, they are sufficiently numerous for the MDs to be approximately normally distributed. For case c in which three different DFT functionals had to be compared, pairwise testing was performed. MDs calculated between HAR and neutron bond lengths divided by csd’s (denoted as MD/csd) were also averaged and tested for equality. Additionally, we also calculated the mean absolute differences (MADs) between the X-ray(HAR) and neutron bond lengths for each group of bonds, including scaling by the csd (MAD/csd). The values of MD, MD/csd, MAD, and MAD/csd between X-ray(HAR) and neutron bond lengths with the corresponding sample standard deviations and averaged csd’s for different conditions (a–d) are presented in Table 2. At first glance, the values of both MD and MD/csd (MAD and MAD/csd) seemed fairly similar between the different conditions tested in a–d especially when compared to their sample standard deviations. MDs and MD/csd’s are the measure of the tendency of the given refinement method to under/overestimate the bond lengths compared to the neutron values. MADs and MAD/csd’s in turn are the measure of the tendency of a given method to produce bond lengths deviating from the neutron values in any direction. All MDs and MD/csd’s are positive, which means that, on average, for the analyzed compounds, HAR tends to overestimate TM–H bond lengths for the conditions/parameters studied and the discrepancies from the neutron values are very similar for the refinement types considered. The results of Welch’s test presented in Table 3 confirm that the MDs and MADs can be considered equal at a significance level of 5%. Despite the fact that, on average, conditions a–d did not seem to influence the HAR TM–H bond lengths in individual cases, they caused differences in the positions of the H atoms bonded to TMs, which could be easily noticed when looking at the individual results for each structure.

**Table 2:** Values of MD, MD Calculated for Differences Divided by csd (MD/csd), MAD, and MAD Calculated for Differences Divided by csd (MAD/csd) between X-ray(HAR) and Neutron TM–H Bond Lengths with the Corresponding Sample Standard Deviations and Averaged csd’s for Different Conditions: (a) Including or Not In-Crystal Interactions via a Cluster of Multipoles (Cluster/No Cluster), (b) Relativistic Effects, (c) Different DFT Functionals, and (d) the Difference between Double-ζ and Triple-ζ basis sets

	MD [Å]	σ [Å]	MD/csd	σ	MAD [Å]	σ [Å]	MAD/csd	σ	mean csd [Å]
cluster	0.016	0.071	0.34	2.06	0.056	0.046	1.68	1.23	0.036
no cluster	0.017	0.075	0.19	1.74	0.061	0.047	1.54	0.83	0.040
non-rel	0.031	0.065	0.57	1.92	0.060	0.041	1.71	1.05	0.036
rel	0.027	0.067	0.48	2.03	0.060	0.040	1.74	1.15	0.038
B3LYP	0.014	0.077	0.23	1.99	0.060	0.050	1.65	1.13	0.038
PBE	0.015	0.075	0.22	1.94	0.059	0.049	1.62	1.08	0.038
M062X	0.020	0.067	0.35	1.79	0.056	0.041	1.55	0.94	0.038
double-ζ	0.048	0.047	0.95	1.44	0.056	0.036	1.49	0.86	0.038
triple-ζ	0.038	0.056	0.51	2.06	0.058	0.035	1.68	1.28	0.038

**Table 3:** Results of Welch’s T-Test (*p* Value) in which the Zero Hypothesis (H_0_) Was the Equality of MD, MD Calculated for Differences Divided by csd (MD/csd), MAD, and MAD Calculated for Differences Divided by csd (MAD/csd) between X-ray(HAR) and Neutron TM–H Bond Lengths with the Corresponding Sample Standard Deviations and Averaged csd’s between Different Conditions: (a) Including or Not In-Crystal Interactions via a Cluster of Multipoles (Cluster/No Cluster), (b) Relativistic Effects, (c) Different DFT Functionals, and (d) the Difference between Double-ζ and Triple-ζ basis sets

	*p* value			
	MD	MD/csd	MAD	MAD/csd
cluster–no cluster	0.85	0.43	0.31	0.18
rel–non-rel	0.36	0.52	0.84	0.64
B3LYP–PBE	0.93	0.99	0.83	0.84
PBE–M06-2X	0.50	0.59	0.44	0.44
B3LYP–M06-2X	0.56	0.58	0.59	0.57
double-ζ–triple-ζ	0.14	0.06	0.65	0.18

When we consider all the X–H bond lengths formed by H with any atom, we notice that, on average, the obtained results varied more noticeably with the parameters/conditions of a–d than in the case of the HAR TM–H bond lengths. In spite of the fact that MDs calculated for X–H bond lengths obtained as a result of HARs performed with parameters/conditions of a–d (see Table 4) are 1–2 orders of magnitude lower than it is in the case of analogous MDs obtained for TM–H bond lengths (thus, they are seemingly closer to one another), given much higher sizes of the compared samples, differences between MDs calculated for X–H bond lengths for X–H bonds are statistically significant for parameters/conditions of a–d (see *p* values obtained in Welch’s test in Table 5). When B3LYP-PBE and PBE-M062X are compared pairwise, there are no statistically significant differences in terms of average X–H bond lengths. The “cluster”/“no cluster” and “rel”/“non-rel” MDs have opposite signs and are statistically significantly different, which suggests that one HAR technique tends to overestimate X–H bond lengths and the other tends to underestimate them although these effects are rather small. The difference between the “cluster”/“no cluster” refinements is statistically significant, and using a cluster of multipoles provides slightly better MAD and much better MD/csd and MAD/csd values for X–H bond lengths. Therefore, from here on, all the refinements presented hereafter will be the “cluster” ones performed with DiSCaMB, the B3LYP functional without relativistic effects, and the basis set providing the best results unless otherwise stated. The results obtained with the remaining combinations of parameters and methods are presented in Tables S11–S28.

**Table 4:** Values of MD, MD Calculated for Differences Divided by csd (MD/csd), MAD, and MAD Calculated for Differences Divided by csd (MAD/csd) between X-ray(HAR) and Neutron X–H Bond Lengths with the Corresponding Sample Standard Deviations and Averaged csd’s for Different Conditions: (a) Including or Not In-Crystal Interactions via a Cluster of Multipoles (Cluster/No Cluster), (b) Relativistic Effects, (c) Different DFT Functionals, and (d) the Difference between Double-ζ and Triple-ζ Basis Sets

	MD [Å]	σ [Å]	MD/csd	σ	MAD [Å]	σ [Å]	MAD/csd	σ	mean csd [Å]
cluster	0.004	0.039	0.01	1.15	0.029	0.026	0.89	0.73	0.036
no cluster	–0.004	0.046	–1.30	6.60	0.034	0.032	2.03	6.41	0.038
non-rel	0.002	0.042	–0.17	2.81	0.031	0.028	1.05	2.61	0.037
rel	–0.001	0.042	–0.40	3.77	0.031	0.028	1.23	3.59	0.037
B3LYP	–0.001	0.043	–0.65	4.78	0.031	0.029	1.46	4.60	0.037
PBE	–0.002	0.046	–1.26	6.74	0.034	0.032	2.10	6.53	0.037
M062X	0.001	0.040	–0.06	1.12	0.030	0.027	0.87	0.70	0.037
double-ζ	0.010	0.040	0.23	1.04	0.031	0.028	0.82	0.67	0.041
triple-ζ	0.002	0.043	0.01	1.11	0.031	0.030	0.83	0.73	0.040

**Table 5:** Results of Welch’s T-Test in which the Zero Hypothesis (H_0_) was the Equality of MD, MD Calculated for Differences Divided by csd (MAD/csd), MAD, and MAD Calculated for Differences Divided by csd (MAD/csd) between X-ray(HAR) and Neutron X–H Bond Lengths with the Corresponding Sample Standard Deviations and Averaged csd’s between Different Conditions: (a) Including or Not In-Crystal Interactions via a Cluster of Multipoles (Cluster/No Cluster), (b) Relativistic Effects, (c) Different DFT Functionals, and (d) the Difference between Double-ζ and Triple-ζ Basis Sets

	*p* value			
	MD	MD/csd	MAD	MAD/csd
cluster–no cluster	0.000	0.000	0.000	0.000
rel–non-rel	0.000	0.000	0.604	0.000
B3LYP–PBE	0.141	0.000	0.001	0.000
PBE–M06-2X	0.119	0.000	0.041	0.000
B3LYP–M06-2X	0.003	0.000	0.000	0.000
double-ζ–triple-ζ	0.000	0.000	0.577	0.699

## TM–H Bond Lengths in Various Structures

The refined structures can be divided into three groups based on the quality of the TM–H bond lengths obtained from HAR. The first group (Figure 1), including QOSZON, NOBBOX, and SITKUB, consists of structures in which all or almost all hydrogen atoms can be refined anisotropically. For these compounds, in every case, significant improvement of the positions of hydrogen atoms bonded to TMs was obtained with HAR, compared to the IAM. The HAR bond lengths were elongated considerably toward the neutron lengths such that both values differed by no more than one HAR standard deviation in three cases (Fe-H1 in QOSZON, Ru-HA in NOBBOX, and Rh-H1 in SITKUB) and less than two standard deviations in one case (Fe-H2 in QOSZON), which is an excellent result. The structures and the results obtained for them have already been presented in our communication discussing the preliminary results for a smaller number of structures.^27^Table 6 presents the neutron, IAM, and HAR TM–H and Si–H bond lengths. For QOSZON, the results of HAR with the cc-pVTZ basis set and isotropically refined hydrogen atoms bonded to Fe (H1 and H2) are presented since, in this case, the triple-ζ basis set was superior to the double-ζ one and also H1 and H2 could not be refined anisotropically. The differences between the neutron bond lengths for Fe-H1 and Fe-H2 bonds equal −0.007 and −0.016 Å, respectively, which is within 1 HAR esd/4 neutron esd and 2 HAR esd/8 neutron esd. Compared to the IAM level of underestimation (−0.089 Å for Fe-H1 and −0.101 Å for Fe-H2), HAR brings significant improvement in the position of the H atoms bonded to Fe. In the case of NOBBOX, HAR performed even better since the Ru–HA bond length was underestimated by only 0.005 Å, which is within 1 HAR/2 neutron esd (compared to the IAM, which underestimated this bond length by 0.038 Å). Similarly, the Si–HA bond length was underestimated by HAR by only 0.006 Å, which was within 1 HAR/2 neutron esd (compared to the IAM, which underestimated this bond length by 0.064 Å). In the case of NOBBOX, switching the DFT functional from B3LYP to PBE allows us to obtain the Ru–HA bond length underestimated by only 0.001 Å compared to the neutron value, which is an almost perfect match (see Table S15). The structure of SITKUB is an interesting example of how choosing a different basis set may considerably improve the obtained results. The previously published results^27^ were obtained with HAR using the cc-pVTZ-DK basis set, which improved the IAM length of the Rh-H1 bond (1.40(2) Å) to a certain extent (elongation to 1.433(13) Å with HAR) compared to the neutron bond length of 1.531(11) Å. This means that, whereas the IAM underestimated the Rh–H1 bond length by 0.131 Å, HAR decreased this difference (0.098 Å), which was still far from the neutron result. However, if the jorge-DZP basis set was used instead of cc-pVTZ-DK, HAR elongates the Rh-H1 bond to 1.534(15) Å (see Table 6), which means that the difference between the neutron and the HAR bond length decreased to only 0.003 Å, being within 1 neutron/1 HAR esd. Although the smaller basis set performs so well in terms of hydrogen positions, it is not equally effective when it comes to anisotropic refinement of hydrogen thermal motions. When switching to the jorge-DZP basis set, four hydrogen atoms have to be refined isotropically (see Table 6). Still, the conclusion resulting from studies^2,7^ that the model of the hydrogen thermal motion used for refinement does not change hydrogen positions holds true. In the case of QOSZON and NOBBOX, esd’s for TM–H bond lengths obtained with HAR and IAM are 1 order of magnitude higher than the esd’s from neutron diffraction. In the case of SITKUB, all the esd’s of the Rh–H1 bond length are of the same order of magnitude. On average though, the esd’s from HAR tend to be slightly lower than the IAM ones. For QOSZON and SITKUB, the agreement of the neutron TM–H bond lengths with the values from geometry optimization is very high.

**Table 6:** TM-H and Si-H Bond Lengths (Unit: Å) and Laplacian of the Electron Density Δρ (Unit: e/Å^5^) at the Bond Critical Point Obtained with Various Experimental Methods and Refinement Types for the Three Structures Anisotropically Refined with HAR (“anis” Stands for “Anisotropic” and “iso” Stands for “Isotropic”)^a^

structure	bond	basis set	H ADPs in HAR	neutron	IAM	HAR	optimized	Δρ
QOSZON	Fe–H1	cc-pVTZ	anis + H1, H2 - iso	1.529(2)	1.44(2)	1.522(15)	1.531	4.305
	Fe–H2			1.521(2)	1.42(2)	1.505(14)	1.522	4.217
NOBBOX	Ru–HA	cc-pVTZ-DK	anis	1.598(3)	1.55(2)	1.593(11)	NA	1.605^b^
	SiA–HA			1.874(3)	1.82(2)	1.868(13)	NA	
SITKUB	Rh–H1	jorge-DZP	anis + H, H9A, H10, H13 - iso	1.531(11)	1.40(2)	1.534(15)	1.531	–4.646

a HAR was closer to neutron than IAM for all the presented bonds. Optimized values were obtained as a result of DFT/B3LYP geometry optimization with the jorge-DZP basis set performed using Gaussian16.

b Laplacian calculated for the neutron geometry.

The second group of compounds (Figure 2) includes two compounds (MIGKIY and NEBNEO) for which HAR can be performed only with isotropically refined hydrogen atoms, and the TM–H bond lengths are, on average, improved compared to their values from the IAM. MIGKIY has already been presented in a preceding communication.^74^ In the case of this structure (Table 7), the bond length of only one of the Ru–H bonds was improved after HAR. For the Ru–HA bond length, the difference from the neutron value decreases from 0.05 Å (IAM) to 0.02 Å (HAR), which is already in 3 neutron esd. Unfortunately, the Ru–HB bond length does not change after HAR. Nevertheless, considerable improvement was obtained for the SiA–HA bond; the IAM length was underestimated by 0.117 Å, whereas the HAR length only by 0.017 Å (within 2 neutron esd). For MIGKIY, testing different DFT functionals was also a good method to improve the Ru–H bond lengths; for example, PBE, M06-2X, and M06-2X with a DKH2 relativistic correction slightly improved the position of the HB atom and they decreased the distance between the B3LYP-HAR and the neutron result for the Ru–HB bond from 0.117 to 0.107 Å. Moreover, the M06-2X functional also decreased the neutron-HAR difference for the Ru–HA bond to only 0.01 Å, which is within 2 neutron esd. NEBNEO (Table 7) is a structure in which improvement is observed for the Ru1–H2H, Ru1–H11H, and Ru1–H21H bonds with the following changes in the underestimation from the IAM to HAR: from −0.039 to 0.009 Å, from −0.010 to 0.004 Å, and from −0.024 to 0.016 Å. For the Ru1–H1H bond, both the HAR and IAM yield the same result. Deterioration is observed in the case of the Ru1–H12H bond and the Ru1–H22H bond: from −0.023 Å for IAM to 0.037 Å for HAR and from −0.015 Å for IAM to 0.025 Å for HAR, respectively. Likewise, in the case of NEBNEO, it was possible to achieve improvement in the HAR bond lengths via changing the DFT functional (see Table S13). The most even level of improvement of multiple Ru–H bonds was achieved when M06-2X with a DKH2 relativistic correction is used: Ru1–H1H from −0.078 to 0.068 Å, Ru1–H2H from −0.039 to 0.018 Å, Ru1–H11H from −0.010 to 0.007 Å, Ru1–H12H from −0.023 to 0.037 Å, Ru1–H21H from −0.024 to 0.016 Å, and Ru1–H22H from −0.015 to 0.015 Å (only one bond length deteriorated with HAR, one without improvement, and four bond lengths improved). For both discussed compounds, the esd’s of the TM–H bond lengths from the IAM and HAR were comparable, whereas the ones obtained with neutron diffraction were 1 order of magnitude lower. In the case of NEBNEO, only two bond lengths obtained from geometry optimization (Ru1–H1H and Ru1–H2H) are in good agreement with the neutron values; in the case of the remaining four bond lengths (two H_2_ molecules bonded to Ru), there is a discrepancy between the optimized and the neutron values.

The third group of compounds (Figure 3) includes the five compounds for which only isotropic refinement was attainable and for which HAR generally worsens the TM–H bond lengths compared to the IAM: ZEYVAA, GOJNIF, UJABOX, TIWXOP, and XAXMEP. Of these structures, only XAXMEP had been discussed previously.^57^ For these compounds, unlike for the two aforementioned groups of structures, HAR had a tendency to overestimate the TM–H bond lengths (Table 8). The IAM in turn yielded TM–H bond lengths that were surprisingly close to the neutron values. This is especially pronounced for ZEYVAA, TIWXOP, and XAXMEP. For Nb1-H1, Sb1-H1, Os-H1, Os, H3, and Os-H4, the IAM bond lengths are within 1 neutron esd from the neutron values. HAR results in substantially more divergent bond lengths with the following changes in differences with the neutron values: For Nb1–H1, from the IAM, −0.016 Å, to HAR, 0.08 Å; Sb1–H1, from the IAM, 0.01 Å, to HAR, 0.08 Å; Os–H1, from the IAM, −0.006 Å, to HAR, 0.074 Å; Os–H3, from the IAM, 0.011 Å, to HAR, 0.081 Å; Os–H4 from the IAM, 0.004 Å, to HAR, 0.034 Å. The O2–H2 bond is the only one for which HAR performs slightly better than the IAM, yielding a 0.028 Å difference (within 2 neutron esd) compared to the −0.032 Å difference (within 3 neutron esd) obtained with the IAM. In the case of the GOJNIF IAM, the Ni–H bond lengths were within 2 neutron esd from the neutron value, and in the case of HAR bond lengths, the difference increased to 3–4 neutron esd. The Si2–H101 bond in UJABOX was closer to the neutron length than in the case of the IAM (0.001 Å overestimation vs −0.071 Å underestimation). However, the Ru1–H10 bond length was substantially underestimated with the IAM (−0.209 Å), and HAR made the discrepancy with the neutron data even slightly higher (−0.249 Å). The errors of bond-length determination were similar between the IAM and HAR, and for TIWXOP, GOJNIF, and XAXMEP, they were similar to the neutron uncertainties. In the remaining structures, neutron diffraction yielded TM–H bond lengths, which were 1 order of magnitude more precise. For ZEYVAA, GOJNIF, and UJABOX, the neutron and the optimized TM–H bond lengths are in very high agreement. In the case of TIWXOP and XAXMEP, the agreement is slightly lower.

In order to investigate whether the ionic or covalent character of TM–H bonds is a factor deciding the success of HAR at establishing the bond length, the Laplacian of the electron density (Δρ) at the bond critical point (BCP) was calculated for the TM–H bonds. The theoretical electron density was obtained based on the molecular wave function calculated with Gaussian16 using the DFT method with the B3LYP functional combined with the Douglas–Kroll–Hess second-order scalar relativistic Hamiltonian and the jorge-DZP-DKH basis set. Calculations were preceded by geometry optimization except for the structures of NOBBOX and MIGKIY for which geometry optimization was unsuccessful and the neutron geometry was used instead. Topological analysis of the theoretical electron density used to calculate the Laplacian at the BCPs of the TM–H bonds was performed with the Multiwfn 3.8 program,^75^ and the values of the Laplacian are presented in Tables 6–8. It can be observed that all TM–H bond lengths for the structures in Tables 6 and 7 are either clearly ionic or covalent. In Table 8, there are three structures (ZEYVAA, TIWXOP, and XAXMEP) in which the TM–H bonds have a very weak ionic character (small positive values of the Laplacian). Based on these examples it can be concluded that HAR performed well at determining positions of hydrogen atoms bonded to TMs both with ionic and covalent bonds; however, when the character of the bond is weakly ionic, determining the TM–H bond length with HAR seems problematic.

**Table 7:** TM–H and Si–H Bond Lengths (Unit: Å) and Laplacian of the Electron Density Δρ (Unit: e/Å^5^) at the Bond Critical Point Obtained with Various Experimental Methods and Refinement Types for the Two Structures Isotropically Refined with HAR for which HAR Improved TM–H Bond Lengths^a^

structure	bond	basis set	H ADPs in HAR	neutron	IAM	HAR	optimized	Δρ
MIGKIY	Ru–HA	cc-pVTZ-DK	iso	1.600(8)	1.55(4)	**1.58(6)**	NA	1.364^b^
	Ru–HB			1.587(7)	1.47(4)	1.47(7)	NA	–2.647^b^
	SiA–HA			1.737(10)	1.62(4)	**1.72(6)**		
NEBNEO	Ru1–H1H	cc-pVTZ-DK	iso	1.628(4)	1.55(2)	1.55(2)	1.622	–3.249
	Ru1–H2H			1.625(4)	1.586(19)	**1.634(17)**	1.621	–3.299
	Ru1–H11H			1.730(5)	1.72(2)	**1.734(18)**	1.625	–2.396
	Ru1–H12H			1.753(5)	1.73(2)	*1.79(2)*	1.606	–4.081
	Ru1–H21H			1.764(5)	1.74(2)	**1.78(2)**	1.606	–4.163
	Ru1–H22H			1.745(5)	1.73(3)	*1.77(2)*	1.626	–2.458

a Optimized values were obtained as a result of DFT/B3LYP geometry optimization with the jorge-DZP basis set performed using Gaussian16.

b Laplacian calculated for the neutron geometry. Bold – HAR closer to neutron than IAM, italic – HAR further from neutron than IAM, regular – no difference.

**Table 8:** TM–H and Si–H Bond Lengths (Unit: Å) and Laplacian of the Electron Density Δρ (Unit: e/Å^5^) at the Bond Critical Point Obtained with Various Experimental Methods and Refinement Types for the Five Structures Isotropically Refined with HAR for which HAR Did Not Improve TM–H Bond Lengths^a^

structure	bond	basis set	H ADPs in HAR	neutron	IAM	HAR	optimized	Δρ
ZEYVAA	Nb1–H1	cc-pVTZ-DK	iso	1.816(8)	1.80(8)	*1.88(7)*	1.828	0.202
GOJNIF	Ni1–H1B	cc-pVTZ-DK	iso	1.61(2)	1.65(2)	*1.68(3)*	1.610	10.722^b^
	Ni1–H1A			1.61(2)	1.58(2)	*1.66(2)*	1.611	
UJABOX	Ru1–H10	cc-pVTZ-DK	iso	1.559(7)	1.35(11)	*1.31(10)*	1.565	–3.893
	Si2–H11			1.481(5)	1.41(3)	**1.48(3)**	1.486	
TIWXOP	Sb1–H1	jorge-DZP	iso	1.73(2)	1.74(3)	*1.81(2)*	1.692	0.608
XAXMEP	Os–H1	jorge-DZP	iso	1.606(17)	1.60(7)	*1.68(3)*	1.631	0.303
	Os–H2			1.632(15)	1.60(6)	**1.66(5)**	1.632	0.024
	Os–H3			1.599(21)	1.61(5)	*1.68(5)*	1.632	1.128
	Os–H4			1.626(19)	1.63(4)	*1.66(5)*	1.629	0.608

a Optimized values were obtained as a result of DFT/B3LYP geometry optimization with the jorge-DZP basis set performed using Gaussian16.

b The bond paths for Ni1–H1B and Ni1–H1A bonds are symmetrical and adjacent to the extent that the BCPs for both bonds are merged into one BCP and cannot be distinguished. Bold – HAR closer to neutron than IAM, italic – HAR further from neutron than IAM.

## Data and Refinement Quality

Analyzing TM–H bond lengths obtained with HAR and the IAM from X-ray experiments and comparing them to the experimental neutron values begs the question of how strong is the influence of the quality of the experimental data and quality of the model refined against this data on hydrogen positions obtained based on HAR of X-ray data and based on neutron data. In order to answer this question, we ranked the data sets according to the quality of experimental data and the quality of the refined model. The values of all the parameters characterizing each data set and structure taken into account while preparing the ranking are given in Tables S1–S10. The ranking of structures by data quality was based on the data completeness, *R*_int_, and data resolution. Structures were ordered from the “worst” to the “best” according to the given quantity, and the obtained score was equal to their place in the ranking. If a certain quantity taken into account in the ranking procedure was unknown, the structure automatically obtained the lowest position in the ranking with respect to this quantity. The obtained scores were summed for each structure, separately for the X-ray and the neutron structures, in order to determine the position of the structure in the overall data quality X-ray/neutron ranking (the higher the score, the lower the position in the ranking). The X-ray and the neutron rankings were combined in order to obtain an overall X-ray–neutron data quality ranking of structures suitable for drawing conclusions from the comparison of X-ray and neutron TM–H bond lengths. Similarly, the quality of the refinement was evaluated using statistical values such as the goodness of fit, *R*, w*R*_2_, and the range of residual density values (Δρ range). Separate refinement quality rankings were prepared for neutron, IAM, and HAR structures. The rankings were subsequently combined into overall neutron–IAM and neutron–HAR refinement quality rankings. At the end, the data quality rankings and the refinement quality rankings were combined for each structure in order to obtain the final joint data-refinement quality X-ray(HAR)–neutron and X-ray(IAM)–neutron rankings presented in Table 9. All the intermediate rankings are available in the Supporting Information.

**Table 9:** Final Joint Data-Refinement Quality X-ray(HAR)–Neutron and X-ray(IAM)–Neutron Rankings of Structures Together with the Score Obtained by the Structures

X-ray(HAR)–neutron			X-ray(IAM)–neutron		
position	structure	score	position	structure	score
1	NOBBOX	99	1	NOBBOX	100
2	QOSZON	90	2	QOSZON	87
3	GOJNIF	73	3	SITKUB	72
4	SITKUB	70	3	GOJNIF	72
5	NEBNEO	64	4	TIWXOP	66
6	TIWXOP	63	5	NEBNEO	64
7	MIGKIY	60	6	MIGKIY	60
8	UJABOX	57	7	UJABOX	56
9	ZEYVAA	44	8	ZEYVAA	42
10	XAXMEP	39	9	XAXMEP	40

The obtained rankings are only tentative for a few reasons: (a) the differences between parameters are in certain cases very small, (b) all the parameters were considered with equal importance, which might not be optimal, and (c) the parameters taken into account do not exhaust all the factors and circumstances, which could influence the structure and refinement quality. Examples of such circumstances are the following: (a) for XAXMEP, the neutron experiment resulted in a data-to-parameter ratio insufficient to refine the ADPs of hydrogen atoms;^57^ (b) in the X-ray structure of MIGKIY, unmodeled disorder in the vicinity of one of the CF_3_ groups is found (the disorder is separated from the Ru–H bonds, and the related residual density is up to 0.4 e/Å^3^ for F atoms; however, it influences all the reflections and therefore it might also influence the Ru–H bond region); (c) in the neutron data set collected for TIWXOP, the hydrogen atoms of one of the methyl groups were significantly disordered and therefore they had to be refined isotropically.^56^ Nevertheless, the overall ranking of structures correlates with certain trends observed during the analysis of TM–H bonds in the studied structures. The two structures, which achieved the best score, are NOBBOX and QOSZON, which belong to the first group of structures for which anisotropic HAR is achievable and TM–H bond lengths from HAR are in good agreement with those from neutron experiments. Closely behind them is SITKUB for which excellent agreement could also be achieved. Three structures at the bottom of the ranking (XAXMEP, ZEYVAA, and UJABOX) belong to the third group of compounds for which only isotropic HAR was possible and a general deterioration of TM–H bond lengths compared to the IAM was observed. The remaining structures from the middle of the ranking are characterized by quite similar scores, and they belong either to the third group of structures or to the second group for which only isotropic HAR was feasible and still improved TM–H bond lengths were obtained.

## X–H Bond Lengths and TM–H Bond Lengths in View of the Data Quality

The overall evaluation of the quality of all hydrogen positions was also narrowed down to the X–H bond lengths obtained with the DiSCaMB-HAR method, B3LYP functional, and one selected basis set. The evaluation was based on the mean difference (MD) and mean absolute difference (MAD) calculated between the X–H bond lengths obtained with HAR and the ones obtained with neutron diffraction (the calculated values are presented in Table 10). Combined HAR–neutron standard deviations (csd’s) are also presented. All the values were calculated for all X–H bond lengths and for TM–H bond lengths separately. The IAM in a vast majority of the analyzed cases leads to shortened bond lengths, and the average shortening for all structures amounts to MD = −0.122 Å with MAD = 0.122 Å. For DiSCaMB-HAR, the MAD for all X–H bonds averaged over all the structures amounted to 0.029 Å, which was 4 times better than that of IAM. The MD for DiSCaMB-HAR X–H bond lengths was very close to zero, showing that, on average, HAR produced X–H bond lengths that were neither too long nor too short compared to neutron diffraction. Similar conclusions can be drawn when looking at the average values calculated for the TM–H bonds. The IAM mostly underestimated TM–H bond lengths (MD = −0.046 Å; MAD = 0.052 Å), whereas HAR had an equally strong tendency to produce TM–H bond lengths that are either longer or shorter than in the neutron structure (MD = −0.004 Å; MAD = 0.051 Å). Based on the value of the MAD, the accuracy of TM–H bond lengths was very similar in the case of HAR and IAM. The average values of the csd were quite similar for the IAM and HAR (the values obtained for HAR were slightly lower), and the average values of the csd calculated for TM–H bonds did not diverge from those obtained for all X–H bonds.

**Table 10:** Mean Difference (MD; Unit: Å), Mean Absolute Difference (MAD; Unit: Å), and Mean Combined Standard Deviation (mean(csd); Unit: Å) Calculated between All the X–H/TM–H Bond Lengths Obtained with DiSCaMB-HAR (B3LYP Functional and the Most Optimal Basis Set) or the IAM and the Neutron Bond Lengths

			MD				MAD				mean(csd)			
			IAM–neutron		HAR–neutron		IAM–neutron		HAR–neutron		IAM–neutron		HAR–neutron	
	basis set	TM–H	all X–H	TM–H	all X–H	TM–H	all X–H	TM–H	all X–H	TM–H	all X–H	TM–H	all X–H	TM–H
GOJNIF	cc-pVTZ-DK	2 Ni–H	–0.130	0.000	–0.015	0.060	0.131	0.030	0.030	0.060	0.124	0.028	0.031	0.032
MIGKIY	cc-pVTZ-DK	2 Ru–H	–0.118	–0.084	0.000	–0.069	0.118	0.084	0.039	0.069	0.012	0.041	0.048	0.051
NEBNEO	cc-pVTZ-DK	6 Ru–H	–0.129	–0.032	–0.002	0.002	0.129	0.032	0.023	0.028	0.021	0.022	0.021	0.020
NOBBOX	cc-pVTZ-DK	Ru–H	–0.106	–0.048	–0.012	–0.005	0.106	0.048	0.014	0.005	0.004	0.020	0.013	0.011
QOSZON	cc-pVTZ	2 Fe–H	–0.135	–0.095	0.006	–0.012	0.135	0.095	0.011	0.012	0.024	0.020	0.017	0.015
SITKUB	jorge-DZP	Rh–H	–0.116	–0.131	0.017	0.003	0.116	0.131	0.023	0.003	0.018	0.023	0.021	0.019
TIWXOP	jorge-DZP	Sb–H	–0.131	0.010	0.016	0.090	0.132	0.010	0.040	0.090	0.053	0.036	0.051	0.028
UJABOX	cc-pVTZ-DK	Ru–H	–0.164	–0.209	–0.029	–0.249	0.164	0.209	0.045	0.249	0.047	0.110	0.047	0.100
XAXMEP	jorge-DZP	4 Os–H	–0.099	–0.006	0.028	0.054	0.100	0.018	0.046	0.054	0.027	0.058	0.064	0.049
ZEYVAA	cc-pVTZ-DK	Nb–H	–0.133	–0.016	0.005	0.064	0.133	0.016	0.038	0.064	0.067	0.080	0.073	0.070
all structures			–0.122	–0.046	0.002	0.004	0.122	0.052	0.029	0.051	0.038	0.039	0.035	0.035

**Figure 4:**
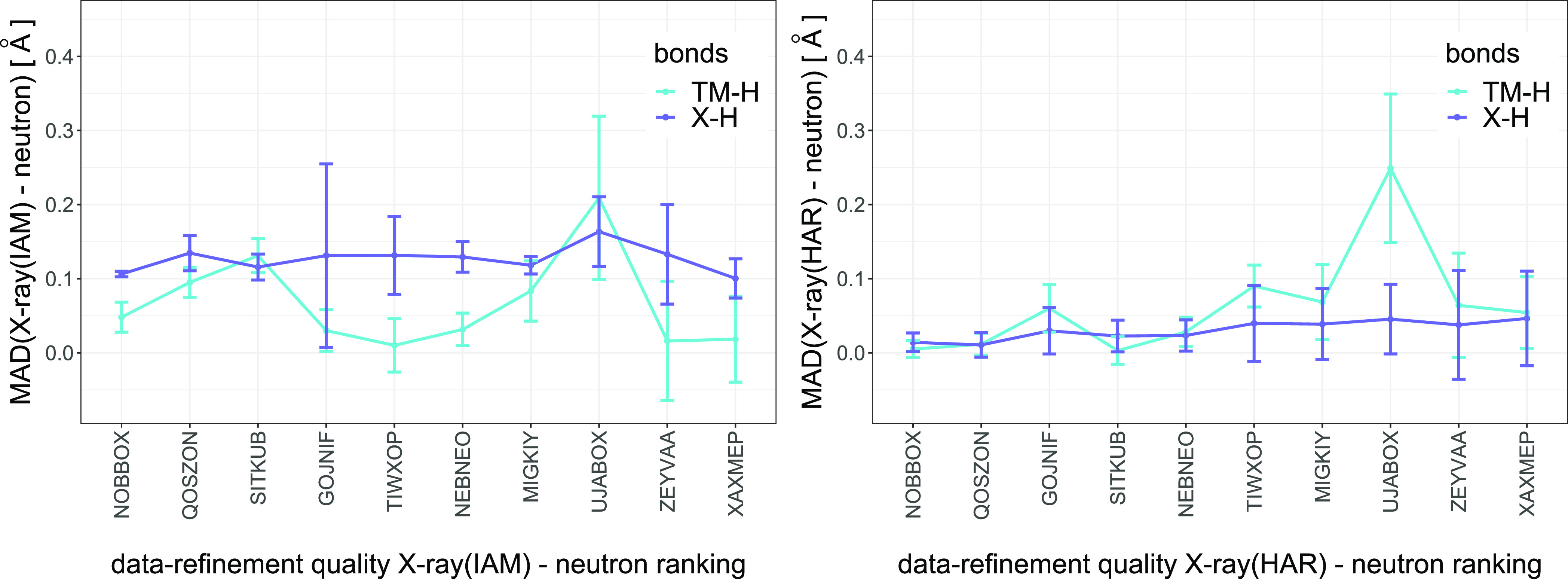
Mean absolute differences between X-ray and neutron TM–H and X–H bond lengths (left: X-ray(IAM); right: X-ray(HAR)) arranged according to the corresponding data-refinement quality X-ray(IAM)–neutron and X-ray(HAR)–neutron rankings. Error bars denote the csd.

It should not be disregarded that MAD displays a certain variability among the analyzed structures, which we decided to investigate in view of the data-refinement quality ranking (see Figure 4 and Table 10). Trends are clearly visible in the case of HAR. For X–H bond lengths, it can be observed that the discrepancy between the X-ray(HAR) and the neutron values (measured as the MAD) increases with a decreasing position in the data-refinement quality and X-ray(HAR)–neutron ranking. For the highest-quality data sets, NOBBOX and QOSZON, it amounted to 0.014 and 0.011 Å, which was the level of the average difference between HAR and neutron lengths of typical bonds in crystals of organic compounds.^6^ For the next group of compounds, GOJNIF, SITKUB, and NEBNEO, the MAD slightly increased, assuming values between 0.023 and 0.029 Å. The next group of compounds located lower in the ranking consists of TIWXOP, MIGKIY, and ZEYVAA with an MAD between 0.038 and 0.040 Å. For XAXMEP and UJABOX, the MADs were slightly higher (0.046 and 0.045 Å, respectively). When we restricted our considerations to TM–H bond lengths only, we can also observe the general trend of an increasing MAD with a decreasing position in the data-refinement quality X-ray(HAR)–neutron ranking. There was a tendency that became visible for the lower-quality structures that the MAD for TM–H bonds was higher than the MAD calculated for X–H bonds for the same structure. The exceptions from this rule (NOBBOX and SITKUB) are structures ranked closer to the top of the ranking. In the case of QOSZON, the MAD for TM–H bonds was higher by only 0.001 Å than the MAD for X–H bonds. For these three structures, it was possible to achieve TM–H bond lengths closer to the neutron value than 0.014 Å, which is the level of the average difference between HAR and neutron lengths observed for typical X–H bonds in organic compounds.^6^ The highest increase in the MAD for TM–H bonds compared to X–H bonds was observed for UJABOX for which the Ru–H bond diverges considerably from the neutron value. The MAD plotted for the data-refinement quality X-ray(IAM)–neutron did not show clear dependence on the position in the ranking. The X–H bonds of all the structures were characterized by an MAD equal to at least 0.100 Å with the lowest value obtained for the last structure in the ranking, XAXMEP. The highest value of the MAD for X–H bonds was equal to 0.164 Å for UJABOX, which is the only result in line with the results obtained for HAR. In the case of TM–H bond lengths obtained with IAM, the MAD was generally lower than the one obtained for all X–H bonds (the only exceptions are SITKUB and UJABOX), which was a very surprising observation given the higher difficulty of establishing the position of H atoms in the vicinity of TMs. What is even more surprising is that the MAD is particularly low for structures such as TIWXOP (from the middle of the ranking) and ZEYVAA and XAXMEP (two structures from the bottom of the ranking), attaining low values typical for X–H bond lengths refined with HAR, whereas the remaining X–H bond lengths for these structures are far from the neutron lengths as in typical IAM refinements. The averaged csd’s calculated for HAR (both TM–H and X–H bonds) had a tendency to increase with a decreasing position in the ranking, the same conclusion held true in the case of TM–H bond lengths obtained with the IAM. In the case of the IAM X–H bond lengths, such a tendency was not observed.

The main conclusion that emerged from plotting the MAD against the data-refinement quality rankings was that the performance of HAR was dependent on the quality of the structure; for the data sets of the highest quality, the TM–H and X–H bond lengths obtained with HAR were in very good agreement with the neutron values. With a deteriorating structure quality, the agreement of bond lengths with the neutron structure also decreased. For the high-quality structures, HAR was significantly better at establishing positions of hydrogen atoms in the vicinity of transition metals than the IAM. For the structures of the worst quality, this trend was reversed as the IAM yielded TM–H bond lengths in the best agreement with the neutron data for the two structures ranked as the worst. Another non-intuitive observation was that the IAM in majority of the cases determined the TM–H bond lengths with better accuracy than the X–H bond lengths in the given structure. Based on these results, we can conclude that the more sophisticated model of electron density applied in HAR improved TM–H bond lengths only when the quality of the analyzed data was high. In the case of lower data quality, HAR still improved the positions of hydrogen atoms bonded to lighter atoms; however, it did not improve bond lengths for hydrogen atoms bonded to heavy metals.

**Figure 5:**
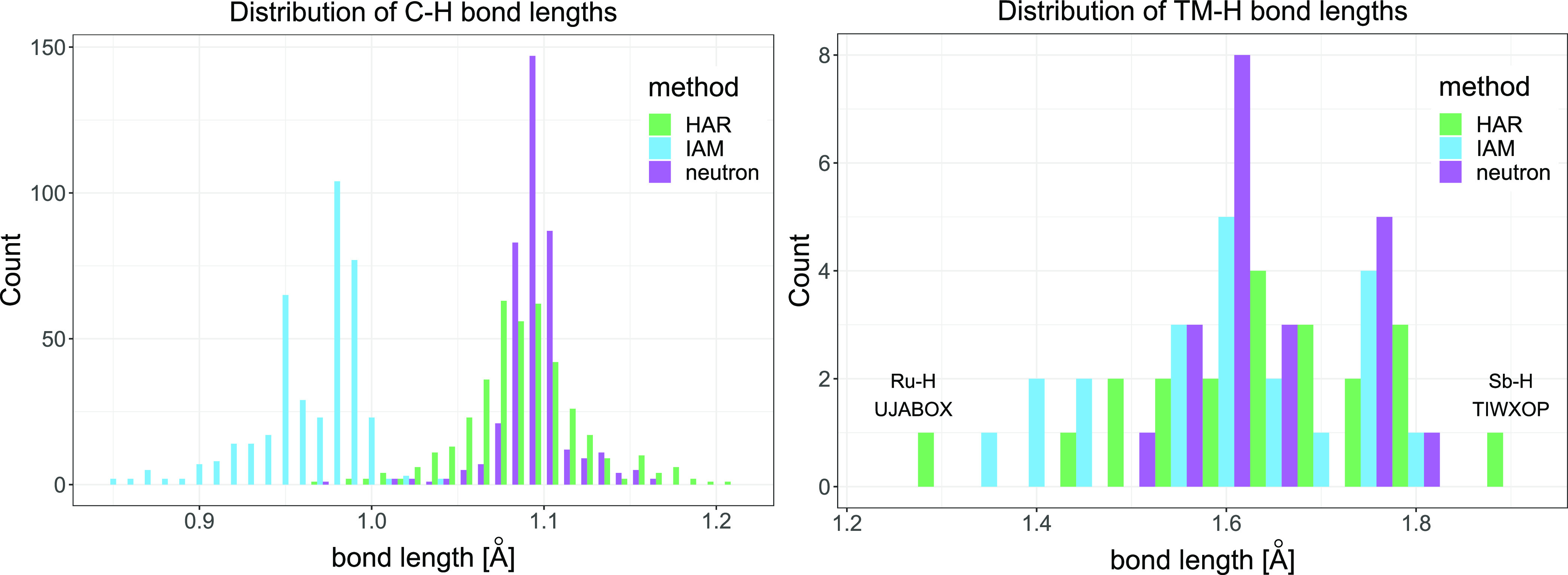
Histograms showing the distribution of C–H (left) and TM–H (right) bond lengths obtained with DiSCaMB-HAR (B3LYP functional and the most optimal basis set) or the IAM and the neutron bond lengths.

The observations resulting from the analysis of MD and MAD based on Figure 4 and Table 10 are confirmed by histograms of C–H and TM–H bonds in all the analyzed compounds obtained with neutron diffraction, the IAM, and HAR (Figure 5). The Si–H bonds present in the structures are excluded from the histograms for the sake of clarity since their lengths strongly diverge from typical C–H bond lengths. There are only three Si–H bonds, and for all of them, HAR in comparison with the IAM resulted in much better agreement with the neutron bond lengths (Tables 6–8). In the case of C–H bond lengths, the distribution of IAM bond lengths is visibly separated from the distributions obtained for the other methods and shifted toward shorter bond lengths. The maximum of the IAM distribution is shifted by 0.12 Å from the maxima of the HAR and neutron distributions, which are located at the same position. The IAM histogram is less symmetrical than the HAR and neutron histograms. The distribution obtained for HAR is flatter than the neutron one, and it is characterized by a higher occurrence of extreme values and a longer right tail. The histograms obtained for the TM–H bond lengths are based on much fewer observations and more diverse bond types; therefore, their interpretation is less straightforward. The maxima of the distributions obtained with all methods have similar positions; however, as it is in the case of C–H bond lengths, the maximum for the neutron bond lengths is the highest. Moreover, the distribution of neutron bond lengths is the narrowest and shifted toward higher lengths. In the case of the IAM, shorter bond lengths appear in the distribution. It is similar for HAR, which is additionally characterized by the outlying shortest bond length Ru–H in UJABOX and the longest Sb–H in TIWXOP.

## Atomic Thermal Motions

As it was stated in our recently published study,^74^ performing HAR with hydrogen thermal motions estimated with SHADE2 was achievable only for QOSZON and, in terms of the Fe–H bond lengths, it did not introduce much improvement. Comparison with neutron thermal ellipsoids was not possible owing to different experimental temperatures of the neutron and X-ray measurements. Therefore, in this study, we would like to focus on two structures for which the neutron and X-ray experiments were performed at the same temperature, which are GOJNIF (experimental temperature: 100 K) and TIWXOP (experimental temperature: 120 K). For both of them, only isotropic HAR was successful, and thus isotropic displacement parameters of hydrogen atoms from HAR are compared to the equivalent isotropic displacement parameters from the neutron measurements. Additionally, equivalent isotropic displacement parameters for non-hydrogen atoms are analyzed using the mean difference (MD), mean absolute difference (MAD), and combined standard deviation (csd) calculated between the given X-ray structure and the neutron structure.

**Figure 6:**
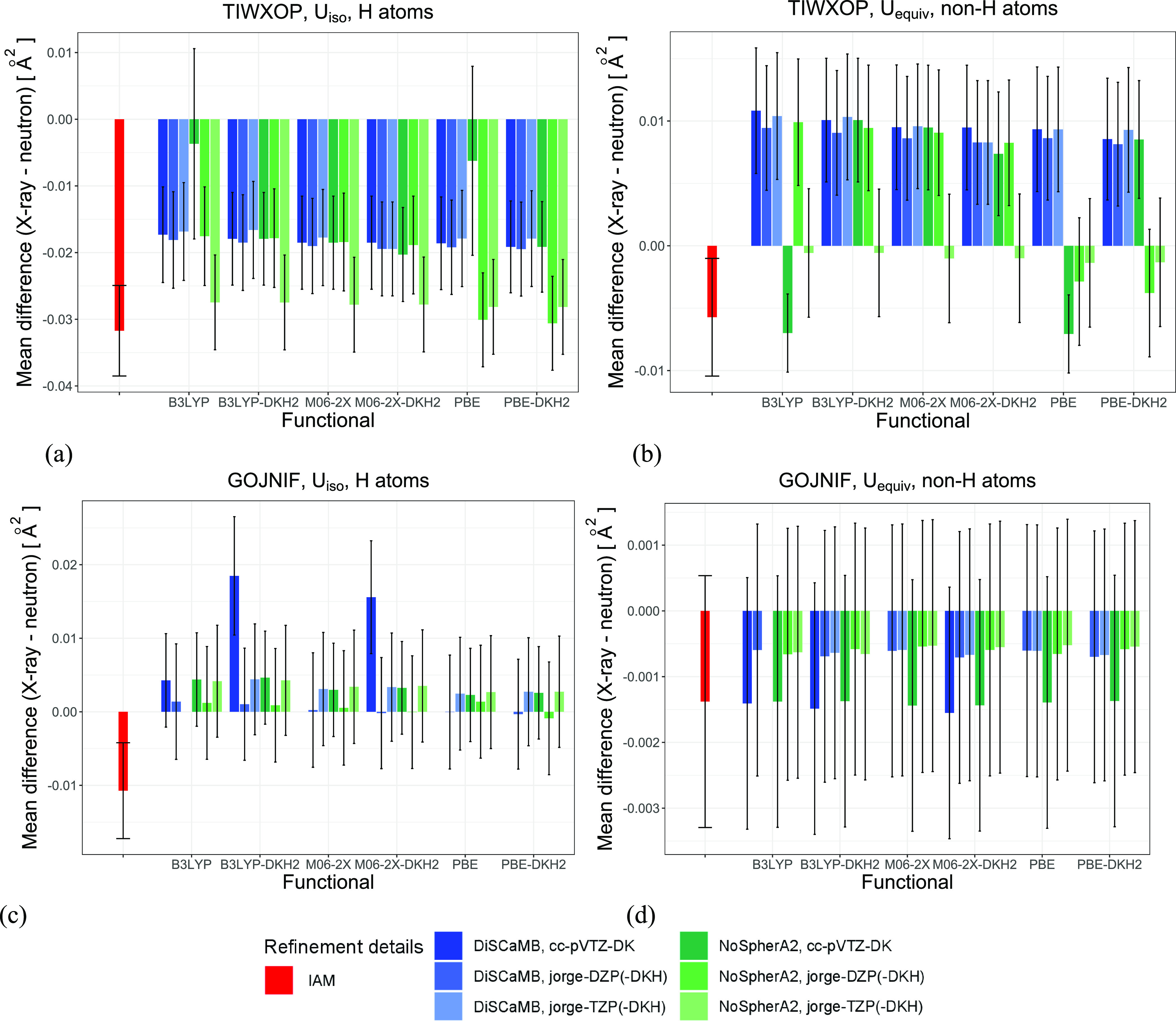
Mean difference (MD) between the X-ray-derived values of *U*_iso_ of hydrogen atoms (*U*_equiv_ of non-hydrogen atoms) and the neutron-derived values of *U*_equiv_: (a) TIWXOP, hydrogen atoms; (b) TIWXOP, non-hydrogen atoms; (c) GOJNIF, hydrogen atoms; and (d) GOJNIF, non-hydrogen atoms. Error bars depict the mean combined neutron and X-ray standard deviation.

Plots presenting the MD and MAD calculated between the X-ray-derived values of the *U*_iso_ of hydrogen atoms (*U*_equiv_ of non-hydrogen atoms) and the neutron-derived values of the *U*_equiv_ with the mean csd are given in Figure 6 and Figure S1. As the analysis of MD shows, for both compounds, the IAM underestimates *U*_iso_/*U*_equiv_ for all atoms. The underestimation was stronger for TIWXOP than for GOJNIF and stronger for hydrogen atoms than for non-hydrogen atoms. In the case of HAR, whether thermal motions were, on average, under- or overestimated depended on whether hydrogen or non-hydrogen atoms were discussed, and in certain cases, it also depended on the refinement strategy. For TIWXOP, the MAD plots show that clearly, there is a discrepancy between the values of *U*_iso_/*U*_equiv_ obtained with HAR and those derived from neutron diffraction, and the divergence is higher than 3 mean csd. In the case of TIWXOP, for the majority of refinement hydrogen atoms, the MAD values obtained with HAR were slightly lower than those obtained with the IAM for hydrogen atoms and slightly higher than the IAM MAD for non-hydrogen atoms. However, there is a group of HARs (performed with NoSPherA2 and the jorge-TZP(-DKH) basis sets and also all refinements performed with NoSPherA2, PBE(-DKH2) functionals, and the jorge-DZP(-DKH) basis sets) for which the MAD values for H atoms were slightly higher but still lower than the values obtained with the IAM. For both H atoms and non-H atoms, the NoSpherA2 refinement with the cc-pVTZ-DK basis set and B3LYP or PBE functional was characterized by lower disagreement with the neutron *U*_iso_/*U*_equiv_.

The discrepancies between the X-ray and neutron thermal displacement parameters for GOJNIF were much lower than they were for TIWXOP. For non-H atoms, the discrepancies were so small that all MDs were within 1 mean csd from 0 and MADs were either within 1 mean csd from 0 or slightly further. For non-H atoms in GOJNIF, the MDs and MADs obtained with the IAM were very similar to those obtained from HAR performed with the cc-pVTZ-DK basis set. The remaining HARs yielded slightly better agreement with the neutron thermal motions. For hydrogen atoms in GOJNIF, the majority of HARs yielded slightly lower MADs than the IAM. There were however some versions of HAR (DiSCaMB, cc-pVTZ-DK, B3LYP-DKH2, and M06-2X-DKH2) for which the MAD for hydrogen atoms was higher than for the IAM. For the same versions of HAR, higher discrepancies were also observed in the MD plot. It is worth pointing out that the MD obtained for hydrogen atoms with the IAM for GOJNIF is negative, whereas the MD for HARs was mostly positive (and in majority of the cases, it was within 1 csd from 0). To summarize, the level of similarity of *U*_iso_/*U*_equiv_ obtained with HAR depends on the analyzed crystal structure and, as it could be expected, the similarity of thermal motions is lower for hydrogen atoms. The results for various versions of HAR are similar with a few exceptions, and they are also quite comparable with the IAM.

## Conclusions

In summary, we performed an extensive study of the Hirshfeld atom refinement approach applied to the problem of obtaining accurate positions of hydrogen atoms bonded to heavy metals based on the X-ray structures of 10 transition metal-bound hydrides. The X-ray bond lengths were compared to the values resulting from neutron diffraction and to those obtained in the course of the IAM refinement. Many varieties of HAR were explored: with and without modeling the crystal environment with a cluster of atomic multipoles, with and without relativistic effects, a selection of three DFT functionals, and various basis sets. In the case of TM–H bond lengths, no statistically significant differences between the mean bond-length discrepancies from neutron values obtained with various refinement parameters were observed. Although the differences were smaller if all bonds formed by hydrogen atoms were considered, they were statistically significant in the case of modeling/not modeling the crystal environment with a cluster of atomic multipoles, using/not using relativistic effects, switching between B3LYP and M06-2X functionals, and changing from a double-ζ to a triple-ζ basis set. The mean differences calculated for X–H bonds obtained with various types of HAR differed from one another (also with their sign). The mean absolute differences were very similar, meaning that all types of HAR give a similar level of deviation from neutron X–H bond lengths and no type could be selected as superior with the exception of slightly better performance of HAR with a cluster of atomic multipoles. Further, we discussed in detail the results obtained for the structures studied based on non-relativistic HARs performed with the B3LYP functional and taking into account interactions in a crystal and the basis set of choice. Despite the fact that, on average, no significant differences were observed between the tested varieties of HAR with respect to TM–H bond lengths, for each structure, specific adjustments helped in considerably improving the TM–H bond lengths.

For three of the investigated structures (QOSZON, NOBBOX, and SITKUB) for which anisotropic HAR was attainable, the obtained TM–H bond lengths were in very high agreement with the neutron values. The discrepancies were on the level of a few milliangstrom, and moreover, by adjusting some parameters of HAR, it was possible to obtain substantial improvement in the TM–H bond lengths. For two other compounds (MIGKIY and NEBNEO), only isotropic HAR was possible; however, the TM–H bond lengths were still in better agreement with the neutron values than in the case of the IAM. Additionally, for these two structures, the TM–H bond lengths could be improved by changing the functional or introducing relativistic effects into the wave function calculations. Unfortunately, for the remaining five compounds the TM–H bond lengths were, on average, deteriorated by HAR compared to the IAM, which yielded TM–H bond lengths in surprisingly good agreement (better than in the case of other bonds formed by hydrogen atoms) with the neutron values. The structures could be ranked according to data and refinement quality of the corresponding X-ray and neutron data set. The ranking revealed a clear trend for HAR: with increasing the data and refinement quality (increasing the position in the data-refinement quality X-ray–neutron ranking), the agreement of both TM–H and X–H bond lengths from HAR with the neutron diffraction data also increased. In particular, it could be observed that HAR considerably improved the IAM TM–H bond lengths for the top structures from the ranking, making them almost perfectly match the neutron distances. Anisotropic refinement of hydrogen thermal motions was also possible with HAR for the structures with higher rankings. A conclusion can be drawn that the advanced model of electron density employed by HAR is superior to the spherical model of density that is high quality for data sets. For low-quality data, a more exact model of electron density was not beneficial, which was confirmed by the fact that, for the structures that were ranked at lower positions, HAR yielded worse TM–H distances, compared to the IAM. The good performance of the IAM for low-quality data seems to be rather accidental since the IAM produces TM–H distances that are in the best agreement with the X-ray data for the two structures at the bottom of the ranking. Quite an unexpected observation was that the IAM for the majority of the structures produced TM–H bond lengths that were in better agreement with the neutron bond lengths than in the case of hydrogen atoms bonded to lighter atoms. The IAM usually underestimated TM–H and X–H bond lengths. HAR yielded both under- and overestimated TM–H and X–H bond lengths with similar frequencies. The precision of TM–H and X–H bond lengths was similar for the IAM and HAR.

The last problem discussed in our study was a direct comparison of neutron and X-ray isotropic (equivalent) displacement parameters for two structures for which neutron and X-ray data were measured at the same temperatures. For both structures, the IAM underestimated the *U*_iso_/*U*_equiv_ for all atoms and this effect is stronger for hydrogen atoms than for non-hydrogen atoms. HAR, depending on the case, either under- or overestimated the *U*_iso_/*U*_equiv_ for hydrogen and non-hydrogen atoms. Based on the results obtained for only two structures, it was impossible to unambiguously conclude whether HAR or the IAM provided better agreement between the X-ray thermal motions and the neutron data. The differences between HAR and the IAM were often small, and they depended on the structure and the type of HAR performed.

This analysis of 10 structures of metal–organic complexes with hydrogen atoms bonded to transition metals shows how important it is to collect high-quality X-ray data so that a sophisticated model of electron density, such as the one used by HAR, could reach its full potential. Although no specific recommendation can be given as to which parameters of HAR (surrounding with a cluster of multipoles, relativistic effects, functionals, and basis sets) should be used to achieve the best TM–H bond lengths, it is worth investigating which parameters are optimal for the structure under consideration. It must be stressed that accurate hydrogen positions can be achieved with HAR applied to standard-resolution data of sufficient quality. Nevertheless, it remains an open question whether performing HAR against high-resolution data could improve TM–H bond lengths and how different strategies improving the accuracy of wave function calculations could influence the final result.

## Supporting Information Available

Experimental and refinement details for the crystal structures, bond lengths for the crystal structures, and various rankings of data and refinement quality (PDF)

## References

[ref1] Compton A. H. The Distribution of the Electrons in Atoms. Nature 1915, 95, 343–344. 10.1038/095343b0.

[ref2] Woińska M.; Grabowsky S.; Dominiak P. M.; Woźniak K.; Jayatilaka D. Hydrogen Atoms Can Be Located Accurately and Precisely by X-Ray Crystallography. Sci. Adv. 2016, 2, e1600192 10.1126/sciadv.1600192. 27386545 PMC4928899

[ref3] Jayatilaka D.; Dittrich B. X-Ray Structure Refinement Using Aspherical Atomic Density Functions Obtained from Quantum-Mechanical Calculations. Acta Crystallogr., Sect. A: Found. Adv. 2008, 64, 383–393. 10.1107/S0108767308005709. 18421128

[ref4] Capelli S. C.; Bürgi H.-B.; Dittrich B.; Grabowsky S.; Jayatilaka D. Hirshfeld Atom Refinement. IUCrJ 2014, 1, 361–379. 10.1107/S2052252514014845. PMC4174878 25295177

[ref5] Hirshfeld F. L. Bonded-Atom Fragments for Describing Molecular Charge Densities. Theor. Chim. Acta 1977, 44, 129–138. 10.1007/BF00549096.

[ref6] Jha K. K.; Gruza B.; Kumar P.; Chodkiewicz M. L.; Dominiak P. M. TAAM: A Reliable and User Friendly Tool for Hydrogen-Atom Location Using Routine X-Ray Diffraction Data. Acta Crystallogr., Sect. B: Struct. Sci., Cryst. Eng. Mater. 2020, 76, 296–306. 10.1107/S2052520620002917. 32831250

[ref7] Malaspina L. A.; Hoser A. A.; Edwards A. J.; Woińska M.; Turner M. J.; Price J. R.; Sugimoto K.; Nishibori E.; Bürgi H.-B.; Jayatilaka D.; Grabowsky S. Hydrogen Atoms in Bridging Positions from Quantum Crystallographic Refinements: Influence of Hydrogen Atom Displacement Parameters on Geometry and Electron Density. CrystEngComm 2020, 22, 4778–4789. 10.1039/D0CE00378F.

[ref8] Woinska M.; Wanat M.; Taciak P.; Pawinski T.; Minor W.; Wozniak K. Energetics of Interactions in the Solid State of 2-Hydroxy-8-X-Quinoline Derivatives (X = Cl, Br, I, S-Ph): Comparison of Hirshfeld Atom, X-Ray Wavefunction and Multipole Refinements. IUCrJ 2019, 6, 868–883. 10.1107/S2052252519007358. PMC6760436 31576220

[ref9] Schlapbach L.; Züttel A. Hydrogen-Storage Materials for Mobile Applications. Nature 2001, 414, 353–358. 10.1038/35104634. 11713542

[ref10] Fukuzumi S.; Suenobu T. Hydrogen Storage and Evolution Catalysed by Metal Hydride Complexes. Dalton Trans. 2013, 42, 18–28. 10.1039/C2DT31823G. 23080061

[ref11] Langmi H. W.; Ren J.; North B.; Mathe M.; Bessarabov D. Hydrogen Storage in Metal-Organic Frameworks: A Review. Electrochim. Acta 2014, 128, 368–392. 10.1016/j.electacta.2013.10.190.

[ref12] Semenok D. V.; Kruglov I. A.; Savkin I. A.; Kvashnin A. G.; Oganov A. R. On Distribution of Superconductivity in Metal Hydrides. Curr. Opin. Solid State Mater. Sci. 2020, 24, 100808 10.1016/j.cossms.2020.100808.

[ref13] Du M.; Zhang Z.; Song H.; Yu H.; Cui T.; Kresin V. Z.; Duan D. High-Temperature Superconductivity in Transition Metallic Hydrides MH11 (M = Mo, W, Nb, and Ta) under High Pressure. Phys. Chem. Chem. Phys. 2021, 23, 6717–6724. 10.1039/D0CP06435A. 33710184

[ref14] Skoskiewicz T. Superconductivity in the Palladium-Hydrogen and Palladium-Nickel-Hydrogen Systems. Phys. Status Solidi A 1972, 11, K123–K126. 10.1002/pssa.2210110253.

[ref15] Li X.; Peng F. Superconductivity of Pressure-Stabilized Vanadium Hydrides. Inorg. Chem. 2017, 56, 13759–13765. 10.1021/acs.inorgchem.7b01686. 29094931

[ref16] Yu S.; Jia X.; Frapper G.; Li D.; Oganov A. R.; Zeng Q.; Zhang L. Pressure-Driven Formation and Stabilization of Superconductive Chromium Hydrides. Sci. Rep. 2015, 5, 17764 10.1038/srep17764. 26626579 PMC4667211

[ref17] Hubbard Horn F.; Ziegler W. T. Superconductivity and Structure of Hydrides and Nitrides of Tantalum and Columbium^1,2^. J. Am. Chem. Soc. 1947, 69, 2762–2769. 10.1021/ja01203a056.

[ref18] Satterthwaite C. B.; Toepke I. L. Superconductivity of Hydrides and Deuterides of Thorium. Phys. Rev. Lett. 1970, 25, 741–743. 10.1103/PhysRevLett.25.741.

[ref19] Bullock R. M. Catalytic Ionic Hydrogenations. Chem. – Eur. J. 2004, 10, 2366–2374. 10.1002/chem.200305639. 15146510

[ref20] Rakowski Dubois M.; Dubois D. L. Development of Molecular Electrocatalysts for CO_2_ Reduction and H_2_ Production/Oxidation. Acc. Chem. Res. 2009, 42, 1974–1982. 10.1021/ar900110c. 19645445

[ref21] Thoi V. S.; Sun Y.; Long J. R.; Chang C. J. Complexes of Earth-Abundant Metals for Catalytic Electrochemical Hydrogen Generation under Aqueous Conditions. Chem. Soc. Rev. 2013, 42, 2388–2400. 10.1039/C2CS35272A. 23034627

[ref22] Hilt G. Double Bond Isomerisation and Migration—New Playgrounds for Transition Metal-Catalysis. ChemCatChem 2014, 6, 2484–2485. 10.1002/cctc.201402341.

[ref23] Lyons T. W.; Sanford M. S. Palladium-Catalyzed Ligand-Directed C–H Functionalization Reactions. Chem. Rev. 2010, 110, 1147–1169. 10.1021/cr900184e. 20078038 PMC2836499

[ref24] Labinger J. A.; Bercaw J. E. Understanding and Exploiting C–H Bond Activation. Nature 2002, 417, 507–514. 10.1038/417507a. 12037558

[ref25] Choi J.; Roy MacArthur A. H.; Brookhart M.; Goldman A. S. Dehydrogenation and Related Reactions Catalyzed by Iridium Pincer Complexes | Chemical Reviews. Chem. Rev. 2011, 111, 1761–1779. 10.1021/cr1003503. 21391566

[ref26] Kleemiss F.; Dolomanov O. V.; Bodensteiner M.; Peyerimhoff N.; Midgley L.; Bourhis L. J.; Genoni A.; Malaspina L. A.; Jayatilaka D.; Spencer J. L.; White F.; Grundkötter-Stock B.; Steinhauer S.; Lentz D.; Puschmann H.; Grabowsky S. Accurate Crystal Structures and Chemical Properties from NoSpherA2. Chem. Sci. 2021, 12, 1675–1692. 10.1039/D0SC05526C. PMC8179328 34163928

[ref27] Woińska M.; Chodkiewicz M. L.; Woźniak K. Correction: Towards Accurate and Precise Positions of Hydrogen Atoms Bonded to Heavy Metal Atoms. Chem. Commun. 2021, 57, 4469–4469. 10.1039/D1CC90147H. 33890596

[ref28] Holsten S.; Malaspina A. L.; Kleemiss F.; Mebs S.; Hupf E.; Grabowsky S.; Beckmann J. Different Reactivities of (5-Ph_2_P-Ace-6-)_2_MeSiH toward the Rhodium(I) Chlorides [(C_2_H_4_)_2_RhCl]_2_ and [(CO)_2_RhCl]_2_. Hirshfeld Atom Refinement of a Rh–H···Si Interaction. Organometallics 2021, 40, 2027–2038. 10.1021/acs.organomet.0c00804.

[ref29] Jayatilaka D.; Grimwood D. J. Tonto: A Fortran Based Object-Oriented System for Quantum Chemistry and Crystallography. In Computational Science — ICCS 2003; Sloot P. M. A., Abramson D., Bogdanov A. V., Gorbachev Y. E., Dongarra J. J., Zomaya A. Y., Eds.; Lecture Notes in Computer Science; Springer: Berlin, Heidelberg, 2003; pp. 142–151. 10.1007/3-540-44864-0_15.

[ref30] Dolomanov O. V.; Bourhis L. J.; Gildea R. J.; Howard J. A. K.; Puschmann H. OLEX2: A Complete Structure Solution, Refinement and Analysis Program. J. Appl. Crystallogr. 2009, 42, 339–341. 10.1107/S0021889808042726.

[ref31] Fugel M.; Jayatilaka D.; Hupf E.; Overgaard J.; Hathwar V. R.; Macchi P.; Turner M. J.; Howard J. A. K.; Dolomanov O. V.; Puschmann H.; Iversen B. B.; Bürgi H.-B.; Grabowsky S. Probing the Accuracy and Precision of Hirshfeld Atom Refinement with HARt Interfaced with Olex2. IUCrJ 2018, 5, 32–44. 10.1107/S2052252517015548. PMC5755575 29354269

[ref32] Wieduwilt E. K.; Macetti G.; Malaspina L. A.; Jayatilaka D.; Grabowsky S.; Genoni A. Post-Hartree-Fock Methods for Hirshfeld Atom Refinement: Are They Necessary? Investigation of a Strongly Hydrogen-Bonded Molecular Crystal. J. Mol. Struct. 2020, 1209, 127934 10.1016/j.molstruc.2020.127934.

[ref33] Chodkiewicz M. L.; Migacz S.; Rudnicki W.; Makal A.; Kalinowski J. A.; Moriarty N. W.; Grosse-Kunstleve R. W.; Afonine P. V.; Adams P. D.; Dominiak P. M. DiSCaMB: A Software Library for Aspherical Atom Model X-Ray Scattering Factor Calculations with CPUs and GPUs. J. Appl. Crystallogr. 2018, 51, 193–199. 10.1107/S1600576717015825. 29507550 PMC5822993

[ref34] Neese F. The ORCA Program System. WIREs Comput. Mol. Sci. 2012, 2, 73–78. 10.1002/wcms.81.

[ref35] Neese F. Software Update: The ORCA Program System, Version 4.0. Wiley Interdiscip. Rev. Comput. Mol. Sci. 2018, 8, e1327 10.1002/wcms.1327.

[ref36] Frisch M. J.; Trucks G. W.; Schlegel H. B.; S G. E.; Robb M. A.; Cheeseman J. R.; Scalmani G.; Barone V.; Petersson G. A.; Nakatsuji H.; Li X.; Caricato M.; Marenich A. V.; Bloino J.; Janesko B. G.; Gomperts R.; Mennucci B.; Hratchian H. P.; Ortiz J. V.; Izmaylov A. F.; Sonnenberg J. L.; Williams-Young D.; Ding F.; Lipparini F.; Egidi F.; Goings J.; Peng B.; Petrone A.; Henderson T.; Ranasinghe D.; Zakrzewski V. G.; Gao J.; Rega N.; Zheng G.; Liang W.; Hada M.; Ehara M.; Toyota K.; Fukuda R.; Hasegawa J.; Ishida M.; Nakajima T.; Honda Y.; Kitao O.; Nakai H.; Vreven T.; Throssell K.; Montgomery J. A. Jr.; Peralta J. E.; Ogliaro F.; Bearpark M. J.; Heyd J. J.; Brothers E. N.; Kudin K. N.; Staroverov V. N.; Keith T. A.; Kobayashi R.; Normand J.; Raghavachari K.; Rendell A. P.; Burant J. C.; Iyengar S. S.; Tomasi J.; Cossi M.; Millam J. M.; Klene M.; Adamo C.; Cammi R.; Ochterski J. W.; Martin R. L.; Morokuma K.; Farkas O.; Foresman J. B.; Fox D. J. Gaussian 16, Revision C.01.; Gaussian Inc. https://gaussian.com/citation/ (accessed 2022-03-09).

[ref37] Chodkiewicz M. L.; Woińska M.; Woźniak K. Hirshfeld Atom like Refinement with Alternative Electron Density Partitions. IUCrJ 2020, 7, 1199–1215. 10.1107/S2052252520013603. PMC7642787 33209330

[ref38] Reiher M.; Wolf A. Exact Decoupling of the Dirac Hamiltonian. II. The Generalized Douglas–Kroll–Hess Transformation up to Arbitrary Order. J. Chem. Phys. 2004, 121, 10945–10956. 10.1063/1.1818681. 15634044

[ref39] Reiher M.; Wolf A. Exact Decoupling of the Dirac Hamiltonian. I. General Theory. J. Chem. Phys. 2004, 121, 2037–2047. 10.1063/1.1768160. 15260757

[ref40] Wolf A.; Reiher M.; Hess B. A. The Generalized Douglas–Kroll Transformation. J. Chem. Phys. 2002, 117, 9215–9226. 10.1063/1.1515314. 15267790

[ref41] Reiher M. Douglas–Kroll–Hess Theory: A Relativistic Electrons-Only Theory for Chemistry. Theor. Chem. Acc. 2006, 116, 241–252. 10.1007/s00214-005-0003-2.

[ref42] Bučinský L.; Jayatilaka D.; Grabowsky S. Importance of Relativistic Effects and Electron Correlation in Structure Factors and Electron Density of Diphenyl Mercury and Triphenyl Bismuth. J. Phys. Chem. A 2016, 120, 6650–6669. 10.1021/acs.jpca.6b05769. 27434184

[ref43] Bućinský L.; Jayatilaka D.; Grabowsky S. Relativistic Quantum Crystallography of Diphenyl- and Dicyanomercury. Theoretical Structure Factors and Hirshfeld Atom Refinement. Acta Crystallogr., Sect. A: Found. Adv. 2019, 75, 705–717. 10.1107/S2053273319008027. 31475915

[ref44] Malaspina L. A.; Wieduwilt E. K.; Bergmann J.; Kleemiss F.; Meyer B.; Ruiz-López M. F.; Pal R.; Hupf E.; Beckmann J.; Piltz R. O.; Edwards A. J.; Grabowsky S.; Genoni A. Fast and Accurate Quantum Crystallography: From Small to Large, from Light to Heavy. J. Phys. Chem. Lett. 2019, 10, 6973–6982. 10.1021/acs.jpclett.9b02646. 31633355

[ref45] Pawlędzio S.; Malinska M.; Woińska M.; Wojciechowski J.; Andrade Malaspina L.; Kleemiss F.; Grabowsky S.; Woźniak K. Relativistic Hirshfeld Atom Refinement of an Organo-Gold(I) Compound. IUCrJ 2021, 8, 608–620. 10.1107/S2052252521004541. PMC8256711 34258009

[ref46] Ho N. N.; Bau R.; Mason S. A. Neutron Diffraction Study of the Highly Distorted Octahedral Complex FeH2(CO)2[P(OPh)3]2. J. Organomet. Chem. 2003, 676, 85–88. 10.1016/S0022-328X(03)00312-7.

[ref47] Arion V.; Brunet J.-J.; Neibecker D. Crystal Structure, Mössbauer Spectra, and Thermal Behavior of H_2_Fe(CO)_2_[P(OPh)_3_]_2_. Inorg. Chem. 2001, 40, 2628–2630. 10.1021/ic0006899. 11350245

[ref48] Grellier M.; Vendier L.; Chaudret B.; Albinati A.; Rizzato S.; Mason S.; Sabo-Etienne S. Synthesis, Neutron Structure, and Reactivity of the Bis(Dihydrogen) Complex RuH_2_(η^2^-H_2_)_2_(PCyp_3_)_2_ Stabilized by Two Tricyclopentylphosphines. J. Am. Chem. Soc. 2005, 127, 17592–17593. 10.1021/ja055126g. 16351074

[ref49] Smart K. A.; Grellier M.; Vendier L.; Mason S. A.; Capelli S. C.; Albinati A.; Sabo-Etienne S. Step-by-Step Introduction of Silazane Moieties at Ruthenium: Different Extents of Ru–H–Si Bond Activation. Inorg. Chem. 2013, 52, 2654–2661. 10.1021/ic302682f. 23421738

[ref50] Smart K. A.; Grellier M.; Coppel Y.; Vendier L.; Mason S. A.; Capelli S. C.; Albinati A.; Montiel-Palma V.; Muñoz-Hernández M. A.; Sabo-Etienne S. Nature of Si–H Interactions in a Series of Ruthenium Silazane Complexes Using Multinuclear Solid-State NMR and Neutron Diffraction. Inorg. Chem. 2014, 53, 1156–1165. 10.1021/ic4027199. 24392827

[ref51] Lam W. H.; Shimada S.; Batsanov A. S.; Lin Z.; Marder T. B.; Cowan J. A.; Howard J. A. K.; Mason S. A.; McIntyre G. J. Accurate Molecular Structures of 16-Electron Rhodium Hydrido Boryl Complexes: Low-Temperature Single-Crystal X-Ray and Neutron Diffraction and Computational Studies of [(PR3)2RhHCl(Boryl)] (Boryl = Bpin, Bcat). Organometallics 2003, 22, 4557–4568. 10.1021/om030434d.

[ref52] Grellier M.; Ayed T.; Barthelat J.-C.; Albinati A.; Mason S.; Vendier L.; Coppel Y.; Sabo-Etienne S. Versatile Coordination of 2-Pyridinetetramethyldisilazane at Ruthenium: Ru(II) vs Ru(IV) As Evidenced by NMR, X-Ray, Neutron, and DFT Studies. J. Am. Chem. Soc. 2009, 131, 7633–7640. 10.1021/ja901140v. 19435352

[ref53] Bakhmutov V. I.; Howard J. A. K.; Keen D. A.; Kuzmina L. G.; Leech M. A.; Nikonov G. I.; Vorontsov E. V.; Wilson C. C. Combined Single Crystal Neutron Diffraction and Solution NMR Relaxation Studies of Mono- and Bis(Silyl) Substituted Niobocene Hydrides with Nonclassical Interligand Interactions. J. Chem. Soc., Dalton Trans. 2000, 10, 1631–1635. 10.1039/B000089M.

[ref54] Nikonov G. I.; Kuzmina L. G.; Lemenovskii D. A.; Kotov V. V. Interligand Hypervalent Interaction in the Bis(Silyl) Hydride Derivatives of Niobocene. J. Am. Chem. Soc. 1995, 117, 10133–10134. 10.1021/ja00145a033.

[ref55] Cammarota R. C.; Xie J.; Burgess S. A.; Vollmer M. V.; Vogiatzis K. D.; Ye J.; Linehan J. C.; Appel A. M.; Hoffmann C.; Wang X.; Young V. G.; Lu C. C. Thermodynamic and Kinetic Studies of H_2_ and N_2_ Binding to Bimetallic Nickel-Group 13 Complexes and Neutron Structure of a Ni(η^2^-H_2_) Adduct. Chem. Sci. 2019, 10, 7029–7042. 10.1039/C9SC02018G. 31588270 PMC6676469

[ref56] Schwamm R. J.; Edwards A. J.; Fitchett C. M.; Coles M. P. A Study of Di(Amino)Stibines with Terminal Sb(III) Hydrogen-Ligands by X-Ray- and Neutron-Diffraction. Dalton Trans. 2019, 48, 2953–2958. 10.1039/C8DT05113E. 30741279

[ref57] Webster C. E.; Gross C. L.; Young D. M.; Girolami G. S.; Schultz A. J.; Hall M. B.; Eckert J. Electronic and Steric Effects on Molecular Dihydrogen Activation in [Cp*OsH4(L)]+ (L = PPh3, AsPh3, and PCy3). J. Am. Chem. Soc. 2005, 127, 15091–15101. 10.1021/ja052336k. 16248648

[ref58] Gross C. L.; Girolami G. S. Synthesis and NMR Studies of [(C_5_Me_5_)Os(L)H_2_(H_2_)^+^] Complexes. Evidence of the Adoption of Different Structures by a Dihydrogen Complex in Solution and the Solid State. Organometallics 2007, 26, 1658–1664. 10.1021/om061017e.

[ref59] Canal Neto A.; Jorge F. E. All-Electron Double Zeta Basis Sets for the Most Fifth-Row Atoms: Application in DFT Spectroscopic Constant Calculations. Chem. Phys. Lett. 2013, 582, 158–162. 10.1016/j.cplett.2013.07.045.

[ref60] Canal Neto A.; Muniz E. P.; Centoducatte R.; Jorge F. E. Gaussian Basis Sets for Correlated Wave Functions. Hydrogen, Helium, First- and Second-Row Atoms. J. Mol. Struct.: THEOCHEM 2005, 718, 219–224. 10.1016/j.theochem.2004.11.037.

[ref61] Becke A. D. Density-Functional Exchange-Energy Approximation with Correct Asymptotic Behavior. Phys. Rev. A 1988, 38, 3098–3100. 10.1103/PhysRevA.38.3098. 9900728

[ref62] Lee C.; Yang W.; Parr R. G. Development of the Colle-Salvetti Correlation-Energy Formula into a Functional of the Electron Density. Phys. Rev. B 1988, 37, 785–789. 10.1103/PhysRevB.37.785. 9944570

[ref63] Perdew J. P.; Ernzerhof M.; Burke K. Rationale for Mixing Exact Exchange with Density Functional Approximations. J. Chem. Phys. 1996, 105, 9982–9985. 10.1063/1.472933.

[ref64] Perdew J. P.; Burke K.; Ernzerhof M. Generalized Gradient Approximation Made Simple. Phys. Rev. Lett. 1996, 77, 3865–3868. 10.1103/PhysRevLett.77.3865. 10062328

[ref65] Zhao Y.; Truhlar D. G. Density Functional for Spectroscopy: No Long-Range Self-Interaction Error, Good Performance for Rydberg and Charge-Transfer States, and Better Performance on Average than B3LYP for Ground States. J. Phys. Chem. A 2006, 110, 13126–13130. 10.1021/jp066479k. 17149824

[ref66] Zhao Y.; Truhlar D. G. The M06 Suite of Density Functionals for Main Group Thermochemistry, Thermochemical Kinetics, Noncovalent Interactions, Excited States, and Transition Elements: Two New Functionals and Systematic Testing of Four M06-Class Functionals and 12 Other Functionals. Theor. Chem. Acc. 2008, 120, 215–241. 10.1007/s00214-007-0310-x.

[ref67] Dunning T. H. Jr. Gaussian Basis Sets for Use in Correlated Molecular Calculations. I. The Atoms Boron through Neon and Hydrogen. J. Chem. Phys. 1989, 90, 1007–1023. 10.1063/1.456153.

[ref68] Balabanov N. B.; Peterson K. A. Basis Set Limit Electronic Excitation Energies, Ionization Potentials, and Electron Affinities for the 3d Transition Metal Atoms: Coupled Cluster and Multireference Methods. J. Chem. Phys. 2006, 125, 074110 10.1063/1.2335444. 16942325

[ref69] Jorge F. E.; Canal Neto A.; Camiletti G. G.; Machado S. F. Contracted Gaussian Basis Sets for Douglas–Kroll–Hess Calculations: Estimating Scalar Relativistic Effects of Some Atomic and Molecular Properties. J. Chem. Phys. 2009, 130, 064108 10.1063/1.3072360. 19222268

[ref70] Hill J. G.; Peterson K. A. Gaussian Basis Sets for Use in Correlated Molecular Calculations. XI. Pseudopotential-Based and All-Electron Relativistic Basis Sets for Alkali Metal (K–Fr) and Alkaline Earth (Ca–Ra) Elements. J. Chem. Phys. 2017, 147, 244106 10.1063/1.5010587. 29289120

[ref71] Pritchard B. P.; Altarawy D.; Didier B.; Gibson T. D.; Windus T. L. New Basis Set Exchange: An Open, Up-to-Date Resource for the Molecular Sciences Community. J. Chem. Inf. Model. 2019, 59, 4814–4820. 10.1021/acs.jcim.9b00725. 31600445

[ref72] Verstraelen T.; Tecmer P.; Heidar-Zadeh F.; González-Espinoza C. E.; Chan M.; Kim T. D.; Boguslawski K.; Fias S.; Vandenbrande S.; Berrocal D. Horton 2.0. 1. *Available at theochem. github. com/horton/. Accessed August* 2016, 28, 2017.

[ref73] Welch B. L. The Generalisation of Student’s Problems When Several Different Population Variances Are Involved. Biometrika 1947, 34, 28–35. 10.1093/biomet/34.1-2.28. 20287819

[ref74] Woińska M.; Chodkiewicz M. L.; Woźniak K. Towards Accurate and Precise Positions of Hydrogen Atoms Bonded to Heavy Metal Atoms. Chem. Commun. 2021, 57, 3652–3655. 10.1039/D0CC07661A. 33870351

[ref75] Lu T.; Chen F. Multiwfn: A Multifunctional Wavefunction Analyzer. J. Comput. Chem. 2012, 33, 580–592. 10.1002/jcc.22885. 22162017

