# Supplementary material for: Hirshfeld Atom Refinement of Metal–Organic Complexes: Treatment of Hydrogen Atoms Bonded to Transition Metals
Source: J Phys Chem A. 2023 Mar 22;127(13):3020–35. doi: 10.1021/acs.jpca.2c06998 (PMC10084459; doi:10.1021/acs.jpca.2c06998)
Supplement: Supplementary file 1 — jp2c06998_si_001.pdf [file jp2c06998_si_001.pdf]

Supporting Information

Hirshfeld Atom Refinement of Metal Organic Complexes – Treatment of Hydrogen Atoms Bonded to Transition Metals

Magdalena Woińska, Sylwia Pawlędzio, Michał L. Chodkiewicz, Krzysztof Woźniak

Biological and Chemical Research Centre, Chemistry Department, University of Warsaw, Żwirki i Wigury 101, 02-089 Warszawa, Poland.

Correspondence to: magdalena.woinska@uw.edu.pl, kwozniak@chem.uw.edu.pl

KEYWORDS: HAR, metal organic complexes, transition metals, hydrides, Quantum Crystallography, refinement, DFT

Table S1 Experimental and computational details of the neutron, X-ray IAM and X-ray HAR crystal structures of QOSZON. HAR was performed with a cluster of charges and dipoles modeling crystal environment (DiSCaMB) and without a cluster (NoSpherA2). DFT calculations for HAR were performed with various functionals in the non-relativistic version (B3LYP, PBE and M06-2X) and with relativistic correction (B3LYP-DKH2, PBE-DKH2 and M06-2X-DKH2). Basis sets: [a] cc-pVDZ; [b] cc-pVTZ; [c] cc-pVDZ-DK, [d] cc-pVTZ-DK. The IAM structures were re-refined with *Olex2.refine* based on the original structures.

			**DiSCaMB-HAR**						**NoSpherA2-HAR**					
**QOSZON**	**neutron**	**IAM**	**B3LYP**	**PBE**	**M06-2X**	**B3LYP-DKH2**	**PBE-DKH2**	**M06-2X-DKH2**	**B3LYP**	**PBE**	**M06-2X**	**B3LYP-DKH2**	**PBE-DKH2**	**M06-2X-DKH2**
**REFCODE [literature reference]**	**QOSZON01**	**QOSZON**												
**Chemical formula**	$C_{38}H_{32}FeO_8P_2$													
**Space group**	P -1													
**Temperature (K)**	20(2)	293(2)												
**Wavelength [Å]**	1.315	0.71073												
**Theta range (deg)**	2.14-61.68	2.11-26.09												
**sin(θ)/ λ Å^{-1}**	0.67	0.62												
**Completeness**	0.761	0.919												
**R_{int}**	0.0217	0.0344												
**Year of publication**	2003	2001												
**Parameters**	733	570	720 [a]<br>720 [b]											
**Goodness of fit**	1.41	1.00	1.22 [a]<br>0.97 [b]	1.21 [a]<br>1.18 [b]	1.22 [a]<br>1.18 [b]	1.22 [c]<br>1.18 [d]	1.21 [c]<br>1.18 [d]	1.21 [c]<br>1.18 [d]	0.96 [a]<br>0.97 [b]	0.96 [a]<br>0.97 [b]	0.96 [a]<br>0.97 [b]	0.97 [c]<br>0.97 [d]	0.97 [c]<br>0.97 [d]	0.96 [c]<br>0.97 [d]
**R[%]**<br><br>**(reflections)**	2.64<br><br>(6301)	2.68<br><br>(5084)	1.97 [a]<br>1.87 [b]<br>(5084)	1.97 [a]<br>1.91 [b]<br>(5084)	1.97 [a]<br>1.91 [b]<br>(5084)	1.97 [c]<br>1.91 [d]<br>(5084)	1.96 [c]<br>1.90 [d]<br>(5084)	1.97 [c]<br>1.91 [d]<br>(5084)	1.92 [a]<br>1.87 [b]<br>(5084)	1.92 [a]<br>1.86 [b]<br>(5084)	1.92 [a]<br>1.87 [b]<br>(5084)	1.92 [c]<br>1.86 [d]<br>(5084)	1.91 [c]<br>1.86 [d]<br>(5084)	1.92 [c]<br>1.86 [d]<br>(5084)
**wR2[%]**<br><br>**(reflections)**	5.44<br><br>(6402)	6.92<br><br>(6202)	3.32 [a]<br>3.89 [b]<br>(6202)	3.20 [a]<br>3.21 [b]<br>(6202)	3.32 [a]<br>3.23 [b]<br>(6202)	3.32 [c]<br>3.22 [d]<br>(6202)	3.30 [c]<br>3.21 [d]<br>(6202)	3.31 [c]<br>3.22 [d]<br>(6202)	4.11 [a]<br>3.90 [b]<br>(6202)	4.11 [a]<br>3.85 [b]<br>(6202)	4.12 [a]<br>3.88 [b]<br>(6202)	4.06 [c]<br>3.89 [d]<br>(6202)	4.09 [c]<br>3.84 [d]<br>(6202)	4.09 [c]<br>3.87 [d]<br>(6202)
**$\Delta\rho_{min/max}$ (eÅ^{-3})**	-0.36/0.37	-0.34/0.34	-0.24/0.25 [a]<br>-0.24/0.23 [b]	-0.24/0.25[a]<br>-0.23/0.23[b]	-0.26/0.24 [a]<br>-0.25/0.22 [b]	-0.24/0.25 [c]<br>-0.23/0.23 [d]	-0.24/0.24 [c]<br>-0.23/0.23 [d]	-0.25/0.23 [c]<br>-0.24/0.22[d]	-0.250.23 [a]<br>-0.24/0.22 [b]	-0.24/0.24 [a]<br>-0.22/0.24 [b]	-0.26/0.22 [a]<br>-0.26/0.22 [b]	-0.24/0.23 [c]<br>-0.24/0.22 [d]	-0.23/0.25 [c]<br>-0.22/0.23 [d]	-0.26/0.22 [c]<br>-0.26/0.21 [d]
**Refined H positions**	all	all	all	all	all	all	all	all	all	all	all	all	all	all
**H thermal motions**	aniso	iso	anis + iso (H1, H2)											

Table S2 Experimental and computational details of the neutron, X-ray IAM and X-ray HAR crystal structures of NEBNEO. HAR was performed with a cluster of charges and dipoles modeling crystal environment (DiSCaMB) and without a cluster (NoSpherA2). DFT calculations for HAR were performed with various functionals in the non-relativistic version (B3LYP, PBE and M06-2X) and with relativistic correction (B3LYP-DKH2, PBE-DKH2 and M06-2X-DKH2). Basis sets: cc-pVTZ-DK. The IAM structures were re-refined with *Olex2.refine* based on the original structures.

			DiSCaMB-HAR						NoSpherA2-HAR					
**NEBNEO**	**neutron**	**IAM**	**B3LYP**	**PBE**	**M06-2X**	**B3LYP-DKH2**	**PBE-DKH2**	**M06-2X-DKH2**	**B3LYP**	**PBE**	**M06-2X**	**B3LYP-DKH2**	**PBE-DKH2**	**M06-2X-DKH2**
**REFCODE [literature reference]**	**NEBNEO**	**NEBNEO01**												
**Chemical formula**	$C_{30}H_{60}P_2Ru$													
**Space group**	$P\ 2_1/n$													
**Temperature (K)**	20(1)	100												
**Wavelength [Å]**	1.31600	0.71073												
**Theta range (deg)**	2.77- 63.02	1.65-26.05												
**sin(θ)/ λ Å^{-1}**	0.68	0.75												
**Completeness**	NA	0.9348												
**R_{int}**	NA	NA												
**Year of publication**	2005	2005												
**Parameters**	838	538	538											
**Goodness of fit**	1.07	1.28	1.09	1.09	1.09	1.08	1.08	1.08	0.92	0.92	0.92	0.92	0.92	0.92
**R[%] (reflections)**	5.21 (4979)	3.75 (6749)	3.47 (6749)	3.45 (6749)	3.45 (6749)	3.44 (6749)	3.43 (6749)	3.43 (6749)	3.37 (6749)	3.36 (6749)	3.36 (6749)	3.37 (6749)	3.37 (6749)	3.36 (6749)
**wR2[%] (reflections)**	14.40 (6498)	4.76 (9779)	4.06 (9779)	4.04 (9779)	4.04 (9779)	4.03 (9779)	4.01 (9779)	4.02 (9779)	5.98 (9779)	5.98 (9779)	5.93 (9779)	5.97 (9779)	5.97 (9779)	5.93 (9779)
**$\Delta\rho_{min/max}$ (eÅ^{-3})**	-0.09/0.12	-0.90/0.94	-1.01/0.87	-0.99/0.87	-1.01/0.86	-0.900.85	-0.87/0.83	-0.87/0.84	-0.82/0.86	-0.81/0.86	-0.80/0.89	-0.83/0.85	-0.81/0.84	-0.82/0.85
**Refined H positions**	all	H100, H101, H102, H103, H104, H105	all	all	all	all	all	all	all	all	all	all	all	all
**H thermal motions**	anis	iso	iso											

Table S3 Experimental and computational details of the neutron, X-ray IAM and X-ray HAR crystal structures of MIGKIY. HAR was performed with a cluster of charges and dipoles modeling crystal environment (DiSCaMB) and without a cluster (NoSpherA2). DFT calculations for HAR were performed with various functionals in the non-relativistic version (B3LYP, PBE and M06-2X) and with relativistic correction (B3LYP-DKH2, PBE-DKH2 and M06-2X-DKH2). Basis sets: cc-pVTZ-DK. The IAM structures were re-refined with *Olex2.refine* based on the original structures.

			**DiSCaMB-HAR**						**NoSpherA2-HAR**					
**MIGKIY**	**neutron**	**IAM**	**B3LYP**	**PBE**	**M06-2X**	**B3LYP-DKH2**	**PBE-DKH2**	**M06-2X-DKH2**	**B3LYP**	**PBE**	**M06-2X**	**B3LYP-DKH2**	**PBE-DKH2**	**M06-2X-DKH2**
**REFCODE [literature reference]**	**MIGKIY01**	**MIGKIY**												
**Chemical formula**	$C_{32}H_{12}BF_{24}$, $C_{24}H_{41}N_6RuSi_3$													
**Space group**	P n													
**Temperature (K)**	20(1)	100(2)												
**Wavelength [Å]**	1.1708	0.71073												
**Theta range (deg)**	4.82-61.29	2.94-29.40												
**sin(θ)/ λ Å^{-1}**	0.75	0.69												
**Completeness**	0.88	0.89												
**R_{int}**	0.1253	0.0429												
**Year of publication**	2013	2013												
**Parameters**	1297	828	1032											
**Goodness of fit**	1.06	1.06	1.07	1.07	1.07	1.07	1.07	1.07	1.07	1.07	1.07	1.07	1.07	1.07
**R[%] (reflections)**	6.17 (7647)	3.82 (13569)	3.56 (13569)	3.54 (13569)	3.55 (13569)	3.57 (13569)	3.56 (13569)	3.56 (13569)	3.57 (13569)	3.57 (13569)	3.57 (13569)	3.57 (13569)	3.56 (13569)	3.56 (13569)
**wR2[%] (reflections)**	13.10 (9494)	9.24 (15166)	8.39 (15166)	8.27 (15166)	8.34 (15166)	8.43 (15166)	8.35 (15166)	8.41 (15166)	8.45 (15166)	8.41 (15166)	8.40 (15166)	8.43 (15166)	8.39 (15166)	8.38 (15166)
**$\Delta\rho_{min/max}$ (eÅ^{-3})**	-1.30/1.30	-0.47/0.93	-0.47/0.95	-0.48/ 0.94	-0.48/ 0.94	-0.47/ 0.95	-0.48/ 0.95	-0.48/ 0.95	-0.47/0.94	-0.47/0.94	-0.48/ 0.94	-0.47/0.94	-0.47/0.94	-0.48/ 0.94
**Refined H positions**	all	Ru-H, Si-H	all	all	all	all	all	all	all	all	all	all	all	all
**H thermal motions**	aniso	iso	iso											

Table S4 Experimental and computational details of the neutron, X-ray IAM and X-ray HAR crystal structures of NOBBOX. HAR was performed with a cluster of charges and dipoles modeling crystal environment (DiSCaMB) and without a cluster (NoSpherA2). DFT calculations for HAR were performed with various functionals in the non-relativistic version (B3LYP, PBE and M06-2X) and with relativistic correction (B3LYP-DKH2, PBE-DKH2 and M06-2X-DKH2). Basis sets: cc-pVTZ-DK. The IAM structures were re-refined with *Olex2.refine* based on the original structures.

			**DiSCaMB-HAR**						**NoSpherA2-HAR**					
**NOBBOX**	**neutron**	**IAM**	**B3LYP**	**PBE**	**M06-2X**	**B3LYP-DKH2**	**PBE-DKH2**	**M06-2X-DKH2**	**B3LYP**	**PBE**	**M06-2X**	**B3LYP-DKH2**	**PBE-DKH2**	**M06-2X-DKH2**
**REFCODE [literature reference]**	**NOBBOX01**	**NOBBOX**												
**Chemical formula**	$C_{27}H_{55}N_9RuSi_3$													
**Space group**	P 2_1/c													
**Temperature (K)**	20(2)	100(2)												
**Wavelength [Å]**	1.17	0.71073												
**Theta range (deg)**	4.94-60.30	1.54-36.39												
**sin(θ)/ λ $Å^{-1}$**	0.74	0.83												
**Completeness**	0.884	1												
**R_{int}**	0.0517	0.0222												
**Year of publication**	2014	2014												
**Parameters**	856	365	856						856	694	856	694	694	856
**Goodness of fit**	1.12	1.01	0.97	0.96	0.96	0.97	0.97	0.97	0.97	1.01	0.97	1.01	1.02	0.97
**R[%] (reflections)**	4.39 (9100)	2.61 (11599)	2.08 (11599)	2.06 (11599)	2.07 (11599)	2.11 (11599)	2.10 (11599)	2.11 (11599)	2.12 (11599)	2.37 (11599)	2.12 (11599)	2.35 (11599)	2.36 (11599)	2.11 (11599)
**wR2[%] (reflections)**	8.39 (15166)	5.10 (14045)	3.40 (14045)	3.36 (14045)	3.38 (14045)	3.55 (14045)	3.50 (14045)	3.55 (14045)	3.66 (14045)	3.58 (14045)	3.60 (14045)	4.94 (14045)	5.02 (14045)	3.55 (14045)
**$\Delta\rho_{min/max}$ ($eÅ^{-3}$)**	-1.08/1.25	-0.47/0.67	-0.41/0.48	-0.41/0.48	-0.41/0.46	-0.41/0.58	-0.41/0.57	-0.41/0.56	-0.48/0.50	-0.45/0.65	-0.47/0.49	-0.45/0.60	-0.45/0.63	-0.47/0.46
**Refined H positions**	all	Ru-H	all	all	all	all	all	all	all	all	all	all	all	all
**H thermal motions**	aniso	iso	aniso											

Table S5 Experimental and computational details of the neutron, X-ray IAM and X-ray HAR crystal structures of SITKUB. HAR was performed with a cluster of charges and dipoles modeling crystal environment (DiSCaMB) and without a cluster (NoSpherA2). DFT calculations for HAR were performed with various functionals in the non-relativistic version (B3LYP, PBE and M06-2X) and with relativistic correction (B3LYP-DKH2, PBE-DKH2 and M06-2X-DKH2). Basis sets: [a] cc-pVTZ-DK; [b] jorge-DZP; [c] jorge-TZP; [d] jorge-DZP-DKH, [e] jorge-TZP-DKH. The IAM structures were re-refined with *Olex2.refine* based on the original structures.

			**DiSCaMB-HAR**						**NoSpherA2-HAR**					
**SITKUB**	**neutron**	**IAM**	**B3LYP**	**PBE**	**M06-2X**	**B3LYP-DKH2**	**PBE-DKH2**	**M06-2X-DKH2**	**B3LYP**	**PBE**	**M06-2X**	**B3LYP-DKH2**	**PBE-DKH2**	**M06-2X-DKH2**
**REFCODE [literature reference]**	**SITKUB02**	**SITKUB01**												
**Chemical formula**	$C_{24}H_{47}BClO_2P_2Rh$													
**Space group**	$P\ 2_1/c$													
**Temperature (K)**	20(2)	120.15												
**Wavelength [Å]**	1.5453	0.71073												
**Theta range (deg)**	3.9-66.94	1.78-29.06												
**sin(θ)/ λ Å^{-1}**	0.60	0.68												
**Completeness**	0.474	0.998												
**R_{int}**	0.0574	0.0293												
**Year of publication**	2003	2003												
**Parameters**	473	354	703 [a]<br>678 [b]<br>703[c]	703 [a]<br>683 [b]<br>703 [c]	698 [a]<br>683 [b]<br>703 [c]	703 [a]<br>673 [d]<br>703 [e]	703 [a]<br>688 [d]<br>703 [e]	703 [a]<br>688 [d]<br>703 [e]	703 [a]<br>688 [b]<br>698 [c]	698 [a]<br>688 [b]<br>698 [c]	703 [a]<br>688 [b]<br>698 [c]	703 [a]<br>688 [d]<br>698 [e]	703 [a]<br>688 [d]<br>698 [e]	703 [a]<br>688 [d]<br>698 [e]
**Goodness of fit**	1.07	1.06	1.73 [a]<br>2.02[b]<br>1.74[c]	1.09 [a]<br>1.99 [b]<br>1.72 [c]	1.10 [a]<br>1.99 [b]<br>1.72 [c]	1.10 [a]<br>2.02 [d]<br>1.76 [e]	1.10 [a]<br>1.99 [d]<br>1.75 [e]	1.10 [a]<br>1.98 [d]<br>1.75 [e]	1.10 [a]<br>1.09 [b]<br>1.09 [c]	1.10 [a]<br>1.09 [b]<br>1.10 [c]	1.10 [a]<br>1.09 [b]<br>1.09 [c]	1.10 [a]<br>1.09 [d]<br>1.09 [e]	1.11 [a]<br>1.09 [d]<br>1.09 [e]	1.13 [a]<br>1.09 [d]<br>1.10 [e]
**R[%]**<br>**(reflections)**	6.49<br>(1908)	2.56<br>(7021)	2.15 [a]<br>2.02 [b]<br>2.16 [c]<br>(7021)	2.10 [a]<br>2.34 [b]<br>2.14 [c]<br>(7021)	2.11 [a]<br>2.34 [b]<br>2.14 [c]<br>(7021)	2.15 [a]<br>2.36 [d]<br>2.18 [e]<br>(7021)	2.14 [a]<br>2.34 [d]<br>2.17 [e]<br>(7021)	2.14 [a]<br>2.34 [d]<br>2.17 [e]<br>(7021)	2.18 [a]<br>2.34 [b]<br>2.16 [c]<br>(7021)	2.17 [a]<br>2.34 [b]<br>2.17 [c]<br>(7021)	2.19 [a]<br>2.33 [b]<br>2.15 [c]<br>(7021)	2.15 [a]<br>2.32 [d]<br>2.15 [e]<br>(7021)	2.16 [a]<br>2.31 [d]<br>2.15 [e]<br>(7021)	2.14 [a]<br>2.32 [d]<br>2.13 [e]<br>(7021)
**wR2[%]**<br>**(reflections)**	16.62<br>(2356)	5.77<br>(7624)	2.86 [a]<br>3.34 [b]<br>2.87 [c]<br>(7624)	4.08 [a]<br>3.29 [b]<br>2.85 [c]<br>(7624)	4.09 [a]<br>3.29 [b]<br>2.85 [c]<br>(7624)	4.22 [a]<br>3.34 [d]<br>2.92 [e]<br>(7624)	4.20 [a]<br>3.29 [d]<br>2.89 [e]<br>(7624)	4.22 [a]<br>3.28 [d]<br>2.89 [e]<br>(7624)	4.38 [a]<br>4.85 [b]<br>4.30 [c]<br>(7624)	4.30 [a]<br>4.81 [b]<br>4.33 [c]<br>(7624)	4.38 [a]<br>4.76 [b]<br>4.24 [c]<br>(7624)	4.29 [a]<br>4.76 [d]<br>4.25 [e]<br>(7624)	4.26 [a]<br>4.74 [d]<br>4.28 [e]<br>(7624)	4.23 [a]<br>4.76 [d]<br>4.20 [e]<br>(7624)
**$\Delta\rho_{min/max}$ (eÅ^{-3})**	-0.73/0.56	-0.79/0.47	-0.76/0.42 [a]<br>-0.76/0.42 [b]<br>-0.75/0.43 [c]	-0.79/0.40 [a]<br>-0.76/0.41 [b]<br>-0.76/0.42 [c]	-0.78/0.40 [a]<br>-0.76/0.41 [b]<br>-0.76/0.43 [c]	-0.77/0.42 [a]<br>-0.76/0.43 [d]<br>-0.75/0.45 [e]	-0.77/0.42 [a]<br>-0.76/0.41 [d]<br>-0.75/0.44 [e]	-0.78/0.44 [a]<br>-0.76/0.41 [d]<br>-0.76/0.46 [e]	-0.80/0.54 [a]<br>-0.73/0.46 [b]<br>-0.75/0.42 [c]	-0.77/0.44 [a]<br>-0.73/0.45 [b]<br>-0.76/0.40 [c]	-0.81/0.63 [a]<br>-0.73/0.46 [b]<br>-0.75/0.42 [c]	-0.77/0.42 [a]<br>-0.73/0.46[d]<br>-0.74/0.42 [e]	-0.77/0.43 [a]<br>-0.73/0.45 [d]<br>-0.76/0.41 [e]	-0.78/0.41 [a]<br>-0.73/0.46 [d]<br>-0.75/0.44 [e]
**Refined H positions**	all	Rh-H	all	all	all	all	all	all	all	all	all	all	all	all
**H thermal motions**	iso	iso	aniso [a]<br>aniso + iso (H, H5, H9A, H10, H13) [b]<br>aniso [c]	aniso [a]<br>aniso + iso (H, H5, H9A, H13) [b]<br>aniso [c]	aniso [a]<br>aniso + iso (H, H5, H9A, H13) [b]<br>aniso [c]	aniso [a]<br>aniso + iso (H5, H9A, H10, H13, H22) [d]<br>aniso [e]	aniso [a]<br>aniso + iso (H5, H9A, H10, H13) [d]<br>aniso [e]	aniso [a]<br>aniso + iso (H5, H9A, H13) [d]<br>aniso [e]	aniso [a]<br>aniso + iso (H, H9A, H17C) [b]<br>aniso + iso (H17C) [c]	aniso + iso (H17C) [a]<br>aniso + iso (H, H9A, H17C) [b]<br>aniso + iso (H17C) [c]	aniso [a]<br>aniso + iso (H, H9A, H17C) [b]<br>aniso + iso (H17C) [c]	aniso [a]<br>aniso + iso (H, H9A, H17C) [d]<br>aniso + iso (H17C) [e]	aniso [a]<br>aniso + iso (H, H9A, H17C) [d]<br>aniso + iso (H17C) [e]	aniso [a]<br>aniso + iso (H, H9A, H17C) [d]<br>aniso + iso (H17C) [e]

Table S6 This is a supplementary table heading (style name: IUCr sup table caption; this style applies table numbering). Please use the **IUCr tables** (toolbar button) to create experimental and geometry tables when reporting crystal structure data. Experimental and computational details of the neutron, X-ray IAM and X-ray HAR crystal structures of UJABOX. HAR was performed with a cluster of charges and dipoles modeling crystal environment (DiSCaMB) and without a cluster (NoSpherA2). DFT calculations for HAR were performed with various functionals in the non-relativistic version (B3LYP, PBE and M06-2X) and with relativistic correction (B3LYP-DKH2, PBE-DKH2 and M06-2X-DKH2). Basis sets: cc-pVTZ-DK. The IAM structures were re-refined with *Olex2.refine* based on the original structures.

			DiSCaMB-HAR						NoSpherA2-HAR					
UJABOX	**neutron**	**IAM**	**B3LYP**	**PBE**	**M06-2X**	**B3LYP-DKH2**	**PBE-DKH2**	**M06-2X-DKH2**	**B3LYP**	**PBE**	**M06-2X**	**B3LYP-DKH2**	**PBE-DKH2**	**M06-2X-DKH2**
**REFCODE [literature reference]**	**UJABOX01**	**UJABOX**												
**Chemical formula**	$C_{27}H_{52}N_6RuSi_6$													
**Space group**	R -3													
**Temperature (K)**	20(2)	110												
**Wavelength [Å]**	1.16954(2)	0.71073												
**Theta range (deg)**	2.74-61.64	3.31-32.12												
**sin(θ)/ λ $Å^{-1}$**	0.75	0.75												
**Completeness**	NA	0.9143												
**R_{int}**	0.097	0.051												
**Year of publication**	2009	2009												
**Parameters**	278	191	191											
**Goodness of fit**	1.12	1.11	1.12	1.12	1.12	1.12	1.12	1.12	1.12	1.12	1.12	1.12	1.12	1.12
**R[%] (reflections)**	13.06 (3216)	3.87 (1428)	3.49 (1428)	3.48 (1428)	3.48 (1428)	3.49 (1428)	3.49 (1428)	3.49 (1428)	3.50 (1428)	3.49 (1428)	3.50 (1428)	3.49 (1428)	3.49 (1428)	3.49 (1428)
**wR2[%] (reflections)**	14.83 (4285)	7.01 (1619)	5.91 (1619)	5.88 (1619)	5.89 (1619)	5.93 (1619)	5.90 (1619)	5.91 (1619)	5.94 (1619)	5.94 (1619)	5.92 (1619)	5.92 (1619)	5.92 (1619)	5.90 (1619)
**$\Delta\rho_{min/max}$ ($eÅ^{-3}$)**	-1.22/1.36	-0.43/0.60	-0.33/0.57	-0.33/0.60	-0.33/0.59	-0.33/0.58	-0.33/0.62	-0.33/0.59	-0.36/0.66	-0.39/0.65	-0.35/0.68	-0.36/0.64	-0.39/0.67	-0.35/0.66
**Refined H positions**	all	all	all	all	all	all	all	all	all	all	all	all	all	all
**H thermal motions**		iso	iso											

Table S7 Experimental and computational details of the neutron, X-ray IAM and X-ray HAR crystal structures of ZEYVAA. HAR was performed with a cluster of charges and dipoles modeling crystal environment (DiSCaMB) and without a cluster (NoSpherA2). DFT calculations for HAR were performed with various functionals in the non-relativistic version (B3LYP, PBE and M06-2X) and with relativistic correction (B3LYP-DKH2, PBE-DKH2 and M06-2X-DKH2). Basis sets: cc-pVTZ-DK. The IAM structures were re-refined with *Olex2.refine* based on the original structures.

			DiSCaMB-HAR						NoSpherA2-HAR					
**ZEYVAA**	**neutron**	**IAM**	**B3LYP**	**PBE**	**M06-2X**	**B3LYP-DKH2**	**PBE-DKH2**	**M06-2X-DKH2**	**B3LYP**	**PBE**	**M06-2X**	**B3LYP-DKH2**	**PBE-DKH2**	**M06-2X-DKH2**
**REFCODE [literature reference]**	**ZEYVAA02**	**ZEYVAA01**												
**Chemical formula**	$C_{14}H_{23}Cl_2NbSi_2$													
**Space group**	P nma													
**Temperature (K)**	100	173(2)												
**Wavelength [Å]**	0.5-5.0	0.71073												
**Theta range (deg)**	NA	2.46-29.96												
**sin(θ)/ λ Å^{-1}**	NA	0.70												
**Completeness**	NA	0.7989												
**R_{int}**	0.0599	0.1130												
**Year of publication**	2000	1999												
**Parameters**	199	140	140	140	140	140	140	140	140	140	140	140	140	140
**Goodness of fit**	1.19	0.99	1.00	0.98	0.99	0.98	1.00	1.00	0.99	1.00	0.99	1.00	1.00	1.00
**R[%] (reflections)**	6.20 (1424)	4.16 (2212)	4.08 (2212)	4.04 (2212)	4.02 (2212)	3.96 (2212)	3.96 (2212)	3.96 (2212)	3.97 (2212)	3.97 (2212)	3.86 (2212)	3.99 (2212)	3.98 (2212)	3.97 (2212)
**wR2[%] (reflections)**	9.8 (1537)	12.56 (2221)	12.76 (2221)	12.55 (2221)	12.43 (2221)	12.13 (2221)	12.20 (2221)	12.20 (2221)	12.01 (2221)	12.22 (2221)	11.73 (2221)	12.23 (2221)	12.20 (2221)	12.19 (2221)
**$\Delta\rho_{min/max}$ (eÅ^{-3})**	'not applicable"	-2.80/1.21	-2.76/1.20	-2.74/1.20	-2.72/1.21	-2.69/1.22	-2.69/1.22	-2.68/1.22	-2.71/1.21	-2.67/1.22	-2.61/1.24	-2.69/1.22	-2.69/1.22	-2.67/1.22
**Refined H positions**	all	all	all	all	all	all	all	all	all	all	all	all	all	all
**H thermal motions**	anis	iso	iso											

Table S8 Experimental and computational details of the neutron, X-ray IAM and X-ray HAR crystal structures of GOJNIF. HAR was performed with a cluster of charges and dipoles modeling crystal environment (DiSCaMB) and without a cluster (NoSpherA2). DFT calculations for HAR were performed with various functionals in the non-relativistic version (B3LYP, PBE and M06-2X) and with relativistic correction (B3LYP-DKH2, PBE-DKH2 and M06-2X-DKH2). Basis sets: [a] cc-pVTZ-DK; [b] jorge-DZP; [c] jorge-TZP; [d] jorge-DZP-DKH, [e] jorge-TZP-DKH. The IAM structures were re-refined with *Olex2.refine* based on the original structures.

			DiSCaMB-HAR						NoSpherA2-HAR					
**GOJNIF**	**neutron**	**IAM**	**B3LYP**	**PBE**	**M06-2X**	**B3LYP-DKH2**	**PBE-DKH2**	**M06-2X-DKH2**	**B3LYP**	**PBE**	**M06-2X**	**B3LYP-DKH2**	**PBE-DKH2**	**M06-2X-DKH2**
**REFCODE [literature reference]**	**GOJNIF**	**GOJNIF01**												
**Chemical formula**	$C_{39}H_{62}InN_4NiP_3$													
**Space group**	P $2_12_12_1$													
**Temperature (K)**	100(2)	100(2)												
**Wavelength [Å]**	0.60- 3.36	0.71073												
**Theta range (deg)**	7.352-78.740	2.29-36.35												
**sin(θ)/ λ $Å^{-1}$**	NA	0.83												
**Completeness**	0.437	0.9994												
**R_{int}**	NA	NA												
**Year of publication**	2019	2019												
**Parameters**	991	513	681 [a]<br>681 [b]<br>681 [c]	failed* [a]<br>681 [b]<br>681 [c]	failed* [a]<br>681 [b]<br>681 [c]	681 [a]<br>681 [d]<br>681 [e]	failed* [a]<br>681 [d]<br>681 [e]	681 [a]<br>681 [d]<br>681 [e]	681 [a]<br>681 [b]<br>681 [c]	681 [a]<br>681 [b]<br>681 [c]	681 [a]<br>681 [b]<br>681 [c]	681 [a]<br>681 [d]<br>681 [e]	681 [a]<br>681 [d]<br>681 [e]	681 [a]<br>681 [d]<br>681 [e]
**Goodness of fit**	0.57	1.05	1.05 [a]<br>1.05 [b]<br>1.05 [c]	failed* [a]<br>1.05 [b]<br>1.04 [c]	failed* [a]<br>1.05 [b]<br>1.04 [c]	1.05 [a]<br>1.05 [d]<br>1.05 [e]	failed* [a]<br>1.05 [d]<br>1.05 [e]	1.05 [a]<br>1.04 [d]<br>1.07 [e]	1.05 [a]<br>1.05 [b]<br>1.04 [c]	1.05 [a]<br>1.05 [b]<br>1.05 [c]	1.05 [a]<br>1.05 [b]<br>1.04 [c]	1.05 [a]<br>1.04 [d]<br>1.05 [e]	1.05 [a]<br>1.05 [d]<br>1.05 [e]	1.06 [a]<br>1.05 [d]<br>1.04 [e]
**R[%]**<br>**(reflections)**	6.20<br>(3111)	1.80<br>(18741)	1.56 [a]<br>2.02[b]<br>1.99[c]<br>(18741)	failed* [a]<br>2.01[b]<br>1.97[c]<br>(18741)	failed* [a]<br>2.02[b]<br>1.98[c]<br>(18741)	1.54[a]<br>1.91[d]<br>1.89[e]<br>(18741)	failed* [a]<br>1.90[d]<br>1.87[e]<br>(18741)	1.54[a]<br>1.91[d]<br>1.88[e]<br>(18741)	1.57[a]<br>1.96 [b]<br>1.92 [c]<br>(18741)	1.57[a]<br>1.95 [b]<br>196[c]<br>(18741)	1.56[a]<br>2.01[b]<br>1.97[c]<br>(18741)	1.55[a]<br>1.96[d]<br>1.88 [e]<br>(18741)	1.54[a]<br>1.96[d]<br>1.92[e]<br>(18741)	1.55[a]<br>1.96[d]<br>1.93[e]<br>(18741)
**wR2[%]**<br>**(reflections)**	14.79<br>(4718)	4.02<br>(19473)	2.74 [a]<br>4.68[b]<br>4.55[c]<br>(19473)	failed* [a]<br>4.62[b]<br>4.46[c]<br>(19473)	failed* [a]<br>4.67[b]<br>4.51[c]<br>(19473)	2.67[a]<br>4.33[d]<br>4.20[e]<br>(19473)	failed* [a]<br>4.29[d]<br>4.12[e]<br>(19473)	2.67[a]<br>4.29[d]<br>4.12[e]<br>(19473)	2.75[a]<br>4.48 [b]<br>4.34 [c]<br>(19473)	2.77[a]<br>4.47 [b]<br>4.48[c]<br>(19473)	2.75[a]<br>4.66[b]<br>4.51[c]<br>(19473)	2.71[a]<br>4.51[d]<br>4.18 [e]<br>(19473)	2.69[a]<br>4.48[d]<br>4.31[e]<br>(19473)	2.68[a]<br>4.48[d]<br>4.34[e]<br>(19473)
**$\Delta\rho_{min/max}$ ($eÅ^{-3}$)**	-0.80/0.80	-0.51/0.42	-0.49/0.36 [a]<br>-0.90/0.48 [b]<br>-0.85/0.49 [c]	failed* [a]<br>-0.89/0.47 [b]<br>-0.85/0.48 [c]	failed* [a]<br>-0.90/0.47 [b]<br>-0.86/0.49 [c]	-0.47/0.34 [a]<br>-0.81/0.47 [d]<br>-0.76/0.49 [e]	failed* [a]<br>-0.81/0.45 [d]<br>-0.76/0.47 [e]	-0.48/0.36 [a]<br>-0.81/0.46 [d]<br>-0.78/0.49 [e]	-0.48/0.35 [a]<br>-0.95/0.43 [b]<br>-0.91/0.45 [c]	-0.50/0.35 [a]<br>-0.95/0.43 [b]<br>-0.87/0.53 [c]	-0.49/0.37 [a]<br>-0.91/0.50 [b]<br>-0.87/0.53 [c]	-0.47/0.34 [a]<br>-0.82/0.51 [d]<br>-0.83/0.43 [e]	-0.46/0.33 [a]<br>-0.82/0.50 [d]<br>-0.77/0.53 [e]	-0.46/0.36 [a]<br>-0.82/0.50 [d]<br>-0.79/0.52 [e]
**Refined H positions**	all	H1A, H1B	all	all	all	all	all	all	all	all	all	all	all	all
**H thermal motions**	anis	iso	iso											

* Failure of wave function calculations.

Table S9 Experimental and computational details of the neutron, X-ray IAM and X-ray HAR crystal structures of TIWXOP. HAR was performed with a cluster of charges and dipoles modeling crystal environment (DiSCaMB) and without a cluster (NoSpherA2). DFT calculations for HAR were performed with various functionals in the non-relativistic version (B3LYP, PBE and M06-2X) and with relativistic correction (B3LYP-DKH2, PBE-DKH2 and M06-2X-DKH2). Basis sets: [a] cc-pVTZ-DK; [b] jorge-DZP; [c] jorge-TZP; [d] jorge-DZP-DKH, [e] jorge-TZP-DKH. The IAM structures were re-refined with *Olex2.refine* based on the original structures.

			**DiSCaMB-HAR**						**NoSpherA2-HAR**					
**TIWXOP**	**neutron**	**IAM**	**B3LYP**	**PBE**	**M06-2X**	**B3LYP-DKH2**	**PBE-DKH2**	**M06-2X-DKH2**	**B3LYP**	**PBE**	**M06-2X**	**B3LYP-DKH2**	**PBE-DKH2**	**M06-2X-DKH2**
**REFCODE [literature reference]**	**TIWXOP01**	**TIWXOP**												
**Chemical formula**	$C_{20}H_{31}N_2OSbSi_2$													
**Space group**	P -1													
**Temperature (K)**	120	120.01(10)												
**Wavelength [Å]**	0.85	0.71073												
**Theta range (deg)**	0.000-57.91	3.794- 28.961												
**sin(θ)/ λ Å^{-1}**	1.00	0.62												
**Completeness**	0.775	0.9978												
**R_{int}**	NA	NA												
**Year of publication**	2019	2019												
**Parameters**	489	359												
**Goodness of fit**	1.47	1.05	1.07 [a]<br>1.07 [b]<br>1.09 [c]	1.07 [a]<br>1.07 [b]<br>1.09 [c]	1.07 [a]<br>1.07 [b]<br>1.08 [c]	1.06 [a]<br>1.07 [d]<br>1.09 [e]	1.06 [a]<br>1.06 [d]<br>1.08 [e]	1.08 [a]<br>1.06 [d]<br>1.08 [e]	1.05 [a]<br>1.05 [b]<br>1.05 [c]	1.05 [a]<br>1.05 [b]<br>1.05 [c]	1.06 0[a]<br>1.05 [b]<br>1.05 [c]	1.05 [a]<br>1.05 [d]<br>1.05 [e]	1.05 [a]<br>1.05 [d]<br>1.05 [e]	1.05 [a]<br>1.05 [d]<br>1.05 [e]
**R[%]**<br>**(reflections)**	6.77<br>(1031)	3.03<br>(4181)	2.80 [a]<br>2.80[b]<br>2.86[c]<br>(4181)	2.79[a]<br>2.80[b]<br>2.84[c]<br>(4181)	2.80[a]<br>2.79[b]<br>2.84[c]<br>(4181)	2.78[a]<br>2.79[d]<br>2.86[e]<br>(4181)	2.78[a]<br>2.79[d]<br>2.84[e]<br>(4181)	2.83[a]<br>2.78[d]<br>2.83[e]<br>(4181)	2.78 [a]<br>2.81[b]<br>3.04[c]<br>(4181)	2.78 [a]<br>3.03[b]<br>3.04[c]<br>(4181)	2.81[a]<br>2.80[b]<br>3.04[c]<br>(4181)	2.78[a]<br>2.80[d]<br>3.04[e]<br>(4181)	2.77[a]<br>3.03[d]<br>3.04[e]<br>(4181)	2.78[a]<br>2.79[d]<br>3.04[e]<br>(4181)
**wR2[%]**<br>**(reflections)**	5.37<br>(1801)	6.36<br>(4543)	4.95 [a]<br>4.96[b]<br>5.06[c]<br>(4543)	4.94[a]<br>4.94[b]<br>5.03[c]<br>(4543)	4.95[a]<br>4.94[b]<br>5.02[c]<br>(4543)	4.91[a]<br>4.94[d]<br>5.05[e]<br>(4543)	4.90[a]<br>4.92[d]<br>5.02[e]<br>(4543)	5.02[a]<br>4.92[d]<br>5.01[e]<br>(4543)	5.08 [a]<br>5.15[b]<br>6.59[c]<br>(4543)	5.09 [a]<br>6.58[b]<br>6.62[c]<br>(4543)	5.31[a]<br>5.08[b]<br>6.59[c]<br>(4543)	5.09[a]<br>5.12[d]<br>6.59[e]<br>(4543)	5.09[a]<br>6.61[d]<br>6.62[e]<br>(4543)	5.08[a]<br>5.07[d]<br>6.59[e]<br>(4543)
**$\Delta\rho_{min/max}$ (eÅ^{-3})**	-0.63/0.58	-0.59/ 1.05	-0.58/ 1.11 [a]<br>-0.58/1.00 [b]<br>-0.60/0.98 [c]	-0.58/1.10 [a]<br>-0.58/1.01 [b]<br>-0.60/0.98 [c]	-0.58/1.11 [a]<br>-0.58/1.01 [b]<br>-0.59/0.98 [c]	-0.58/1.07 [a]<br>-0.58/1.01 [d]<br>-0.61/0.98 [e]	-0.58/1.06 [a]<br>-0.58/1.02 [d]<br>-0.60/0.97 [e]	-0.57/1.04 [a]<br>-0.58/1.02 [d]<br>-0.60/0.97 [e]	-0.58/0.99 [a]<br>-0.58/1.01 [b]<br>-0.59/0.93 [c]	-0.58/1.00 [a]<br>-0.59/0.96 [b]<br>-0.59/0.93 [c]	-0.58/1.09 [a]<br>-0.58/1.01 [b]<br>-0.59/0.93 [c]	-0.57/1.07 [a]<br>-0.58/1.01 [d]<br>-0.60/0.92 [e]	-0.56/1.08 [a]<br>-0.59/0.96 [d]<br>-0.59/0.91 [e]	-0.58/1.08 [a]<br>-0.57/1.02 [d]<br>-0.59/0.91 [e]
**Refined H positions**	all	Sb-H	all	all	all	all	all	all	all	all	all	all	all	all
**H thermal motions**	anis+iso(H81, H82, H83)	iso	iso											

Table S10 Experimental and computational details of the neutron, X-ray IAM and X-ray HAR crystal structures of XAXMEP. HAR was performed with a cluster of charges and dipoles modeling crystal environment (DiSCaMB) and without a cluster (NoSpherA2). DFT calculations for HAR were performed with various functionals in the non-relativistic version (B3LYP, PBE and M06-2X) and with relativistic correction (B3LYP-DKH2, PBE-DKH2 and M06-2X-DKH2). Basis sets: [a] jorge-DZP; [b] jorge-TZP; [c] jorge-DZP-DKH, [d] jorge-TZP-DKH. The IAM structures were re-refined with *Olex2.refine* based on the original structures.

			DiSCaMB-HAR						NoSpherA2-HAR					
**XAXMEP**	**neutron**	**IAM**	**B3LYP**	**PBE**	**M06-2X**	**B3LYP-DKH2**	**PBE-DKH2**	**M06-2X-DKH2**	**B3LYP**	**PBE**	**M06-2X**	**B3LYP-DKH2**	**PBE-DKH2**	**M06-2X-DKH2**
**REFCODE [literature reference]**	**XAXMEP[**	**XAXMEP01**												
**Chemical formula**	$C_{28}H_{52}OsP$, BF_4													
**Space group**	P -1													
**Temperature (K)**	20	199(2)												
**Wavelength [Å]**	0.7-4.2	0.71073 Å												
**Theta range (deg)**	N/A	2.14-28.28												
**sin(θ)/ λ $Å^{-1}$**	N/A	0.67												
**Completeness**	N/A	0.918												
**R_{int}**	N/A	0.0325												
**Year of publication**	2005	2007												
**Parameters**	308	332	523 [a]<br>522 [b]	522 [a]<br>522 [b]	522 [a]<br>522 [b]	523 [c]<br>522 [d]	523 [c]<br>522 [d]	523 [c]<br>522 [d]	522 [a]<br>522 [b]	522 [a]<br>521 [b]	522 [a]<br>521 [b]	522 [c]<br>522 [d]	522 [c]<br>522 [d]	522 [c]<br>522 [d]
**Goodness of fit**	1.72	1.0734	1.65 [a]<br>1.65 [b]	1.65[a]<br>1.65[b]	1.65 [a]<br>1.65 [b]	1.64[c]<br>1.64[d]	1.63 [c]<br>1.64 [d]	1.63 [c]<br>1.64 [d]	1.10 [a]<br>1.09 [b]	1.09 [a]<br>1.09 [b]	1.10 [a]<br>1.09 [b]	1.09 [c]<br>1.09 [d]	1.09 [c]<br>1.09 [d]	1.09 [c]<br>1.09 [d]
**R[%]**<br><br>**(reflections)**	12.9<br><br>(2197)	3.35<br><br>(5953)	3.33 [a]<br>0.03.33[b]<br>(5952)	3.32[a]<br>3.33[b]<br>(5952)	3.32[a]<br>3.33[b]<br>(5952)	3.30[c]<br>3.30[d]<br>(5952)	3.29[c]<br>3.30[d]<br>(5952)	3.29[c]<br>3.30[d]<br>(5952)	3.35 [a]<br>3.35 [b]<br>(5952)	3.35 [a]<br>3.35 [b]<br>(5952)	3.34 [a]<br>3.35 [b]<br>(5952)	3.28 [c]<br>3.28 [d]<br>(5952)	3.28 [c]<br>3.28 [d]<br>(5952)	3.27 [c]<br>3.28 [d]<br>(5952)
**wR2[%]**<br><br>**(reflections)**	7.5<br><br>(6629)	8.08<br><br>(6629)	6.81 [a]<br>6.81[b]<br>(6629)	6.80[a]<br>6.80[b]<br>(6629)	6.79[a]<br>6.80[b]<br>(6629)	6.74[c]<br>6.75[d]<br>(6629)	6.74[c]<br>6.74[d]<br>(6629)	6.73[c]<br>6.74[d]<br>(6629)	8.23 [a]<br>8.24 [b]<br>(6629)	8.25 [a]<br>8.22 [b]<br>(6629)	8.20 [a]<br>8.24 [b]<br>(6629)	7.92 [c]<br>7.93 [d]<br>(6629)	7.92 [c]<br>7.93 [d]<br>(6629)	7.90 [c]<br>7.92 [d]<br>(6629)
**$\Delta\rho_{min/max}$ (e$Å^{-3}$)**	N/A	-1.52/1.32	-1.54/1.00 [a]<br>-1.56/0.96 [b]	-1.54/1.02 [a]<br>-1.55/0.98 [b]	-1.54/1.02 [a]<br>-1.56/0.97 [b]	-1.48/1.01 [c]<br>-1.50/0.95 [d]	-1.48/1.02 [c]<br>-1.50/0.98 [d]	-1.48/1.03 [c]<br>-1.50/0.97 [d]	-1.58/0.94 [a]<br>-1.61/0.91 [b]	-1.60/0.95 [a]<br>-1.61/0.93 [b]	-1.58/0.96 [a]<br>-1.62/0.92[b]	-1.48/0.94 [c]<br>-1.48/0.92 [d]	-1.48/0.96 [c]<br>-1.48/0.92 [d]	-1.47/0.97 [c]<br>-1.49/0.93 [d]
**Refined H positions**	all	Os-H	all	all	all	all	all	all	all	all	all	all	all	all
**H thermal motions**	iso	iso	iso											

Table S11 X-H bond lengths (units: Å) obtained for QOSZON with various experimental methods (neutron and X-ray) and refinement methods (IAM and HAR). HAR was performed with a cluster of charges and dipoles modeling crystal environment (DiSCaMB) and without a cluster (NoSpherA2). DFT calculations for HAR were performed with various functionals in the non-relativistic version (B3LYP, PBE and M06-2X) and with relativistic correction (B3LYP-DKH2, PBE-DKH2 and M06-2X-DKH2). Basis sets used: (a) non-relativistic refinements: cc-pVDZ, (b) relativistic refinements: cc-pVDZ-DK.

			**DiSCaMB-HAR**						**NoSpherA2-HAR**					
**bond**	**neutron**	**IAM**	**B3LYP**	**PBE**	**M06-2X**	**B3LYP-DKH2**	**PBE-DKH2**	**M06-2X-DKH2**	**B3LYP**	**PBE**	**M06-2X**	**B3LYP-DKH2**	**PBE-DKH2**	**M06-2X-DKH2**
**Fe1 H1**	1.529(2)	1.44(2)	1.520(16)	1.525(15)	1.516(15)	1.522(16)	1.527(15)	1.519(15)	1.520(16)	1.520(16)	1.516(16)	1.520(16)	1.522(16)	1.517(16)
**Fe1 H2**	1.521(2)	1.42(2)	1.485(14)	1.492(13)	1.486(13)	1.485(14)	1.492(13)	1.485(13)	1.500(15)	1.501(15)	1.499(15)	1.501(15)	1.502(15)	1.499(15)
**C2 H3**	1.086(2)	0.96(2)	1.066(17)	1.070(17)	1.067(17)	1.066(17)	1.069(17)	1.067(17)	1.087(18)	1.087(18)	1.089(18)	1.087(18)	1.086(17)	1.089(18)
**C3 H4**	1.0856(19)	0.95(3)	1.100(18)	1.101(18)	1.099(18)	1.095(18)	1.097(18)	1.093(18)	1.096(19)	1.095(19)	1.093(19)	1.095(19)	1.096(19)	1.094(19)
**C4 H5**	1.087(2)	0.96(2)	1.091(19)	1.093(19)	1.092(19)	1.088(19)	1.090(19)	1.088(19)	1.086(19)	1.086(19)	1.086(19)	1.086(19)	1.085(19)	1.086(19)
**C5 H6**	1.088(2)	0.91(3)	1.120(15)	1.119(16)	1.117(16)	1.117(15)	1.117(15)	1.114(16)	1.104(16)	1.104(16)	1.101(17)	1.105(16)	1.101(16)	1.102(17)
**C6 H7**	1.0877(19)	0.95(2)	1.096(17)	1.098(17)	1.095(17)	1.095(17)	1.097(17)	1.093(17)	1.091(18)	1.091(18)	1.088(18)	1.091(18)	1.093(18)	1.089(18)
**C8 H8**	1.086(2)	0.96(2)	1.119(15)	1.120(15)	1.118(16)	1.116(15)	1.117(15)	1.115(16)	1.105(16)	1.105(16)	1.103(16)	1.105(16)	1.105(16)	1.103(16)
**C9 H9**	1.0856(19)	0.97(3)	1.095(18)	1.098(18)	1.097(18)	1.091(18)	1.094(18)	1.093(18)	1.083(19)	1.082(19)	1.083(19)	1.082(19)	1.083(19)	1.083(19)
**C10 H10**	1.088(2)	0.92(2)	1.085(14)	1.087(14)	1.085(14)	1.083(14)	1.085(14)	1.084(14)	1.080(14)	1.080(14)	1.080(15)	1.080(14)	1.080(14)	1.081(14)
**C11 H11**	1.089(2)	0.97(3)	1.101(17)	1.104(17)	1.103(17)	1.098(17)	1.101(17)	1.100(17)	1.087(18)	1.088(18)	1.091(18)	1.088(18)	1.090(18)	1.091(18)
**C12 H12**	1.0873(19)	0.94(2)	1.053(15)	1.055(15)	1.051(15)	1.052(15)	1.054(15)	1.050(15)	1.066(17)	1.066(17)	1.064(17)	1.066(17)	1.067(17)	1.064(17)
**C14 H13**	1.086(2)	0.94(3)	1.075(18)	1.077(18)	1.075(18)	1.076(18)	1.078(18)	1.076(18)	1.071(18)	1.071(18)	1.072(19)	1.071(18)	1.072(18)	1.073(18)
**C15 H14**	1.085(2)	0.97(2)	1.104(17)	1.106(17)	1.103(17)	1.100(17)	1.102(17)	1.099(17)	1.078(17)	1.078(17)	1.078(17)	1.078(17)	1.079(17)	1.078(17)
**C16 H15**	1.085(2)	0.95(3)	1.111(17)	1.115(17)	1.115(17)	1.108(17)	1.111(17)	1.111(17)	1.095(18)	1.095(18)	1.097(18)	1.095(18)	1.095(18)	1.097(18)
**C17 H16**	1.085(2)	0.94(3)	1.095(17)	1.098(17)	1.096(17)	1.090(17)	1.092(17)	1.090(17)	1.092(17)	1.092(17)	1.092(17)	1.092(17)	1.093(17)	1.092(17)
**C18 H17**	1.084(2)	0.94(2)	1.080(15)	1.084(15)	1.082(15)	1.082(15)	1.087(15)	1.085(15)	1.084(16)	1.084(16)	1.084(16)	1.084(16)	1.084(16)	1.085(16)
**C20 H18**	1.088(2)	0.96(2)	1.086(15)	1.088(15)	1.085(15)	1.082(15)	1.084(15)	1.080(15)	1.090(15)	1.090(15)	1.088(15)	1.091(15)	1.092(15)	1.089(15)
**C21 H19**	1.088(2)	0.91(3)	1.082(18)	1.086(18)	1.083(18)	1.077(18)	1.080(18)	1.077(18)	1.068(18)	1.068(18)	1.067(18)	1.069(18)	1.070(18)	1.068(18)
**C22 H20**	1.083(2)	0.90(3)	1.084(18)	1.085(18)	1.082(18)	1.080(18)	1.082(18)	1.079(18)	1.077(19)	1.077(19)	1.075(19)	1.078(19)	1.080(18)	1.076(19)
**C23 H21**	1.087(2)	0.99(2)	1.082(14)	1.085(14)	1.082(14)	1.082(14)	1.085(14)	1.082(14)	1.086(14)	1.087(14)	1.088(14)	1.087(14)	1.088(14)	1.088(14)
**C24 H22**	1.085(2)	0.95(2)	1.093(16)	1.096(16)	1.096(16)	1.091(16)	1.095(16)	1.094(16)	1.097(16)	1.097(16)	1.099(16)	1.097(16)	1.096(16)	1.099(16)
**C26 H23**	1.0845(19)	0.95(2)	1.089(19)	1.090(19)	1.09(2)	1.091(19)	1.093(19)	1.09(2)	1.101(19)	1.100(19)	1.099(19)	1.100(18)	1.101(18)	1.099(19)
**C27 H24**	1.085(2)	0.93(3)	1.07(2)	1.077(19)	1.073(19)	1.07(2)	1.071(19)	1.07(2)	1.08(2)	1.08(2)	1.07(2)	1.08(2)	1.08(2)	1.07(2)
**C28 H25**	1.085(2)	1.00(3)	1.118(17)	1.121(17)	1.119(17)	1.114(17)	1.118(17)	1.116(17)	1.100(17)	1.100(17)	1.101(17)	1.101(16)	1.103(16)	1.101(17)
**C29 H26**	1.086(2)	0.95(2)	1.096(17)	1.096(17)	1.093(18)	1.094(17)	1.094(17)	1.091(18)	1.094(18)	1.094(18)	1.091(18)	1.095(18)	1.094(18)	1.092(18)

**C30**	**H27**	1.083(2)	0.94(2)	1.085(16)	1.088(16)	1.085(16)	1.084(16)	1.086(16)	1.084(16)	1.078(17)	1.078(17)	1.078(17)	1.079(17)	1.079(17)	1.078(17)
**C32**	**H28**	1.084(2)	0.98(2)	1.087(18)	1.089(18)	1.083(18)	1.090(17)	1.092(17)	1.086(18)	1.111(17)	1.112(17)	1.110(17)	1.111(17)	1.111(17)	1.110(17)
**C33**	**H29**	1.088(2)	0.92(3)	1.107(19)	1.110(19)	1.108(19)	1.103(19)	1.106(19)	1.103(19)	1.095(19)	1.095(19)	1.094(19)	1.096(19)	1.097(19)	1.095(19)
**C34**	**H30**	1.087(2)	0.94(2)	1.091(16)	1.093(16)	1.093(16)	1.087(16)	1.089(16)	1.089(16)	1.076(16)	1.076(16)	1.078(16)	1.076(15)	1.075(15)	1.078(15)
**C35**	**H31**	1.085(2)	0.96(2)	1.073(16)	1.076(16)	1.072(16)	1.070(16)	1.072(16)	1.068(16)	1.076(16)	1.077(16)	1.076(16)	1.076(16)	1.079(16)	1.076(16)
**C36**	**H32**	1.083(2)	0.95(2)	1.087(18)	1.089(18)	1.085(18)	1.084(18)	1.086(18)	1.083(18)	1.098(18)	1.098(17)	1.096(18)	1.097(17)	1.098(17)	1.095(17)

Table S12 X-H bond lengths (units: Å) obtained for QOSZON with various experimental methods (neutron and X-ray) and refinement methods (IAM and HAR). HAR was performed with a cluster of charges and dipoles modeling crystal environment (DiSCaMB) and without a cluster (NoSpherA2). DFT calculations for HAR were performed with various functionals in the non-relativistic version (B3LYP, PBE and M06-2X) and with relativistic correction (B3LYP-DKH2, PBE-DKH2 and M06-2X-DKH2). Basis sets used: (a) non-relativistic refinements: cc-pVTZ, (b) relativistic refinements: cc-pVTZ-DK.

			**DiSCaMB-HAR**						**NoSpherA2-HAR**					
**bond**	**neutron**	**IAM**	**B3LYP**	**PBE**	**M06-2X**	**B3LYP-DKH2**	**PBE-DKH2**	**M06-2X-DKH2**	**B3LYP**	**PBE**	**M06-2X**	**B3LYP-DKH2**	**PBE-DKH2**	**M06-2X-DKH2**
**Fe1 H1**	1.529(2)	1.44(2)	1.522(15)	1.526(15)	1.521(15)	1.523(16)	1.528(15)	1.524(15)	1.523(16)	1.526(15)	1.524(15)	1.524(15)	1.526(15)	1.524(15)
**Fe1 H2**	1.521(2)	1.42(2)	1.505(14)	1.497(13)	1.494(13)	1.490(14)	1.497(13)	1.493(13)	1.505(15)	1.506(14)	1.505(14)	1.506(15)	1.507(14)	1.506(14)
**C2 H3**	1.086(2)	0.96(2)	1.083(17)	1.067(16)	1.063(16)	1.061(16)	1.067(16)	1.063(16)	1.084(17)	1.084(16)	1.086(17)	1.084(17)	1.084(16)	1.086(17)
**C3 H4**	1.0856(19)	0.95(3)	1.108(18)	1.108(18)	1.104(18)	1.101(18)	1.102(18)	1.098(18)	1.101(18)	1.102(18)	1.098(18)	1.102(18)	1.102(18)	1.099(18)
**C4 H5**	1.087(2)	0.96(2)	1.093(18)	1.093(18)	1.092(19)	1.092(18)	1.092(18)	1.091(19)	1.091(18)	1.089(18)	1.089(18)	1.091(18)	1.088(18)	1.089(18)
**C5 H6**	1.088(2)	0.91(3)	1.109(16)	1.120(15)	1.118(15)	1.118(15)	1.117(15)	1.115(15)	1.106(16)	1.103(16)	1.103(16)	1.107(16)	1.103(16)	1.104(16)
**C6 H7**	1.0877(19)	0.95(2)	1.096(17)	1.099(17)	1.095(17)	1.096(16)	1.098(16)	1.093(17)	1.094(17)	1.095(17)	1.090(17)	1.095(17)	1.096(17)	1.091(17)
**C8 H8**	1.086(2)	0.96(2)	1.112(15)	1.125(15)	1.122(15)	1.122(15)	1.122(15)	1.119(15)	1.109(15)	1.109(15)	1.107(16)	1.109(15)	1.109(15)	1.107(15)
**C9 H9**	1.0856(19)	0.97(3)	1.087(18)	1.099(17)	1.097(17)	1.090(17)	1.094(17)	1.093(17)	1.083(19)	1.084(18)	1.084(18)	1.083(18)	1.084(18)	1.084(18)
**C10 H10**	1.088(2)	0.92(2)	1.084(14)	1.089(14)	1.087(14)	1.085(14)	1.087(14)	1.085(14)	1.083(14)	1.083(14)	1.083(14)	1.083(14)	1.083(13)	1.083(14)
**C11 H11**	1.089(2)	0.97(3)	1.093(17)	1.105(16)	1.104(16)	1.098(16)	1.101(16)	1.100(16)	1.088(17)	1.090(17)	1.091(17)	1.089(17)	1.091(17)	1.092(17)
**C12 H12**	1.0873(19)	0.94(2)	1.067(16)	1.055(15)	1.050(15)	1.051(15)	1.055(15)	1.049(15)	1.066(16)	1.066(16)	1.063(16)	1.066(16)	1.067(16)	1.063(16)
**C14 H13**	1.086(2)	0.94(3)	1.076(18)	1.081(17)	1.078(17)	1.081(17)	1.081(17)	1.079(17)	1.075(18)	1.077(17)	1.075(18)	1.076(18)	1.077(17)	1.076(18)
**C15 H14**	1.085(2)	0.97(2)	1.085(17)	1.105(16)	1.101(16)	1.099(16)	1.100(16)	1.097(16)	1.080(17)	1.081(16)	1.080(17)	1.080(16)	1.081(16)	1.080(17)
**C16 H15**	1.085(2)	0.95(3)	1.105(17)	1.119(17)	1.118(17)	1.111(17)	1.114(17)	1.114(17)	1.100(17)	1.100(17)	1.102(17)	1.100(17)	1.100(17)	1.102(17)
**C17 H16**	1.085(2)	0.94(3)	1.095(16)	1.097(16)	1.095(16)	1.087(16)	1.089(16)	1.087(16)	1.089(16)	1.090(16)	1.090(16)	1.089(16)	1.090(16)	1.090(16)
**C18 H17**	1.084(2)	0.94(2)	1.087(15)	1.089(14)	1.087(15)	1.087(14)	1.092(14)	1.090(15)	1.091(16)	1.090(15)	1.091(16)	1.091(15)	1.090(15)	1.092(16)
**C20 H18**	1.088(2)	0.96(2)	1.098(14)	1.092(14)	1.087(14)	1.085(14)	1.087(14)	1.082(14)	1.093(14)	1.094(14)	1.090(14)	1.094(14)	1.094(14)	1.090(14)
**C21 H19**	1.088(2)	0.91(3)	1.082(17)	1.091(17)	1.086(17)	1.082(17)	1.085(17)	1.081(17)	1.077(17)	1.078(17)	1.075(17)	1.077(17)	1.079(17)	1.076(17)
**C22 H20**	1.083(2)	0.90(3)	1.085(18)	1.089(18)	1.084(18)	1.084(18)	1.087(18)	1.081(18)	1.081(18)	1.083(18)	1.079(18)	1.082(18)	1.084(18)	1.079(18)
**C23 H21**	1.087(2)	0.99(2)	1.089(14)	1.086(14)	1.082(14)	1.082(14)	1.086(14)	1.082(14)	1.089(14)	1.089(14)	1.088(14)	1.089(14)	1.089(14)	1.088(14)
**C24 H22**	1.085(2)	0.95(2)	1.097(16)	1.095(16)	1.094(16)	1.090(16)	1.094(16)	1.092(16)	1.097(16)	1.095(16)	1.098(16)	1.096(16)	1.095(16)	1.098(16)
**C26 H23**	1.0845(19)	0.95(2)	1.095(18)	1.090(19)	1.086(19)	1.090(19)	1.093(19)	1.088(19)	1.099(18)	1.100(18)	1.097(18)	1.099(18)	1.100(18)	1.097(18)
**C27 H24**	1.085(2)	0.93(3)	1.09(2)	1.081(19)	1.076(19)	1.072(19)	1.075(19)	1.070(19)	1.08(2)	1.081(19)	1.08(2)	1.08(2)	1.082(19)	1.08(2)
**C28 H25**	1.085(2)	1.00(3)	1.111(16)	1.127(16)	1.123(16)	1.119(16)	1.122(16)	1.120(16)	1.107(16)	1.109(16)	1.107(16)	1.108(16)	1.110(16)	1.107(16)
**C29 H26**	1.086(2)	0.95(2)	1.099(17)	1.096(17)	1.092(17)	1.094(17)	1.095(17)	1.091(17)	1.098(17)	1.097(17)	1.094(17)	1.098(17)	1.097(17)	1.094(17)

**C30**	**H27**	1.083(2)	0.94(2)	1.084(16)	1.089(16)	1.085(16)	1.084(16)	1.087(16)	1.084(16)	1.082(16)	1.083(16)	1.082(16)	1.082(16)	1.083(16)	1.082(16)
**C32**	**H28**	1.084(2)	0.98(2)	1.112(16)	1.094(17)	1.088(17)	1.095(17)	1.098(17)	1.092(17)	1.115(16)	1.114(16)	1.113(16)	1.115(16)	1.114(16)	1.113(16)
**C33**	**H29**	1.088(2)	0.92(3)	1.101(18)	1.110(18)	1.106(18)	1.104(18)	1.106(18)	1.102(18)	1.096(18)	1.098(18)	1.094(18)	1.096(18)	1.098(18)	1.094(18)
**C34**	**H30**	1.087(2)	0.94(2)	1.082(15)	1.094(15)	1.094(15)	1.087(15)	1.089(15)	1.089(15)	1.078(15)	1.077(15)	1.080(15)	1.078(15)	1.077(15)	1.080(15)
**C35**	**H31**	1.085(2)	0.96(2)	1.080(16)	1.075(16)	1.071(16)	1.070(16)	1.072(16)	1.068(16)	1.077(16)	1.079(15)	1.076(16)	1.077(16)	1.079(15)	1.076(16)
**C36**	**H32**	1.083(2)	0.95(2)	1.101(17)	1.090(18)	1.085(18)	1.086(18)	1.088(18)	1.084(18)	1.099(17)	1.100(17)	1.096(17)	1.099(17)	1.099(17)	1.096(17)

Table S13 X-H bond lengths (units: Å) obtained for NEBNEO with various experimental methods (neutron and X-ray) and refinement methods (IAM and HAR). HAR was performed with a cluster of charges and dipoles modeling crystal environment (DiSCaMB) and without a cluster (NoSpherA2). DFT calculations for HAR were performed with various functionals in the non-relativistic version (B3LYP, PBE and M06-2X) and with relativistic correction (B3LYP-DKH2, PBE-DKH2 and M06-2X-DKH2). Basis sets used: (a) non-relativistic refinements: cc-pVTZ-DK, (b) relativistic refinements: cc-pVTZ-DK.

				**DiSCaMB-HAR**						**NoSpherA2-HAR**					
**bond**		**neutron**	**IAM**	**B3LYP**	**PBE**	**M06-2X**	**B3LYP-DKH2**	**PBE-DKH2**	**M06-2X-DKH2**	**B3LYP**	**PBE**	**M06-2X**	**B3LYP-DKH2**	**PBE-DKH2**	**M06-2X-DKH2**
**C1**	**H1**	1.100(4)	0.968(18)	1.120(18)	1.121(18)	1.120(18)	1.117(18)	1.118(18)	1.117(18)	1.10(2)	1.10(2)	1.10(2)	1.10(2)	1.10(2)	1.10(2)
**C2**	**H2A**	1.092(4)	0.939(19)	1.068(19)	1.071(19)	1.068(19)	1.066(19)	1.069(19)	1.067(19)	1.06(2)	1.06(2)	1.06(2)	1.06(2)	1.06(2)	1.06(2)
**C2**	**H2B**	1.103(4)	0.97(2)	1.134(19)	1.135(19)	1.132(19)	1.134(19)	1.136(19)	1.132(19)	1.11(2)	1.11(2)	1.11(2)	1.11(2)	1.11(2)	1.11(2)
**C3**	**H3A**	1.092(5)	1.03(2)	1.127(19)	1.132(19)	1.130(19)	1.127(19)	1.132(19)	1.129(19)	1.12(2)	1.12(2)	1.12(2)	1.11(2)	1.12(2)	1.11(2)
**C3**	**H3B**	1.095(4)	0.95(2)	1.10(2)	1.10(2)	1.10(2)	1.09(2)	1.10(2)	1.10(2)	1.09(3)	1.10(3)	1.09(3)	1.09(3)	1.09(3)	1.09(3)
**C4**	**H4A**	1.101(5)	0.98(2)	1.118(18)	1.120(18)	1.118(18)	1.122(18)	1.124(18)	1.122(18)	1.14(2)	1.14(2)	1.14(2)	1.13(2)	1.13(2)	1.13(2)
**C4**	**H4B**	1.097(4)	0.956(19)	1.04(2)	1.044(19)	1.041(19)	1.04(2)	1.039(19)	1.036(19)	1.09(3)	1.09(3)	1.09(3)	1.09(3)	1.09(3)	1.09(3)
**C5**	**H5A**	1.091(4)	0.947(19)	1.09(2)	1.09(2)	1.09(2)	1.09(2)	1.090(19)	1.087(19)	1.09(2)	1.08(2)	1.08(2)	1.08(2)	1.08(2)	1.08(2)
**C5**	**H5B**	1.100(5)	0.97(2)	1.09(2)	1.09(2)	1.09(2)	1.09(2)	1.09(2)	1.09(2)	1.06(2)	1.06(2)	1.06(2)	1.06(2)	1.06(2)	1.06(2)
**C6**	**H6**	1.102(4)	0.968(19)	1.101(17)	1.102(17)	1.102(17)	1.100(17)	1.102(17)	1.101(17)	1.09(2)	1.09(2)	1.09(2)	1.09(2)	1.09(2)	1.09(2)
**C7**	**H7A**	1.092(4)	0.99(2)	1.093(19)	1.098(18)	1.095(18)	1.092(18)	1.097(18)	1.094(18)	1.08(2)	1.09(2)	1.09(2)	1.08(2)	1.09(2)	1.09(2)
**C7**	**H7B**	1.100(4)	0.96(2)	1.081(19)	1.083(18)	1.080(18)	1.080(19)	1.082(18)	1.080(18)	1.10(2)	1.10(2)	1.10(2)	1.10(2)	1.10(2)	1.10(2)
**C8**	**H8A**	1.091(5)	0.94(2)	1.08(2)	1.08(2)	1.08(2)	1.08(2)	1.08(2)	1.08(2)	1.13(3)	1.13(3)	1.13(3)	1.13(3)	1.13(3)	1.13(3)
**C8**	**H8B**	1.090(4)	0.94(2)	1.10(2)	1.10(2)	1.10(2)	1.10(2)	1.10(2)	1.10(2)	1.10(2)	1.10(2)	1.10(2)	1.10(2)	1.10(2)	1.10(2)
**C9**	**H9A**	1.087(5)	0.92(2)	1.07(2)	1.06(2)	1.06(2)	1.06(2)	1.06(2)	1.06(2)	1.08(3)	1.08(3)	1.08(3)	1.08(3)	1.08(3)	1.08(3)
**C9**	**H9B**	1.093(4)	0.88(2)	1.01(3)	1.01(3)	1.01(3)	1.00(3)	1.01(2)	1.00(2)	1.03(3)	1.03(3)	1.03(3)	1.03(3)	1.03(3)	1.03(3)
**C10**	**H10A**	1.086(5)	0.91(2)	1.06(2)	1.06(2)	1.05(2)	1.06(2)	1.06(2)	1.05(2)	1.06(3)	1.06(3)	1.06(3)	1.06(3)	1.06(3)	1.06(3)
**C10**	**H10B**	1.088(5)	0.97(2)	1.11(2)	1.12(2)	1.11(2)	1.11(2)	1.11(2)	1.11(2)	1.12(2)	1.12(2)	1.12(2)	1.12(2)	1.12(2)	1.12(2)
**C11**	**H11**	1.098(4)	0.94(2)	1.089(18)	1.091(18)	1.087(18)	1.085(18)	1.086(18)	1.083(18)	1.10(2)	1.10(2)	1.10(2)	1.10(2)	1.10(2)	1.10(2)
**C12**	**H12A**	1.096(4)	0.97(2)	1.092(19)	1.096(19)	1.092(19)	1.088(19)	1.091(19)	1.087(19)	1.11(2)	1.11(2)	1.11(2)	1.11(2)	1.11(2)	1.11(2)
**C12**	**H12B**	1.095(4)	0.97(2)	1.10(2)	1.10(2)	1.10(2)	1.10(2)	1.10(2)	1.10(2)	1.12(2)	1.12(2)	1.12(2)	1.12(2)	1.12(2)	1.12(2)
**C13**	**H13A**	1.084(5)	0.95(2)	1.10(2)	1.11(2)	1.11(2)	1.10(2)	1.11(2)	1.11(2)	1.07(3)	1.07(3)	1.06(3)	1.06(3)	1.06(3)	1.06(3)
**C13**	**H13B**	1.092(4)	0.97(3)	1.10(4)	1.10(3)	1.09(3)	1.10(3)	1.10(3)	1.09(3)	1.15(4)	1.15(4)	1.14(4)	1.14(4)	1.14(4)	1.14(4)
**C14**	**H14A**	1.088(5)	0.90(3)	1.07(3)	1.08(3)	1.07(3)	1.07(3)	1.07(3)	1.07(3)	1.07(3)	1.07(3)	1.07(3)	1.07(3)	1.07(3)	1.07(3)
**C14**	**H14B**	1.090(5)	1.01(3)	1.13(3)	1.13(3)	1.13(3)	1.13(3)	1.13(3)	1.13(3)	1.15(4)	1.15(4)	1.15(4)	1.15(4)	1.15(4)	1.14(4)
**C15**	**H15A**	1.089(5)	0.89(2)	1.04(2)	1.04(2)	1.04(2)	1.04(2)	1.04(2)	1.04(2)	1.05(3)	1.05(3)	1.05(3)	1.05(3)	1.05(3)	1.05(3)

**C15**	**H15B**	1.085(4)	0.96(2)	1.08(2)	1.08(2)	1.08(2)	1.08(2)	1.08(2)	1.07(2)	1.10(3)	1.10(3)	1.10(3)	1.10(3)	1.10(3)	1.10(3)
**C16**	**H16**	1.100(4)	0.968(18)	1.109(17)	1.112(17)	1.110(17)	1.107(17)	1.110(17)	1.108(17)	1.10(2)	1.09(2)	1.10(2)	1.10(2)	1.09(2)	1.09(2)
**C17**	**H17A**	1.094(4)	0.941(19)	1.064(18)	1.068(18)	1.066(18)	1.060(18)	1.064(18)	1.061(18)	1.06(2)	1.06(2)	1.06(2)	1.06(2)	1.06(2)	1.06(2)
**C17**	**H17B**	1.094(4)	1.005(19)	1.127(19)	1.128(19)	1.126(19)	1.124(19)	1.125(18)	1.124(18)	1.15(2)	1.15(2)	1.15(2)	1.15(2)	1.15(2)	1.15(2)
**C18**	**H18A**	1.090(4)	0.96(2)	1.08(2)	1.09(2)	1.08(2)	1.08(2)	1.08(2)	1.08(2)	1.10(3)	1.10(3)	1.10(3)	1.10(3)	1.10(3)	1.10(3)
**C18**	**H18B**	1.091(5)	0.92(2)	1.07(2)	1.07(2)	1.07(2)	1.06(2)	1.07(2)	1.06(2)	1.08(2)	1.08(2)	1.07(2)	1.08(2)	1.08(2)	1.07(2)
**C19**	**H19A**	1.091(4)	0.97(2)	1.06(3)	1.06(3)	1.06(3)	1.06(3)	1.06(3)	1.06(3)	1.08(3)	1.08(3)	1.08(3)	1.08(3)	1.08(3)	1.08(3)
**C19**	**H19B**	1.089(5)	0.92(2)	1.08(3)	1.08(3)	1.08(3)	1.08(3)	1.08(3)	1.07(3)	1.11(3)	1.11(3)	1.11(3)	1.10(3)	1.10(3)	1.10(3)
**C20**	**H20A**	1.091(4)	0.94(2)	1.07(2)	1.08(2)	1.07(2)	1.07(2)	1.07(2)	1.07(2)	1.07(3)	1.07(3)	1.07(3)	1.07(3)	1.07(3)	1.07(3)
**C20**	**H20B**	1.087(5)	0.88(2)	1.03(2)	1.03(2)	1.02(2)	1.02(2)	1.02(2)	1.02(2)	1.03(3)	1.03(3)	1.03(3)	1.03(3)	1.03(3)	1.02(3)
**C21**	**H21**	1.108(4)	0.952(17)	1.107(16)	1.107(16)	1.105(16)	1.105(16)	1.105(16)	1.103(16)	1.10(2)	1.10(2)	1.10(2)	1.10(2)	1.09(2)	1.09(2)
**C22**	**H22A**	1.084(4)	0.93(2)	1.10(2)	1.10(2)	1.10(2)	1.10(2)	1.10(2)	1.09(2)	1.10(2)	1.09(2)	1.09(2)	1.09(2)	1.09(2)	1.09(2)
**C22**	**H22B**	1.083(5)	0.972(19)	1.047(19)	1.054(19)	1.051(19)	1.050(19)	1.057(19)	1.053(19)	1.08(2)	1.08(2)	1.08(2)	1.08(2)	1.08(2)	1.08(2)
**C23**	**H23A**	1.094(4)	0.99(2)	1.13(2)	1.14(2)	1.13(2)	1.13(2)	1.14(2)	1.14(2)	1.13(3)	1.13(3)	1.13(3)	1.13(3)	1.13(3)	1.12(3)
**C23**	**H23B**	1.084(5)	0.96(2)	1.11(3)	1.12(2)	1.11(2)	1.11(2)	1.11(2)	1.11(2)	1.09(3)	1.09(3)	1.09(3)	1.09(3)	1.09(3)	1.09(3)
**C24**	**H24A**	1.092(4)	0.937(17)	1.09(2)	1.09(2)	1.09(2)	1.09(2)	1.09(2)	1.09(2)	1.11(2)	1.11(2)	1.10(2)	1.11(2)	1.10(2)	1.10(2)
**C24**	**H24B**	1.091(4)	0.959(19)	1.078(18)	1.081(18)	1.079(18)	1.076(18)	1.079(18)	1.077(18)	1.08(2)	1.08(2)	1.08(2)	1.08(2)	1.08(2)	1.08(2)
**C25**	**H25A**	1.088(4)	0.98(2)	1.15(2)	1.15(2)	1.14(2)	1.15(2)	1.148(19)	1.145(19)	1.11(2)	1.12(2)	1.11(2)	1.11(2)	1.11(2)	1.11(2)
**C25**	**H25B**	1.092(4)	0.999(18)	1.094(18)	1.097(18)	1.093(18)	1.092(18)	1.094(18)	1.091(18)	1.10(2)	1.10(2)	1.10(2)	1.10(2)	1.10(2)	1.10(2)
**C26**	**H26**	1.096(4)	0.981(18)	1.130(17)	1.134(17)	1.131(17)	1.127(17)	1.130(17)	1.128(17)	1.11(2)	1.107(19)	1.107(19)	1.11(2)	1.105(19)	1.105(19)
**C27**	**H27A**	1.096(5)	0.97(2)	1.085(19)	1.089(19)	1.087(19)	1.085(19)	1.088(19)	1.086(19)	1.07(2)	1.07(2)	1.07(2)	1.07(2)	1.07(2)	1.07(2)
**C27**	**H27B**	1.102(4)	0.938(19)	1.056(18)	1.061(18)	1.056(18)	1.055(18)	1.059(18)	1.055(18)	1.07(2)	1.07(2)	1.07(2)	1.07(2)	1.07(2)	1.06(2)
**C28**	**H28A**	1.093(4)	0.94(2)	1.08(2)	1.08(2)	1.08(2)	1.07(2)	1.07(2)	1.07(2)	1.08(3)	1.08(3)	1.08(3)	1.08(3)	1.08(3)	1.08(3)
**C28**	**H28B**	1.096(5)	0.97(2)	1.11(2)	1.12(2)	1.12(2)	1.11(2)	1.12(2)	1.11(2)	1.10(2)	1.10(2)	1.10(2)	1.10(2)	1.10(2)	1.10(2)
**C29**	**H29A**	1.087(4)	0.99(2)	1.12(2)	1.13(2)	1.12(2)	1.12(2)	1.12(2)	1.12(2)	1.11(2)	1.11(2)	1.11(2)	1.11(2)	1.11(2)	1.11(2)
**C29**	**H29B**	1.102(5)	0.96(2)	1.085(19)	1.087(19)	1.084(19)	1.087(19)	1.089(19)	1.087(19)	1.08(3)	1.08(3)	1.08(2)	1.08(3)	1.08(3)	1.08(2)
**C30**	**H30A**	1.096(5)	0.981(18)	1.160(17)	1.161(17)	1.158(17)	1.158(17)	1.159(17)	1.156(17)	1.11(2)	1.11(2)	1.11(2)	1.11(2)	1.11(2)	1.11(2)
**C30**	**H30B**	1.102(4)	0.948(19)	1.06(2)	1.067(19)	1.064(19)	1.065(19)	1.066(19)	1.064(19)	1.09(2)	1.09(2)	1.08(2)	1.08(2)	1.08(2)	1.08(2)
**Ru1**	**H21H**	1.764(5)	1.73(3)	1.78(2)	1.78(2)	1.78(2)	1.78(2)	1.78(2)	1.78(2)	1.79(3)	1.79(3)	1.79(3)	1.78(3)	1.78(3)	1.78(3)
**Ru1**	**H12H**	1.753(5)	1.73(3)	1.79(2)	1.80(2)	1.79(2)	1.79(2)	1.79(2)	1.79(2)	1.81(3)	1.80(3)	1.80(3)	1.80(3)	1.80(3)	1.80(3)
**Ru1**	**H1H**	1.628(4)	1.55(2)	1.55(2)	1.56(2)	1.57(2)	1.54(2)	1.55(2)	1.56(2)	1.56(2)	1.56(2)	1.57(2)	1.56(2)	1.56(2)	1.57(2)
**Ru1**	**H2H**	1.625(4)	1.55(2)	1.634(17)	1.640(17)	1.642(17)	1.634(18)	1.640(17)	1.643(17)	1.58(2)	1.58(2)	1.59(2)	1.58(2)	1.58(2)	1.58(2)
**Ru1**	**H22H**	1.745(5)	1.72(3)	1.77(2)	1.77(2)	1.77(2)	1.76(2)	1.77(2)	1.76(2)	1.72(3)	1.72(3)	1.72(3)	1.72(3)	1.72(3)	1.72(3)
**Ru1**	**H11H**	1.730(5)	1.70(2)	1.734(18)	1.739(18)	1.739(18)	1.731(18)	1.736(18)	1.737(18)	1.71(2)	1.71(2)	1.71(2)	1.71(2)	1.71(2)	1.71(2)

Table S14 X-H bond lengths (units: Å) obtained for MIGKIY with various experimental methods (neutron and X-ray) and refinement methods (IAM and HAR). HAR was performed with a cluster of charges and dipoles modeling crystal environment (DiSCaMB) and without a cluster (NoSpherA2). DFT calculations for HAR were performed with various functionals in the non-relativistic version (B3LYP, PBE and M06-2X) and with relativistic correction (B3LYP-DKH2, PBE-DKH2 and M06-2X-DKH2). Basis sets used: (a) non-relativistic refinements: cc-pVTZ-DK, (b) relativistic refinements: cc-pVTZ-DK.

				**DiSCaMB-HAR**						**NoSpherA2-HAR**					
**bond**		**neutron**	**IAM**	**B3LYP**	**PBE**	**M06-2X**	**B3LYP-DKH2**	**PBE-DKH2**	**M06-2X-DKH2**	**B3LYP**	**PBE**	**M06-2X**	**B3LYP-DKH2**	**PBE-DKH2**	**M06-2X-DKH2**
**C1**	**H1**	1.072(8)	0.950(3)	1.13(4)	1.13(4)	1.12(4)	1.12(4)	1.12(4)	1.12(4)	1.12(4)	1.12(4)	1.12(4)	1.12(4)	1.12(4)	1.12(4)
**C2**	**H2**	1.071(11)	0.949(3)	1.03(4)	1.03(4)	1.03(4)	1.03(4)	1.03(4)	1.03(4)	1.03(4)	1.03(4)	1.03(4)	1.03(4)	1.03(4)	1.03(4)
**C3**	**H3**	1.082(9)	0.949(3)	1.11(4)	1.11(4)	1.11(4)	1.11(4)	1.11(4)	1.11(4)	1.11(4)	1.11(4)	1.11(4)	1.11(4)	1.11(4)	1.11(4)
**C4**	**H4**	1.090(8)	0.950(3)	1.07(5)	1.07(5)	1.07(5)	1.07(5)	1.07(5)	1.06(5)	1.07(5)	1.07(5)	1.06(5)	1.06(5)	1.06(5)	1.06(5)
**C6**	**H6A**	1.088(9)	0.980(4)	1.13(5)	1.13(5)	1.14(5)	1.13(5)	1.14(5)	1.14(5)	1.13(5)	1.13(5)	1.14(5)	1.13(5)	1.13(5)	1.14(5)
**C6**	**H6B**	1.088(12)	0.981(4)	1.09(5)	1.09(5)	1.08(5)	1.09(5)	1.08(5)	1.08(5)	1.09(5)	1.09(5)	1.08(5)	1.08(5)	1.08(5)	1.08(5)
**C6**	**H6C**	1.096(10)	0.980(4)	1.13(5)	1.13(5)	1.12(5)	1.13(5)	1.13(5)	1.12(5)	1.13(5)	1.13(5)	1.13(5)	1.13(5)	1.12(5)	1.12(5)
**C7**	**H7A**	1.091(12)	0.981(5)	1.13(6)	1.13(6)	1.14(6)	1.14(6)	1.13(6)	1.14(6)	1.13(6)	1.13(6)	1.14(6)	1.13(6)	1.13(6)	1.14(6)
**C7**	**H7B**	1.058(13)	0.981(5)	1.08(6)	1.09(6)	1.09(6)	1.08(6)	1.09(6)	1.08(6)	1.08(6)	1.09(6)	1.08(6)	1.08(6)	1.09(6)	1.09(6)
**C7**	**H7C**	1.100(11)	0.979(4)	1.01(5)	1.01(5)	1.01(5)	1.01(5)	1.01(5)	1.00(5)	1.00(5)	1.00(5)	1.00(5)	1.01(5)	1.01(5)	1.00(5)
**C8**	**H8A**	1.078(9)	0.980(4)	1.07(4)	1.07(4)	1.07(4)	1.07(5)	1.07(4)	1.07(5)	1.07(4)	1.07(4)	1.07(4)	1.07(4)	1.07(4)	1.07(4)
**C8**	**H8B**	1.086(10)	0.980(4)	1.16(5)	1.17(5)	1.16(5)	1.16(5)	1.17(5)	1.16(5)	1.16(5)	1.16(5)	1.16(5)	1.16(5)	1.16(5)	1.16(5)
**C8**	**H8C**	1.095(10)	0.981(4)	1.04(4)	1.04(4)	1.04(4)	1.04(4)	1.04(4)	1.04(4)	1.04(4)	1.04(4)	1.04(4)	1.04(4)	1.04(4)	1.03(4)
**C11**	**H11**	1.094(8)	0.949(3)	1.10(3)	1.11(3)	1.10(3)	1.10(3)	1.10(3)	1.10(3)	1.10(3)	1.10(3)	1.10(3)	1.10(3)	1.10(3)	1.10(3)
**C12**	**H12**	1.075(8)	0.950(3)	1.04(4)	1.04(4)	1.03(4)	1.03(4)	1.03(4)	1.03(4)	1.03(4)	1.03(4)	1.03(4)	1.03(4)	1.03(4)	1.03(4)
**C13**	**H13**	1.073(8)	0.950(3)	1.03(3)	1.03(3)	1.03(3)	1.03(3)	1.03(3)	1.03(3)	1.03(3)	1.03(3)	1.03(3)	1.03(3)	1.03(3)	1.03(3)
**C14**	**H14**	1.103(9)	0.948(3)	1.11(5)	1.11(5)	1.11(5)	1.11(5)	1.11(5)	1.11(5)	1.11(5)	1.12(5)	1.11(5)	1.11(5)	1.12(5)	1.11(5)
**C16**	**H16A**	1.075(10)	0.982(4)	1.07(5)	1.07(5)	1.07(5)	1.07(5)	1.07(5)	1.07(5)	1.07(5)	1.07(5)	1.07(5)	1.07(5)	1.07(5)	1.07(5)
**C16**	**H16B**	1.083(8)	0.978(4)	1.13(6)	1.14(6)	1.13(6)	1.13(6)	1.14(6)	1.13(6)	1.13(6)	1.14(6)	1.13(6)	1.13(6)	1.14(6)	1.13(6)
**C16**	**H16C**	1.092(12)	0.980(4)	1.10(4)	1.11(4)	1.10(4)	1.10(4)	1.10(4)	1.10(4)	1.10(4)	1.10(4)	1.10(4)	1.10(4)	1.10(4)	1.10(4)
**C17**	**H17A**	1.082(10)	0.980(4)	1.06(5)	1.06(5)	1.06(5)	1.06(5)	1.06(5)	1.06(5)	1.06(5)	1.06(5)	1.06(5)	1.06(5)	1.06(5)	1.06(5)
**C17**	**H17B**	1.079(11)	0.981(4)	1.10(5)	1.10(5)	1.10(5)	1.10(5)	1.11(5)	1.11(5)	1.10(5)	1.10(5)	1.11(5)	1.10(5)	1.10(5)	1.11(5)
**C17**	**H17C**	1.092(10)	0.980(4)	1.01(6)	1.01(6)	1.01(6)	1.01(6)	1.01(6)	1.01(6)	1.01(6)	1.02(6)	1.01(6)	1.01(6)	1.02(6)	1.01(6)
**C18**	**H18A**	1.081(7)	0.979(3)	1.07(4)	1.07(4)	1.07(4)	1.06(4)	1.07(4)	1.06(4)	1.06(4)	1.07(4)	1.06(4)	1.06(4)	1.07(4)	1.06(4)

**C18**	**H18B**	1.082(10)	0.980(4)	1.18(5)	1.18(5)	1.18(5)	1.17(5)	1.18(5)	1.18(5)	1.17(5)	1.17(5)	1.18(5)	1.17(5)	1.17(5)	1.18(5)
**C18**	**H18C**	1.085(11)	0.980(4)	1.03(6)	1.03(6)	1.03(6)	1.03(6)	1.04(6)	1.03(6)	1.03(6)	1.03(6)	1.03(6)	1.03(6)	1.03(6)	1.03(6)
**C21**	**H21**	1.084(9)	0.950(3)	1.05(4)	1.05(4)	1.05(4)	1.05(4)	1.05(4)	1.05(4)	1.05(4)	1.05(4)	1.05(4)	1.05(4)	1.05(4)	1.05(4)
**C22**	**H22**	1.089(9)	0.949(3)	1.05(4)	1.06(4)	1.05(4)	1.05(4)	1.06(4)	1.05(4)	1.05(4)	1.05(4)	1.05(4)	1.05(4)	1.05(4)	1.05(4)
**C23**	**H23**	1.077(8)	0.951(3)	1.14(5)	1.15(5)	1.14(5)	1.14(5)	1.14(5)	1.14(5)	1.14(5)	1.14(5)	1.14(5)	1.14(5)	1.14(5)	1.14(5)
**C24**	**H24**	1.067(9)	0.949(3)	1.06(4)	1.06(4)	1.06(4)	1.06(4)	1.06(4)	1.06(4)	1.06(4)	1.06(4)	1.06(4)	1.06(4)	1.06(4)	1.06(4)
**C26**	**H26A**	1.095(12)	0.980(5)	1.08(6)	1.08(6)	1.07(6)	1.08(6)	1.08(6)	1.07(6)	1.08(6)	1.08(6)	1.08(6)	1.08(6)	1.08(6)	1.07(6)
**C26**	**H26B**	1.111(10)	0.981(4)	1.19(5)	1.20(5)	1.19(5)	1.19(5)	1.19(5)	1.19(5)	1.19(5)	1.19(5)	1.19(5)	1.19(5)	1.19(5)	1.19(5)
**C26**	**H26C**	1.093(10)	0.982(5)	1.05(5)	1.05(5)	1.05(5)	1.04(5)	1.05(5)	1.04(5)	1.04(5)	1.04(5)	1.04(5)	1.04(5)	1.04(5)	1.04(5)
**C27**	**H27A**	1.084(10)	0.980(4)	1.00(6)	1.00(6)	1.00(6)	0.99(6)	1.00(6)	1.00(6)	0.99(6)	0.99(6)	1.00(6)	0.99(6)	0.99(6)	1.00(6)
**C27**	**H27B**	1.100(11)	0.979(4)	1.09(5)	1.09(5)	1.09(5)	1.09(5)	1.09(5)	1.09(5)	1.09(5)	1.09(5)	1.09(5)	1.09(5)	1.09(5)	1.09(5)
**C27**	**H27C**	1.090(11)	0.981(4)	1.15(5)	1.15(5)	1.15(5)	1.15(6)	1.15(5)	1.15(6)	1.15(5)	1.15(5)	1.15(5)	1.15(5)	1.14(5)	1.15(5)
**C28**	**H28A**	1.079(10)	0.977(4)	1.10(6)	1.11(6)	1.11(6)	1.10(6)	1.10(6)	1.10(6)	1.10(6)	1.10(6)	1.10(6)	1.10(6)	1.10(6)	1.10(6)
**C28**	**H28B**	1.084(10)	0.980(5)	1.09(7)	1.10(7)	1.10(7)	1.10(7)	1.11(7)	1.11(7)	1.10(7)	1.10(7)	1.11(7)	1.10(7)	1.10(7)	1.10(7)
**C28**	**H28C**	1.076(12)	0.981(4)	1.13(6)	1.13(6)	1.13(6)	1.13(6)	1.13(6)	1.13(6)	1.13(6)	1.13(6)	1.13(6)	1.13(6)	1.12(6)	1.12(6)
**C52**	**H52**	1.091(8)	0.949(3)	1.11(4)	1.11(4)	1.11(4)	1.10(4)	1.11(4)	1.11(4)	1.10(4)	1.10(4)	1.11(4)	1.10(4)	1.10(4)	1.11(4)
**C54**	**H54**	1.088(7)	0.951(3)	1.14(4)	1.15(4)	1.14(4)	1.14(4)	1.14(4)	1.14(4)	1.14(4)	1.14(3)	1.14(4)	1.14(4)	1.14(3)	1.14(4)
**C56**	**H56**	1.074(7)	0.951(3)	1.16(4)	1.17(4)	1.17(4)	1.16(4)	1.17(4)	1.17(4)	1.16(4)	1.16(4)	1.17(4)	1.17(4)	1.16(4)	1.17(4)
**C62**	**H62**	1.086(8)	0.949(3)	1.16(4)	1.16(4)	1.16(4)	1.15(4)	1.16(4)	1.15(4)	1.16(4)	1.15(4)	1.15(4)	1.15(4)	1.15(4)	1.15(4)
**C64**	**H64**	1.074(8)	0.949(3)	1.11(4)	1.11(4)	1.11(4)	1.10(4)	1.11(4)	1.11(4)	1.11(4)	1.10(4)	1.11(4)	1.10(4)	1.10(4)	1.11(4)
**C66**	**H66**	1.095(9)	0.951(3)	1.11(4)	1.11(4)	1.11(4)	1.11(4)	1.11(4)	1.11(4)	1.11(4)	1.11(4)	1.11(4)	1.11(4)	1.11(4)	1.11(4)
**C72**	**H72**	1.082(9)	0.950(3)	1.06(3)	1.06(3)	1.06(3)	1.06(3)	1.06(3)	1.06(3)	1.06(3)	1.06(3)	1.06(3)	1.06(3)	1.06(3)	1.06(3)
**C74**	**H74**	1.082(8)	0.949(3)	1.05(4)	1.05(4)	1.05(4)	1.05(4)	1.05(4)	1.05(4)	1.05(4)	1.05(4)	1.04(4)	1.05(4)	1.05(4)	1.04(4)
**C76**	**H76**	1.084(9)	0.950(3)	1.06(4)	1.05(4)	1.05(4)	1.05(4)	1.05(4)	1.04(4)	1.05(4)	1.05(4)	1.04(4)	1.05(4)	1.05(4)	1.04(4)
**C82**	**H82**	1.098(8)	0.949(3)	1.07(4)	1.07(4)	1.07(4)	1.07(4)	1.07(4)	1.07(4)	1.07(4)	1.07(4)	1.07(4)	1.07(4)	1.07(4)	1.07(4)
**C84**	**H84**	1.080(7)	0.949(3)	1.02(4)	1.02(4)	1.02(4)	1.01(4)	1.01(4)	1.01(4)	1.01(4)	1.01(4)	1.01(4)	1.01(4)	1.01(4)	1.01(4)
**C86**	**H86**	1.098(9)	0.951(3)	1.08(4)	1.08(4)	1.08(4)	1.08(4)	1.08(4)	1.08(4)	1.08(4)	1.08(4)	1.08(4)	1.08(4)	1.08(4)	1.08(4)
**SiA**	**HA**	1.737(10)	1.62(4)	1.72(6)	1.72(6)	1.71(6)	1.72(6)	1.72(6)	1.72(6)	1.72(6)	1.72(6)	1.71(6)	1.72(6)	1.72(6)	1.71(6)
**Ru**	**HA**	1.600(8)	1.55(4)	1.58(6)	1.58(5)	1.59(5)	1.57(6)	1.57(6)	1.58(6)	1.54(6)	1.55(6)	1.56(6)	1.55(6)	1.56(6)	1.57(6)
**Ru**	**HB**	1.587(7)	1.47(4)	1.47(4)	1.48(4)	1.48(4)	1.46(5)	1.46(5)	1.48(4)	1.44(5)	1.44(5)	1.46(5)	1.45(5)	1.45(5)	1.47(4)

Table S15 X-H bond lengths (units: Å) obtained for NOBBOX with various experimental methods (neutron and X-ray) and refinement methods (IAM and HAR). HAR was performed with a cluster of charges and dipoles modeling crystal environment (DiSCaMB) and without a cluster (NoSpherA2). DFT calculations for HAR were performed with various functionals in the non-relativistic version (B3LYP, PBE and M06-2X) and with relativistic correction (B3LYP-DKH2, PBE-DKH2 and M06-2X-DKH2). Basis sets used: (a) non-relativistic refinements: cc-pVTZ-DK, (b) relativistic refinements: cc-pVTZ-DK.

				DiSCaMB-HAR						NoSpherA2-HAR					
**bond**		**neutron**	**IAM**	**B3LYP**	**PBE**	**M06-2X**	**B3LYP-DKH2**	**PBE-DKH2**	**M06-2X-DKH2**	**B3LYP**	**PBE**	**M06-2X**	**B3LYP-DKH2**	**PBE-DKH2**	**M06-2X-DKH2**
**C1**	**H1A**	1.089(3)	0.9790(14)	1.087(13)	1.091(13)	1.088(13)	1.083(13)	1.086(13)	1.083(13)	1.083(13)	0.9781(13)	1.083(13)	0.9780(13)	0.9782(13)	1.083(13)
**C1**	**H1B**	1.085(3)	0.9795(13)	1.065(13)	1.067(13)	1.064(13)	1.068(14)	1.070(14)	1.068(14)	1.070(14)	0.9812(12)	1.069(14)	0.9811(12)	0.9812(12)	1.067(14)
**C1**	**H1C**	1.090(3)	0.9814(13)	1.082(13)	1.085(13)	1.082(13)	1.078(13)	1.081(13)	1.078(14)	1.076(14)	0.9820(12)	1.077(14)	0.9819(12)	0.9820(12)	1.077(13)
**C3**	**H3A**	1.105(3)	0.9898(12)	1.122(11)	1.125(11)	1.123(11)	1.122(12)	1.125(12)	1.123(12)	1.124(12)	0.9919(11)	1.124(12)	0.9919(11)	0.9919(11)	1.122(12)
**C3**	**H3B**	1.092(2)	0.9920(12)	1.071(11)	1.071(11)	1.069(11)	1.068(11)	1.068(11)	1.066(11)	1.068(12)	0.9944(11)	1.066(11)	0.9945(11)	0.9944(11)	1.065(11)
**C4**	**H4A**	1.097(3)	0.9902(13)	1.073(12)	1.076(12)	1.073(12)	1.072(12)	1.075(12)	1.072(12)	1.073(12)	0.9921(12)	1.073(12)	0.9921(12)	0.9922(12)	1.071(12)
**C4**	**H4B**	1.092(2)	0.9888(12)	1.068(11)	1.070(11)	1.067(11)	1.063(12)	1.065(12)	1.063(12)	1.065(12)	0.9896(12)	1.064(12)	0.9894(11)	0.9896(12)	1.062(12)
**C5**	**H5A**	1.104(3)	0.9891(13)	1.094(12)	1.098(11)	1.095(12)	1.094(12)	1.098(12)	1.095(12)	1.096(12)	0.9899(12)	1.096(12)	0.9899(12)	0.9899(12)	1.095(12)
**C5**	**H5B**	1.098(3)	0.9897(14)	1.079(12)	1.081(12)	1.078(12)	1.075(13)	1.075(13)	1.073(13)	1.076(13)	0.9930(13)	1.073(13)	0.9929(13)	0.9930(13)	1.072(13)
**C7**	**H7A**	1.097(3)	0.9898(15)	1.075(13)	1.079(13)	1.075(13)	1.071(14)	1.073(14)	1.070(14)	1.072(14)	0.9884(14)	1.071(14)	0.9883(14)	0.9884(14)	1.070(14)
**C7**	**H7B**	1.105(3)	0.9890(16)	1.084(14)	1.087(14)	1.084(14)	1.086(14)	1.088(14)	1.086(15)	1.087(15)	0.9936(15)	1.087(15)	0.9938(15)	0.9937(15)	1.085(15)
**C8**	**H8A**	1.097(3)	0.9911(14)	1.071(12)	1.074(12)	1.072(12)	1.071(13)	1.073(13)	1.071(13)	1.070(13)	0.9934(13)	1.070(13)	0.9933(13)	0.9934(13)	1.070(13)
**C8**	**H8B**	1.090(2)	0.9885(13)	1.082(12)	1.085(12)	1.083(12)	1.081(12)	1.084(12)	1.082(12)	1.082(12)	0.9880(12)	1.083(12)	0.9878(12)	0.9880(12)	1.081(12)
**C9**	**H9A**	1.102(3)	0.9894(12)	1.092(11)	1.096(11)	1.094(11)	1.091(11)	1.095(11)	1.092(11)	1.091(11)	0.9905(11)	1.093(11)	0.9906(11)	0.9906(11)	1.092(11)
**C9**	**H9B**	1.096(2)	0.9889(13)	1.073(11)	1.075(11)	1.071(11)	1.072(11)	1.073(11)	1.070(11)	1.071(11)	0.9911(12)	1.070(11)	0.9910(12)	0.9911(12)	1.069(11)
**C10**	**H10A**	1.093(3)	0.9790(14)	1.072(14)	1.074(13)	1.072(14)	1.068(14)	1.071(14)	1.069(14)	1.068(14)	0.9797(13)	1.068(14)	0.9795(13)	0.9797(13)	1.068(14)
**C10**	**H10B**	1.087(3)	0.9807(13)	1.052(14)	1.056(14)	1.053(14)	1.053(15)	1.055(15)	1.053(15)	1.054(15)	0.9818(13)	1.054(15)	0.9818(13)	0.9818(13)	1.052(15)
**C10**	**H10C**	1.087(3)	0.9812(14)	1.083(15)	1.087(15)	1.083(15)	1.085(15)	1.088(15)	1.084(15)	1.087(15)	0.9811(14)	1.086(15)	0.9810(14)	0.9811(14)	1.084(15)
**C11**	**H11A**	1.104(3)	0.9901(14)	1.080(13)	1.083(13)	1.080(13)	1.078(13)	1.080(13)	1.077(13)	1.078(13)	0.9942(13)	1.078(13)	0.9943(13)	0.9943(13)	1.077(13)
**C11**	**H11B**	1.094(3)	0.9903(12)	1.092(12)	1.095(12)	1.093(12)	1.091(12)	1.094(12)	1.091(12)	1.092(12)	0.9903(12)	1.092(12)	0.9903(12)	0.9903(12)	1.091(12)
**C12**	**H12A**	1.094(2)	0.9876(13)	1.104(12)	1.108(12)	1.106(12)	1.103(13)	1.106(13)	1.104(13)	1.104(13)	0.9904(12)	1.105(13)	0.9904(12)	0.9905(12)	1.104(13)
**C12**	**H12B**	1.097(3)	0.9894(14)	1.082(13)	1.084(12)	1.082(13)	1.084(13)	1.086(13)	1.084(13)	1.084(13)	0.9909(13)	1.085(13)	0.9910(13)	0.9909(13)	1.083(13)
**C13**	**H13A**	1.098(3)	0.9904(14)	1.103(13)	1.106(13)	1.103(13)	1.101(13)	1.103(13)	1.101(13)	1.101(13)	0.9927(13)	1.101(13)	0.9927(13)	0.9927(13)	1.101(13)
**C13**	**H13B**	1.103(3)	0.9894(14)	1.086(14)	1.089(14)	1.086(14)	1.084(14)	1.087(14)	1.085(14)	1.085(14)	0.9913(14)	1.085(14)	0.9913(14)	0.9913(14)	1.084(14)
**C15**	**H15A**	1.102(3)	0.9900(13)	1.089(12)	1.093(12)	1.090(12)	1.086(12)	1.090(12)	1.087(12)	1.086(12)	0.9906(12)	1.087(12)	0.9906(12)	0.9907(12)	1.087(12)
**C15**	**H15B**	1.102(3)	0.9892(14)	1.104(12)	1.107(12)	1.104(12)	1.098(13)	1.100(13)	1.097(13)	1.099(13)	0.9920(13)	1.098(13)	0.9921(13)	0.9920(13)	1.097(13)

**C16**	**H16A**	1.096(3)	0.9915(14)	1.074(13)	1.075(12)	1.072(13)	1.073(13)	1.075(13)	1.072(13)	1.073(13)	0.9936(13)	1.072(13)	0.9936(13)	0.9937(13)	1.071(13)
**C16**	**H16B**	1.099(2)	0.9890(12)	1.083(12)	1.086(12)	1.084(12)	1.081(12)	1.082(12)	1.081(12)	1.083(13)	0.9895(11)	1.082(12)	0.9893(11)	0.9895(11)	1.080(12)
**C17**	**H17A**	1.102(3)	0.9889(13)	1.105(11)	1.108(11)	1.105(11)	1.106(12)	1.108(12)	1.105(12)	1.107(12)	0.9911(12)	1.106(12)	0.9913(12)	0.9912(12)	1.105(12)
**C17**	**H17B**	1.093(2)	0.9905(13)	1.083(11)	1.085(11)	1.081(11)	1.082(12)	1.084(11)	1.081(12)	1.084(12)	0.9923(12)	1.082(12)	0.9923(12)	0.9923(12)	1.080(12)
**C19**	**H19A**	1.084(3)	0.9806(14)	1.059(13)	1.061(13)	1.058(13)	1.051(14)	1.054(14)	1.051(14)	1.049(14)	0.9819(13)	1.050(14)	0.9819(13)	0.9820(13)	1.050(14)
**C19**	**H19B**	1.083(3)	0.9800(12)	1.068(12)	1.071(12)	1.069(12)	1.072(13)	1.074(13)	1.072(13)	1.074(13)	0.9791(12)	1.073(13)	0.9788(12)	0.9790(12)	1.072(13)
**C19**	**H19C**	1.091(3)	0.9781(13)	1.086(12)	1.089(12)	1.087(12)	1.083(13)	1.085(13)	1.083(13)	1.084(13)	0.9784(12)	1.084(13)	0.9783(12)	0.9784(12)	1.083(13)
**C20**	**H20A**	1.087(3)	0.9791(13)	1.091(12)	1.094(12)	1.092(12)	1.092(13)	1.095(13)	1.093(13)	1.094(13)	0.9789(12)	1.094(13)	0.9788(12)	0.9789(12)	1.092(13)
**C20**	**H20B**	1.092(3)	0.9806(13)	1.089(13)	1.091(13)	1.089(13)	1.092(14)	1.094(14)	1.093(14)	1.094(14)	0.9797(12)	1.093(14)	0.9796(12)	0.9797(12)	1.092(14)
**C20**	**H20C**	1.083(3)	0.9788(14)	1.075(14)	1.078(14)	1.076(14)	1.073(14)	1.076(14)	1.074(14)	1.074(15)	0.9789(13)	1.074(14)	0.9787(13)	0.9789(13)	1.073(14)
**C21**	**H21A**	1.087(3)	0.9795(13)	1.067(12)	1.070(12)	1.067(12)	1.062(13)	1.065(13)	1.062(13)	1.062(13)	0.9806(13)	1.062(13)	0.9805(12)	0.9806(12)	1.062(13)
**C21**	**H21B**	1.085(3)	0.9810(13)	1.060(13)	1.063(13)	1.060(13)	1.058(13)	1.061(13)	1.058(13)	1.057(13)	0.9812(12)	1.058(13)	0.9812(12)	0.9812(12)	1.058(13)
**C21**	**H21C**	1.085(3)	0.9808(12)	1.083(12)	1.086(12)	1.084(12)	1.085(12)	1.088(12)	1.086(12)	1.084(13)	0.9792(11)	1.085(13)	0.9791(11)	0.9792(11)	1.085(12)
**C22**	**H22A**	1.092(3)	0.9896(13)	1.111(12)	1.114(12)	1.111(12)	1.112(13)	1.115(12)	1.112(13)	1.113(13)	0.9916(12)	1.113(13)	0.9916(12)	0.9916(12)	1.112(13)
**C22**	**H22B**	1.105(3)	0.9902(12)	1.088(12)	1.090(12)	1.087(12)	1.087(12)	1.089(12)	1.087(12)	1.089(13)	0.9925(11)	1.088(13)	0.9925(11)	0.9925(11)	1.086(12)
**C23**	**H23A**	1.095(3)	0.9894(12)	1.078(12)	1.081(12)	1.079(12)	1.077(13)	1.080(12)	1.078(13)	1.077(13)	0.9890(12)	1.078(13)	0.9890(11)	0.9891(11)	1.077(13)
**C23**	**H23B**	1.103(3)	0.9897(13)	1.092(11)	1.095(11)	1.093(11)	1.089(12)	1.092(12)	1.090(12)	1.091(12)	0.9908(12)	1.091(12)	0.9906(12)	0.9908(12)	1.090(12)
**C24**	**H24A**	1.105(3)	0.9911(14)	1.085(12)	1.088(12)	1.086(12)	1.085(12)	1.088(12)	1.086(12)	1.086(12)	0.9919(13)	1.086(12)	0.9919(12)	0.9920(12)	1.085(12)
**C24**	**H24B**	1.098(2)	0.9892(14)	1.082(13)	1.084(13)	1.081(13)	1.075(13)	1.077(13)	1.075(13)	1.076(14)	0.9908(13)	1.076(13)	0.9908(13)	0.9908(13)	1.074(13)
**C26**	**H26A**	1.094(2)	0.9901(13)	1.096(12)	1.099(12)	1.097(12)	1.091(12)	1.093(12)	1.092(13)	1.092(13)	0.9917(12)	1.092(13)	0.9917(12)	0.9917(12)	1.091(13)
**C26**	**H26B**	1.102(3)	0.9901(12)	1.092(11)	1.095(11)	1.092(12)	1.094(12)	1.096(12)	1.095(12)	1.095(12)	0.9913(11)	1.095(12)	0.9912(11)	0.9913(11)	1.094(12)
**C27**	**H27A**	1.096(2)	0.9883(13)	1.072(12)	1.076(12)	1.073(12)	1.064(13)	1.067(13)	1.065(13)	1.065(13)	0.9885(12)	1.066(13)	0.9883(12)	0.9885(12)	1.064(13)
**C27**	**H27B**	1.095(3)	0.9905(13)	1.082(13)	1.083(13)	1.080(13)	1.084(13)	1.086(13)	1.083(13)	1.085(13)	0.9931(12)	1.084(13)	0.9931(12)	0.9931(12)	1.082(13)
**C28**	**H28A**	1.097(2)	0.9898(12)	1.083(11)	1.085(11)	1.082(11)	1.084(12)	1.086(12)	1.083(12)	1.086(12)	0.9927(12)	1.084(12)	0.9926(11)	0.9927(11)	1.082(12)
**C28**	**H28B**	1.105(3)	0.9888(12)	1.104(11)	1.108(11)	1.105(11)	1.100(12)	1.104(12)	1.102(12)	1.102(12)	0.9898(11)	1.103(12)	0.9898(11)	0.9899(11)	1.101(12)
**C30**	**H30A**	1.092(3)	0.9791(14)	1.086(13)	1.090(12)	1.086(13)	1.087(13)	1.089(13)	1.086(13)	1.089(13)	0.9789(13)	1.088(13)	0.9787(13)	0.9788(13)	1.086(13)
**C30**	**H30B**	1.086(3)	0.9801(15)	1.070(13)	1.074(13)	1.071(13)	1.073(14)	1.077(14)	1.074(14)	1.075(14)	0.9795(14)	1.075(14)	0.9793(14)	0.9795(14)	1.074(14)
**C30**	**H30C**	1.084(3)	0.9797(15)	1.053(14)	1.055(14)	1.053(14)	1.050(15)	1.052(14)	1.049(15)	1.049(15)	0.9822(14)	1.049(15)	0.9823(14)	0.9823(14)	1.048(15)
**SiA**	**HA**	1.874(3)	1.82(2)	1.868(13)	1.868(12)	1.868(13)	1.868(13)	1.868(13)	1.868(13)	1.868(13)	1.853(17)	1.867(13)	1.855(17)	1.854(17)	1.867(13)
**Ru**	**HA**	1.598(3)	1.55(2)	1.593(11)	1.599(11)	1.602(11)	1.595(12)	1.601(12)	1.606(12)	1.583(13)	1.558(16)	1.594(13)	1.564(15)	1.563(15)	1.599(12)

Table S16 X-H bond lengths (units: Å) obtained for SITKUB with various experimental methods (neutron and X-ray) and refinement methods (IAM and HAR). HAR was performed with a cluster of charges and dipoles modeling crystal environment (DiSCaMB) and without a cluster (NoSpherA2). DFT calculations for HAR were performed with various functionals in the non-relativistic version (B3LYP, PBE and M06-2X) and with relativistic correction (B3LYP-DKH2, PBE-DKH2 and M06-2X-DKH2). Basis sets used: (a) non-relativistic refinements: cc-pVTZ-DK, (b) relativistic refinements: cc-pVTZ-DK.

				DiSCaMB-HAR						NoSpherA2-HAR					
**bond**		**neutron**	**IAM**	**B3LYP**	**PBE**	**M06-2X**	**B3LYP-DKH2**	**PBE-DKH2**	**M06-2X-DKH2**	**B3LYP**	**PBE**	**M06-2X**	**B3LYP-DKH2**	**PBE-DKH2**	**M06-2X-DKH2**
**Rh**	**H**	1.531(11)	1.40(2)	1.473(15)	1.478(15)	1.489(15)	1.465(17)	1.471(16)	1.485(16)	1.497(16)	1.463(17)	1.511(15)	1.465(17)	1.465(17)	1.484(16)
**C3**	**H3**	1.080(16)	0.9488(NA)	1.104(19)	1.105(19)	1.102(19)	1.103(19)	1.10(2)	1.10(2)	1.11(2)	1.104(19)	1.11(2)	1.108(19)	1.107(19)	1.107(19)
**C4**	**H4**	1.081(12)	0.9485(NA)	1.073(19)	1.076(19)	1.073(19)	1.070(19)	1.074(19)	1.07(2)	1.07(2)	1.073(19)	1.07(2)	1.073(19)	1.074(19)	1.076(19)
**C5**	**H5**	1.098(11)	0.9482(NA)	1.071(18)	1.072(18)	1.070(18)	1.068(18)	1.069(18)	1.067(18)	1.076(19)	1.066(18)	1.074(19)	1.072(18)	1.071(18)	1.070(18)
**C6**	**H6**	1.071(14)	0.9474(NA)	1.090(16)	1.090(16)	1.088(17)	1.083(17)	1.084(17)	1.081(17)	1.091(17)	1.082(17)	1.086(18)	1.088(17)	1.086(17)	1.086(17)
**C7**	**H7**	1.100(9)	0.9988(NA)	1.112(17)	1.113(17)	1.113(17)	1.112(18)	1.113(18)	1.113(18)	1.113(18)	1.110(18)	1.114(18)	1.114(18)	1.113(17)	1.115(17)
**C8**	**H8A**	1.079(10)	0.964(12)	1.06(2)	1.06(2)	1.06(2)	1.06(2)	1.06(2)	1.06(2)	1.06(2)	1.06(2)	1.06(2)	1.06(2)	1.06(2)	1.06(2)
**C8**	**H8B**	1.054(16)	0.963(12)	1.127(19)	1.129(19)	1.126(19)	1.126(19)	1.128(19)	1.125(19)	1.13(2)	1.128(19)	1.12(2)	1.130(19)	1.130(19)	1.128(19)
**C8**	**H8C**	1.072(16)	0.965(12)	1.081(17)	1.084(17)	1.081(17)	1.082(18)	1.086(18)	1.082(18)	1.091(18)	1.082(18)	1.093(18)	1.084(18)	1.085(17)	1.084(18)
**C9**	**H9A**	1.059(18)	0.955(12)	1.101(17)	1.103(17)	1.101(17)	1.101(18)	1.103(18)	1.102(18)	1.105(18)	1.101(18)	1.104(18)	1.104(18)	1.103(18)	1.105(18)
**C9**	**H9B**	1.087(11)	0.955(12)	1.06(2)	1.07(2)	1.06(2)	1.06(2)	1.06(2)	1.06(2)	1.07(2)	1.07(2)	1.07(2)	1.06(2)	1.06(2)	1.06(2)
**C9**	**H9C**	1.085(10)	0.955(12)	1.06(2)	1.06(2)	1.05(2)	1.05(2)	1.05(2)	1.05(2)	1.06(2)	1.05(2)	1.05(2)	1.05(2)	1.05(2)	1.05(2)
**C10**	**H10**	1.085(13)	0.9986(NA)	1.106(17)	1.106(17)	1.105(17)	1.106(17)	1.107(18)	1.106(18)	1.111(18)	1.102(17)	1.110(18)	1.109(17)	1.107(17)	1.111(17)
**C11**	**H11A**	1.083(13)	0.965(13)	1.082(19)	1.086(19)	1.084(19)	1.08(2)	1.08(2)	1.08(2)	1.08(2)	1.08(2)	1.08(2)	1.08(2)	1.08(2)	1.08(2)
**C11**	**H11B**	1.083(11)	0.965(13)	1.08(2)	1.08(2)	1.08(2)	1.08(2)	1.08(2)	1.08(2)	1.08(2)	1.08(2)	1.08(2)	1.08(2)	1.08(2)	1.08(2)
**C11**	**H11C**	1.088(15)	0.966(13)	1.096(19)	1.10(2)	1.09(2)	1.10(2)	1.10(2)	1.10(2)	1.11(2)	1.10(2)	1.11(2)	1.10(2)	1.10(2)	1.10(2)
**C12**	**H12A**	1.071(13)	0.943(11)	1.07(2)	1.07(2)	1.06(2)	1.06(2)	1.07(2)	1.06(2)	1.07(2)	1.06(2)	1.07(2)	1.07(2)	1.07(2)	1.07(2)
**C12**	**H12B**	1.072(14)	0.943(11)	1.079(16)	1.081(16)	1.077(16)	1.081(16)	1.082(17)	1.079(17)	1.080(17)	1.081(16)	1.078(17)	1.083(16)	1.082(16)	1.081(16)
**C12**	**H12C**	1.092(13)	0.943(11)	1.081(18)	1.085(18)	1.081(18)	1.078(18)	1.082(18)	1.079(18)	1.079(19)	1.080(18)	1.078(19)	1.081(18)	1.083(18)	1.082(18)
**C13**	**H13**	1.089(14)	0.9989(NA)	1.094(17)	1.096(17)	1.094(17)	1.094(17)	1.096(17)	1.095(17)	1.099(17)	1.095(17)	1.101(17)	1.096(17)	1.096(17)	1.097(17)
**C14**	**H14A**	1.095(11)	0.974(13)	1.07(2)	1.07(2)	1.07(2)	1.07(2)	1.07(2)	1.07(2)	1.07(2)	1.07(2)	1.07(2)	1.07(2)	1.07(2)	1.07(2)
**C14**	**H14B**	1.073(14)	0.975(13)	1.10(2)	1.10(2)	1.09(2)	1.09(2)	1.09(2)	1.09(2)	1.10(2)	1.09(2)	1.10(2)	1.10(2)	1.10(2)	1.09(2)
**C14**	**H14C**	1.082(16)	0.976(13)	1.09(2)	1.09(2)	1.09(2)	1.09(2)	1.09(2)	1.09(2)	1.10(2)	1.09(2)	1.10(2)	1.09(2)	1.09(2)	1.09(2)
**C15**	**H15A**	1.045(19)	0.975(14)	1.09(2)	1.10(2)	1.09(2)	1.09(2)	1.10(2)	1.10(2)	1.10(2)	1.10(2)	1.10(2)	1.10(2)	1.10(2)	1.10(2)
**C15**	**H15B**	1.095(13)	0.975(14)	1.08(2)	1.08(2)	1.08(2)	1.08(2)	1.08(2)	1.08(2)	1.08(2)	1.08(2)	1.08(2)	1.08(2)	1.08(2)	1.08(2)
**C15**	**H15C**	1.090(11)	0.974(14)	1.10(2)	1.10(2)	1.10(2)	1.10(2)	1.10(2)	1.10(2)	1.10(2)	1.10(2)	1.10(2)	1.10(2)	1.10(2)	1.10(2)

**C16**	**H16**	1.094(11)	0.9983(NA)	1.092(16)	1.092(17)	1.090(17)	1.082(17)	1.082(17)	1.080(17)	1.092(17)	1.078(17)	1.092(17)	1.086(17)	1.083(17)	1.084(17)
**C17**	**H17A**	1.096(12)	0.968(13)	1.079(19)	1.08(2)	1.07(2)	1.08(2)	1.08(2)	1.08(2)	1.08(2)	1.085(19)	1.08(2)	1.08(2)	1.08(2)	1.08(2)
**C17**	**H17B**	1.096(14)	0.969(13)	1.08(2)	1.09(2)	1.08(2)	1.08(2)	1.09(2)	1.08(2)	1.10(2)	1.09(2)	1.10(2)	1.09(2)	1.09(2)	1.09(2)
**C17**	**H17C**	1.088(14)	0.968(13)	1.093(16)	1.097(16)	1.092(16)	1.093(17)	1.097(17)	1.093(17)	1.096(17)	1.09(2)	1.099(17)	1.094(17)	1.094(16)	1.094(16)
**C18**	**H18A**	1.077(13)	0.976(13)	1.085(18)	1.090(18)	1.088(18)	1.084(19)	1.088(19)	1.087(19)	1.089(19)	1.085(18)	1.095(19)	1.085(18)	1.086(18)	1.087(18)
**C18**	**H18B**	1.081(13)	0.976(13)	1.08(2)	1.08(2)	1.08(2)	1.08(2)	1.08(2)	1.08(2)	1.09(2)	1.08(2)	1.09(2)	1.08(2)	1.08(2)	1.08(2)
**C18**	**H18C**	1.085(13)	0.975(13)	1.07(2)	1.08(2)	1.07(2)	1.08(2)	1.08(2)	1.08(2)	1.09(2)	1.08(2)	1.08(2)	1.08(2)	1.08(2)	1.08(2)
**C19**	**H19**	1.103(13)	0.9982(NA)	1.053(18)	1.053(18)	1.050(18)	1.051(18)	1.052(19)	1.048(19)	1.067(18)	1.051(18)	1.068(19)	1.056(18)	1.055(18)	1.053(18)
**C20**	**H20A**	1.087(12)	0.957(14)	1.08(2)	1.08(2)	1.08(2)	1.08(2)	1.08(2)	1.08(2)	1.09(2)	1.08(2)	1.09(2)	1.08(2)	1.08(2)	1.08(2)
**C20**	**H20B**	1.078(14)	0.957(14)	1.10(2)	1.10(2)	1.10(2)	1.10(2)	1.10(2)	1.10(2)	1.11(2)	1.11(2)	1.11(2)	1.11(2)	1.11(2)	1.11(2)
**C20**	**H20C**	1.091(15)	0.956(14)	1.07(3)	1.07(3)	1.06(3)	1.07(3)	1.07(3)	1.06(3)	1.07(3)	1.07(3)	1.07(3)	1.07(3)	1.08(3)	1.07(3)
**C21**	**H21A**	1.049(17)	0.974(14)	1.07(2)	1.08(2)	1.07(3)	1.07(3)	1.07(3)	1.07(3)	1.08(3)	1.07(3)	1.08(3)	1.07(3)	1.07(3)	1.07(3)
**C21**	**H21B**	1.071(16)	0.973(14)	1.05(3)	1.05(3)	1.05(3)	1.04(3)	1.05(3)	1.04(3)	1.05(3)	1.04(3)	1.05(3)	1.05(3)	1.05(3)	1.05(3)
**C21**	**H21C**	1.077(15)	0.975(14)	1.103(19)	1.107(19)	1.105(19)	1.102(19)	1.106(19)	1.104(19)	1.10(2)	1.104(19)	1.11(2)	1.102(19)	1.103(19)	1.104(19)
**C22**	**H22**	1.077(12)	0.9984(NA)	1.094(16)	1.097(16)	1.095(17)	1.095(17)	1.097(17)	1.095(17)	1.096(17)	1.091(17)	1.097(17)	1.096(17)	1.095(16)	1.096(16)
**C23**	**H23A**	1.104(13)	0.948(13)	1.07(2)	1.07(2)	1.07(2)	1.07(2)	1.07(2)	1.07(2)	1.08(2)	1.07(2)	1.08(2)	1.07(2)	1.07(2)	1.07(2)
**C23**	**H23B**	1.083(15)	0.948(13)	1.05(2)	1.06(2)	1.05(2)	1.05(2)	1.06(2)	1.05(2)	1.05(2)	1.05(2)	1.05(2)	1.06(2)	1.05(2)	1.06(2)
**C23**	**H23C**	1.073(12)	0.948(13)	1.078(19)	1.082(19)	1.078(19)	1.08(2)	1.08(2)	1.08(2)	1.09(2)	1.08(2)	1.09(2)	1.081(19)	1.084(19)	1.080(19)
**C24**	**H24A**	1.093(13)	0.959(12)	1.107(18)	1.109(18)	1.108(18)	1.109(19)	1.112(19)	1.110(19)	1.114(19)	1.110(19)	1.115(19)	1.110(19)	1.110(18)	1.110(18)
**C24**	**H24B**	1.081(13)	0.959(12)	1.06(2)	1.07(2)	1.06(2)	1.06(2)	1.06(2)	1.06(2)	1.07(2)	1.06(2)	1.07(2)	1.06(2)	1.06(2)	1.06(2)
**C24**	**H24C**	1.076(14)	0.959(12)	1.07(2)	1.07(2)	1.07(2)	1.07(2)	1.07(2)	1.07(2)	1.08(2)	1.07(2)	1.08(2)	1.07(2)	1.07(2)	1.07(2)

Table S17 X-H bond lengths (units: Å) obtained for SITKUB with various experimental methods (neutron and X-ray) and refinement methods (IAM and HAR). HAR was performed with a cluster of charges and dipoles modeling crystal environment (DiSCaMB) and without a cluster (NoSpherA2). DFT calculations for HAR were performed with various functionals in the non-relativistic version (B3LYP, PBE and M06-2X) and with relativistic correction (B3LYP-DKH2, PBE-DKH2 and M06-2X-DKH2). Basis sets used: (a) non-relativistic refinements: jorge-DZP, (b) relativistic refinements: jorge-DZP-DKH.

				DiSCaMB-HAR						NoSpherA2-HAR					
**bond**		**neutron**	**IAM**	**B3LYP**	**PBE**	**M06-2X**	**B3LYP-DKH2**	**PBE-DKH2**	**M06-2X-DKH2**	**B3LYP**	**PBE**	**M06-2X**	**B3LYP-DKH2**	**PBE-DKH2**	**M06-2X-DKH2**
**Rh**	**H**	1.531(11)	1.40(2)	1.534(15)	1.546(14)	1.553(15)	1.537(14)	1.546(14)	1.556(13)	1.52(2)	1.523(19)	1.539(19)	1.514(19)	1.515(19)	1.514(19)
**C3**	**H3**	1.080(16)	0.9488(NA)	1.117(16)	1.120(16)	1.119(16)	1.112(16)	1.119(16)	1.118(16)	1.12(2)	1.12(2)	1.12(2)	1.12(2)	1.12(2)	1.12(2)
**C4**	**H4**	1.081(12)	0.9485(NA)	1.068(15)	1.072(15)	1.071(15)	1.064(15)	1.070(15)	1.069(15)	1.08(2)	1.08(2)	1.08(2)	1.08(2)	1.08(2)	1.08(2)
**C5**	**H5**	1.098(11)	0.9482(NA)	1.079(16)	1.080(16)	1.078(16)	1.076(16)	1.077(16)	1.075(16)	1.083(19)	1.082(19)	1.083(19)	1.082(19)	1.081(19)	1.082(19)
**C6**	**H6**	1.071(14)	0.9474(NA)	1.123(14)	1.123(14)	1.121(14)	1.121(14)	1.119(14)	1.117(14)	1.105(18)	1.104(18)	1.106(18)	1.104(18)	1.102(18)	1.104(18)
**C7**	**H7**	1.100(9)	0.9988(NA)	1.117(14)	1.121(14)	1.121(14)	1.115(14)	1.121(14)	1.120(14)	1.121(19)	1.120(19)	1.122(19)	1.120(19)	1.119(19)	1.120(19)
**C8**	**H8A**	1.079(10)	0.964(12)	1.073(19)	1.076(18)	1.072(19)	1.072(19)	1.075(19)	1.071(19)	1.07(2)	1.07(2)	1.07(2)	1.06(2)	1.07(2)	1.06(2)
**C8**	**H8B**	1.054(16)	0.963(12)	1.139(16)	1.143(15)	1.141(15)	1.140(16)	1.143(15)	1.141(15)	1.14(2)	1.14(2)	1.14(2)	1.14(2)	1.14(2)	1.14(2)
**C8**	**H8C**	1.072(16)	0.965(12)	1.093(15)	1.094(15)	1.093(15)	1.099(15)	1.098(15)	1.097(15)	1.09(2)	1.09(2)	1.09(2)	1.09(2)	1.09(2)	1.09(2)
**C9**	**H9A**	1.059(18)	0.955(12)	1.101(17)	1.106(17)	1.105(17)	1.100(17)	1.106(17)	1.105(17)	1.12(2)	1.12(2)	1.12(2)	1.12(2)	1.12(2)	1.12(2)
**C9**	**H9B**	1.087(11)	0.955(12)	1.084(17)	1.085(17)	1.081(17)	1.080(17)	1.083(17)	1.079(17)	1.07(2)	1.08(2)	1.07(2)	1.07(2)	1.07(2)	1.07(2)
**C9**	**H9C**	1.085(10)	0.955(12)	1.095(17)	1.095(17)	1.092(17)	1.092(17)	1.096(17)	1.093(17)	1.07(2)	1.07(2)	1.07(2)	1.07(2)	1.07(2)	1.07(2)
**C10**	**H10**	1.085(13)	0.9986(NA)	1.119(15)	1.119(13)	1.118(13)	1.122(15)	1.120(13)	1.120(13)	1.124(19)	1.122(19)	1.125(19)	1.122(19)	1.120(19)	1.122(19)
**C11**	**H11A**	1.083(13)	0.965(13)	1.097(16)	1.101(16)	1.101(16)	1.091(16)	1.097(16)	1.096(16)	1.09(2)	1.09(2)	1.09(2)	1.09(2)	1.09(2)	1.09(2)
**C11**	**H11B**	1.083(11)	0.965(13)	1.105(17)	1.108(17)	1.104(17)	1.101(17)	1.104(16)	1.101(17)	1.10(2)	1.10(2)	1.10(2)	1.10(2)	1.10(2)	1.10(2)
**C11**	**H11C**	1.088(15)	0.966(13)	1.117(17)	1.116(16)	1.112(17)	1.123(17)	1.122(16)	1.119(17)	1.12(2)	1.12(2)	1.12(2)	1.12(2)	1.12(2)	1.12(2)
**C12**	**H12A**	1.071(13)	0.943(11)	1.080(16)	1.085(16)	1.081(16)	1.087(16)	1.086(16)	1.083(16)	1.10(2)	1.10(2)	1.10(2)	1.10(2)	1.10(2)	1.10(2)
**C12**	**H12B**	1.072(14)	0.943(11)	1.099(14)	1.098(14)	1.095(14)	1.094(16)	1.100(14)	1.097(14)	1.093(18)	1.092(18)	1.093(18)	1.092(18)	1.091(18)	1.092(18)
**C12**	**H12C**	1.092(13)	0.943(11)	1.111(15)	1.114(15)	1.111(15)	1.109(15)	1.110(15)	1.108(15)	1.10(2)	1.10(2)	1.10(2)	1.10(2)	1.10(2)	1.10(2)
**C13**	**H13**	1.089(14)	0.9989(NA)	1.109(16)	1.112(15)	1.111(15)	1.111(16)	1.112(15)	1.111(15)	1.108(18)	1.108(18)	1.110(18)	1.108(18)	1.108(18)	1.108(18)
**C14**	**H14A**	1.095(11)	0.974(13)	1.078(18)	1.079(18)	1.078(18)	1.079(18)	1.079(18)	1.078(18)	1.09(2)	1.09(2)	1.09(2)	1.08(2)	1.08(2)	1.08(2)
**C14**	**H14B**	1.073(14)	0.975(13)	1.095(17)	1.097(17)	1.094(17)	1.094(17)	1.096(16)	1.093(17)	1.11(2)	1.11(2)	1.11(2)	1.11(2)	1.11(2)	1.11(2)
**C14**	**H14C**	1.082(16)	0.976(13)	1.106(18)	1.113(18)	1.112(18)	1.108(18)	1.116(18)	1.115(18)	1.09(2)	1.09(2)	1.09(2)	1.09(2)	1.09(2)	1.09(2)
**C15**	**H15A**	1.045(19)	0.975(14)	1.099(17)	1.102(17)	1.100(17)	1.099(17)	1.102(17)	1.101(17)	1.12(3)	1.12(3)	1.12(3)	1.12(3)	1.12(3)	1.12(3)
**C15**	**H15B**	1.095(13)	0.975(14)	1.089(17)	1.092(17)	1.088(17)	1.087(17)	1.091(17)	1.086(17)	1.08(2)	1.08(2)	1.08(2)	1.08(2)	1.08(2)	1.08(2)
**C15**	**H15C**	1.090(11)	0.974(14)	1.106(17)	1.108(17)	1.107(17)	1.103(17)	1.106(17)	1.105(17)	1.12(2)	1.12(2)	1.12(2)	1.11(2)	1.11(2)	1.11(2)

**C16**	**H16**	1.094(11)	0.9983(NA)	1.120(14)	1.118(14)	1.118(14)	1.109(14)	1.111(14)	1.109(14)	1.104(18)	1.102(18)	1.103(18)	1.102(18)	1.100(18)	1.102(18)
**C17**	**H17A**	1.096(12)	0.968(13)	1.115(18)	1.116(18)	1.112(18)	1.115(18)	1.117(18)	1.113(18)	1.11(2)	1.11(2)	1.11(2)	1.11(2)	1.11(2)	1.11(2)
**C17**	**H17B**	1.096(14)	0.969(13)	1.082(17)	1.086(17)	1.083(17)	1.080(17)	1.086(17)	1.083(17)	1.11(2)	1.11(2)	1.11(2)	1.10(2)	1.11(2)	1.10(2)
**C17**	**H17C**	1.088(14)	0.968(13)	1.115(16)	1.117(15)	1.115(15)	1.111(15)	1.118(15)	1.115(15)	1.10(3)	1.10(3)	1.10(3)	1.10(3)	1.10(3)	1.10(3)
**C18**	**H18A**	1.077(13)	0.976(13)	1.101(16)	1.103(15)	1.102(15)	1.101(16)	1.102(15)	1.102(15)	1.09(2)	1.09(2)	1.09(2)	1.09(2)	1.09(2)	1.09(2)
**C18**	**H18B**	1.081(13)	0.976(13)	1.094(19)	1.095(19)	1.094(19)	1.089(19)	1.095(19)	1.094(19)	1.11(2)	1.11(2)	1.11(2)	1.11(2)	1.11(2)	1.11(2)
**C18**	**H18C**	1.085(13)	0.975(13)	1.081(18)	1.083(17)	1.079(17)	1.085(18)	1.086(17)	1.083(17)	1.09(2)	1.10(2)	1.09(2)	1.09(2)	1.09(2)	1.09(2)
**C19**	**H19**	1.103(13)	0.9982(NA)	1.096(16)	1.096(16)	1.093(16)	1.096(16)	1.096(16)	1.094(16)	1.08(2)	1.08(2)	1.08(2)	1.08(2)	1.078(19)	1.08(2)
**C20**	**H20A**	1.087(12)	0.957(14)	1.086(16)	1.090(16)	1.089(16)	1.081(16)	1.085(16)	1.083(16)	1.09(2)	1.09(2)	1.09(2)	1.09(2)	1.09(2)	1.09(2)
**C20**	**H20B**	1.078(14)	0.957(14)	1.130(19)	1.133(19)	1.131(19)	1.135(19)	1.139(19)	1.136(19)	1.13(3)	1.13(2)	1.13(3)	1.13(2)	1.12(2)	1.13(2)
**C20**	**H20C**	1.091(15)	0.956(14)	1.11(2)	1.11(2)	1.11(2)	1.11(2)	1.11(2)	1.11(2)	1.10(3)	1.10(3)	1.10(3)	1.10(3)	1.10(3)	1.10(3)
**C21**	**H21A**	1.049(17)	0.974(14)	1.08(2)	1.08(2)	1.08(2)	1.08(2)	1.08(2)	1.08(2)	1.08(3)	1.08(3)	1.08(3)	1.08(3)	1.08(3)	1.08(3)
**C21**	**H21B**	1.071(16)	0.973(14)	1.09(2)	1.09(2)	1.09(2)	1.08(2)	1.09(2)	1.09(2)	1.07(3)	1.08(3)	1.08(3)	1.07(3)	1.07(3)	1.07(3)
**C21**	**H21C**	1.077(15)	0.975(14)	1.112(16)	1.114(16)	1.112(16)	1.114(16)	1.115(16)	1.114(16)	1.10(2)	1.10(2)	1.11(2)	1.10(2)	1.10(2)	1.10(2)
**C22**	**H22**	1.077(12)	0.9984(NA)	1.113(13)	1.117(13)	1.116(13)	1.116(15)	1.118(13)	1.118(13)	1.094(19)	1.094(19)	1.097(19)	1.095(19)	1.093(18)	1.095(19)
**C23**	**H23A**	1.104(13)	0.948(13)	1.099(17)	1.101(16)	1.098(16)	1.097(17)	1.100(16)	1.096(16)	1.08(2)	1.08(2)	1.08(2)	1.08(2)	1.08(2)	1.08(2)
**C23**	**H23B**	1.083(15)	0.948(13)	1.058(18)	1.058(17)	1.056(18)	1.056(18)	1.058(17)	1.056(17)	1.07(2)	1.06(2)	1.07(2)	1.06(2)	1.06(2)	1.06(2)
**C23**	**H23C**	1.073(12)	0.948(13)	1.085(17)	1.088(17)	1.085(17)	1.084(17)	1.090(17)	1.087(17)	1.09(2)	1.09(2)	1.09(2)	1.09(2)	1.09(2)	1.09(2)
**C24**	**H24A**	1.093(13)	0.959(12)	1.122(17)	1.125(17)	1.125(17)	1.124(17)	1.126(17)	1.125(17)	1.11(2)	1.11(2)	1.11(2)	1.11(2)	1.11(2)	1.11(2)
**C24**	**H24B**	1.081(13)	0.959(12)	1.088(17)	1.093(17)	1.091(17)	1.080(17)	1.087(17)	1.085(17)	1.08(3)	1.08(3)	1.08(3)	1.07(3)	1.07(2)	1.07(3)
**C24**	**H24C**	1.076(14)	0.959(12)	1.112(18)	1.111(18)	1.108(18)	1.108(18)	1.107(18)	1.105(18)	1.11(2)	1.11(2)	1.10(2)	1.10(2)	1.10(2)	1.10(2)

Table S18 X-H bond lengths (units: Å) obtained for SITKUB with various experimental methods (neutron and X-ray) and refinement methods (IAM and HAR). HAR was performed with a cluster of charges and dipoles modeling crystal environment (DiSCaMB) and without a cluster (NoSpherA2). DFT calculations for HAR were performed with various functionals in the non-relativistic version (B3LYP, PBE and M06-2X) and with relativistic correction (B3LYP-DKH2, PBE-DKH2 and M06-2X-DKH2). Basis sets used: (a) non-relativistic refinements: jorge-TZP, (b) relativistic refinements: jorge-TZP-DKH.

				DiSCaMB-HAR						NoSpherA2-HAR					
**bond**		**neutron**	**IAM**	**B3LYP**	**PBE**	**M06-2X**	**B3LYP-DKH2**	**PBE-DKH2**	**M06-2X-DKH2**	**B3LYP**	**PBE**	**M06-2X**	**B3LYP-DKH2**	**PBE-DKH2**	**M06-2X-DKH2**
**Rh**	**H**	1.531(11)	1.40(2)	1.429(14)	1.443(13)	1.456(13)	1.397(16)	1.418(15)	1.434(15)	1.474(17)	1.479(16)	1.491(16)	1.469(17)	1.472(16)	1.485(16)
**C3**	**H3**	1.080(16)	0.9488(NA)	1.097(14)	1.100(14)	1.097(14)	1.093(14)	1.096(14)	1.094(14)	1.111(19)	1.111(19)	1.111(19)	1.109(19)	1.108(19)	1.108(19)
**C4**	**H4**	1.081(12)	0.9485(NA)	1.066(13)	1.070(13)	1.067(14)	1.064(14)	1.067(14)	1.065(14)	1.071(19)	1.074(19)	1.073(19)	1.071(19)	1.073(19)	1.073(19)
**C5**	**H5**	1.098(11)	0.9482(NA)	1.069(13)	1.070(13)	1.068(13)	1.065(13)	1.066(13)	1.064(13)	1.072(18)	1.072(18)	1.072(18)	1.070(18)	1.070(18)	1.069(18)
**C6**	**H6**	1.071(14)	0.9474(NA)	1.093(13)	1.093(13)	1.090(13)	1.087(13)	1.088(13)	1.084(13)	1.092(17)	1.091(17)	1.090(17)	1.089(17)	1.087(17)	1.087(17)
**C7**	**H7**	1.100(9)	0.9988(NA)	1.107(13)	1.109(13)	1.108(13)	1.106(13)	1.108(13)	1.107(13)	1.112(18)	1.112(17)	1.113(17)	1.111(17)	1.111(17)	1.112(17)
**C8**	**H8A**	1.079(10)	0.964(12)	1.056(15)	1.059(15)	1.056(15)	1.054(15)	1.057(15)	1.054(15)	1.06(2)	1.06(2)	1.06(2)	1.06(2)	1.06(2)	1.06(2)
**C8**	**H8B**	1.054(16)	0.963(12)	1.131(14)	1.133(13)	1.131(13)	1.128(14)	1.131(14)	1.129(14)	1.134(19)	1.134(19)	1.132(19)	1.132(19)	1.132(19)	1.130(19)
**C8**	**H8C**	1.072(16)	0.965(12)	1.088(13)	1.092(13)	1.090(13)	1.091(14)	1.095(14)	1.093(14)	1.082(18)	1.083(18)	1.082(18)	1.081(18)	1.082(18)	1.081(18)
**C9**	**H9A**	1.059(18)	0.955(12)	1.098(13)	1.101(13)	1.099(13)	1.098(13)	1.101(13)	1.099(13)	1.106(18)	1.106(18)	1.107(18)	1.104(18)	1.103(18)	1.104(18)
**C9**	**H9B**	1.087(11)	0.955(12)	1.068(15)	1.071(15)	1.067(15)	1.066(15)	1.068(15)	1.065(15)	1.07(2)	1.07(2)	1.06(2)	1.06(2)	1.07(2)	1.06(2)
**C9**	**H9C**	1.085(10)	0.955(12)	1.064(15)	1.066(15)	1.062(15)	1.063(15)	1.064(16)	1.060(16)	1.06(2)	1.06(2)	1.06(2)	1.05(2)	1.05(2)	1.05(2)
**C10**	**H10**	1.085(13)	0.9986(NA)	1.108(12)	1.110(12)	1.109(12)	1.109(12)	1.111(12)	1.110(12)	1.109(17)	1.108(17)	1.110(17)	1.107(17)	1.105(17)	1.108(17)
**C11**	**H11A**	1.083(13)	0.965(13)	1.089(14)	1.093(14)	1.092(14)	1.085(14)	1.089(14)	1.088(15)	1.08(2)	1.08(2)	1.08(2)	1.08(2)	1.08(2)	1.08(2)
**C11**	**H11B**	1.083(11)	0.965(13)	1.083(16)	1.085(16)	1.081(16)	1.080(16)	1.082(16)	1.078(16)	1.09(2)	1.09(2)	1.08(2)	1.08(2)	1.08(2)	1.08(2)
**C11**	**H11C**	1.088(15)	0.966(13)	1.096(14)	1.098(14)	1.095(14)	1.099(14)	1.102(14)	1.099(15)	1.11(2)	1.11(2)	1.10(2)	1.10(2)	1.10(2)	1.10(2)
**C12**	**H12A**	1.071(13)	0.943(11)	1.066(15)	1.070(15)	1.066(15)	1.066(15)	1.070(15)	1.065(15)	1.08(2)	1.08(2)	1.07(2)	1.07(2)	1.07(2)	1.07(2)
**C12**	**H12B**	1.072(14)	0.943(11)	1.073(12)	1.074(12)	1.070(12)	1.074(13)	1.075(13)	1.071(13)	1.084(16)	1.084(16)	1.082(16)	1.082(16)	1.082(16)	1.081(16)
**C12**	**H12C**	1.092(13)	0.943(11)	1.084(14)	1.088(13)	1.084(14)	1.080(14)	1.084(14)	1.081(14)	1.084(18)	1.085(18)	1.085(18)	1.082(18)	1.083(18)	1.082(18)
**C13**	**H13**	1.089(14)	0.9989(NA)	1.094(13)	1.096(13)	1.094(13)	1.093(13)	1.096(13)	1.094(13)	1.098(17)	1.098(17)	1.099(17)	1.097(17)	1.097(17)	1.098(17)
**C14**	**H14A**	1.095(11)	0.974(13)	1.066(16)	1.069(16)	1.067(16)	1.064(16)	1.067(16)	1.065(16)	1.07(2)	1.07(2)	1.07(2)	1.07(2)	1.07(2)	1.07(2)
**C14**	**H14B**	1.073(14)	0.975(13)	1.082(15)	1.085(15)	1.081(15)	1.078(16)	1.081(16)	1.078(16)	1.10(2)	1.10(2)	1.09(2)	1.09(2)	1.10(2)	1.09(2)
**C14**	**H14C**	1.082(16)	0.976(13)	1.094(16)	1.098(16)	1.096(16)	1.095(16)	1.099(16)	1.098(16)	1.09(2)	1.09(2)	1.09(2)	1.09(2)	1.09(2)	1.09(2)
**C15**	**H15A**	1.045(19)	0.975(14)	1.086(16)	1.088(16)	1.087(16)	1.086(16)	1.088(16)	1.087(16)	1.10(2)	1.11(2)	1.10(2)	1.10(2)	1.10(2)	1.10(2)
**C15**	**H15B**	1.095(13)	0.975(14)	1.087(16)	1.090(16)	1.085(16)	1.086(16)	1.089(16)	1.083(16)	1.08(2)	1.08(2)	1.08(2)	1.08(2)	1.08(2)	1.08(2)
**C15**	**H15C**	1.090(11)	0.974(14)	1.086(15)	1.090(15)	1.088(15)	1.084(15)	1.088(15)	1.086(15)	1.10(2)	1.10(2)	1.10(2)	1.10(2)	1.10(2)	1.10(2)

**C16**	**H16**	1.094(11)	0.9983(NA)	1.091(13)	1.090(13)	1.089(13)	1.081(13)	1.080(13)	1.079(13)	1.088(17)	1.085(17)	1.086(17)	1.085(17)	1.082(17)	1.083(17)
**C17**	**H17A**	1.096(12)	0.968(13)	1.084(15)	1.086(15)	1.082(15)	1.086(16)	1.088(15)	1.083(15)	1.09(2)	1.094(19)	1.091(19)	1.091(19)	1.091(19)	1.089(19)
**C17**	**H17B**	1.096(14)	0.969(13)	1.057(15)	1.062(15)	1.058(15)	1.055(16)	1.060(15)	1.056(15)	1.10(2)	1.10(2)	1.10(2)	1.09(2)	1.10(2)	1.09(2)
**C17**	**H17C**	1.088(14)	0.968(13)	1.112(13)	1.116(13)	1.113(13)	1.114(13)	1.119(13)	1.115(14)	1.09(2)	1.09(2)	1.09(2)	1.09(2)	1.09(2)	1.09(2)
**C18**	**H18A**	1.077(13)	0.976(13)	1.081(14)	1.085(14)	1.083(14)	1.080(14)	1.084(14)	1.082(14)	1.084(18)	1.084(18)	1.086(18)	1.083(18)	1.085(18)	1.086(18)
**C18**	**H18B**	1.081(13)	0.976(13)	1.073(17)	1.076(17)	1.075(17)	1.071(17)	1.074(17)	1.073(17)	1.09(2)	1.09(2)	1.09(2)	1.09(2)	1.09(2)	1.09(2)
**C18**	**H18C**	1.085(13)	0.975(13)	1.074(15)	1.076(15)	1.073(15)	1.078(16)	1.080(16)	1.076(16)	1.08(2)	1.08(2)	1.08(2)	1.08(2)	1.08(2)	1.08(2)
**C19**	**H19**	1.103(13)	0.9982(NA)	1.054(14)	1.054(14)	1.051(14)	1.053(15)	1.054(15)	1.051(15)	1.061(18)	1.060(18)	1.059(18)	1.057(18)	1.056(18)	1.055(18)
**C20**	**H20A**	1.087(12)	0.957(14)	1.083(14)	1.088(14)	1.085(14)	1.077(15)	1.083(15)	1.079(15)	1.08(2)	1.08(2)	1.08(2)	1.08(2)	1.08(2)	1.08(2)
**C20**	**H20B**	1.078(14)	0.957(14)	1.114(16)	1.117(16)	1.112(16)	1.118(16)	1.121(16)	1.117(16)	1.11(2)	1.11(2)	1.11(2)	1.11(2)	1.11(2)	1.11(2)
**C20**	**H20C**	1.091(15)	0.956(14)	1.08(2)	1.08(2)	1.07(2)	1.08(2)	1.08(2)	1.08(2)	1.08(3)	1.08(3)	1.08(3)	1.08(3)	1.08(3)	1.08(3)
**C21**	**H21A**	1.049(17)	0.974(14)	1.072(18)	1.074(18)	1.070(19)	1.069(19)	1.071(19)	1.067(19)	1.07(3)	1.07(3)	1.07(3)	1.07(3)	1.07(3)	1.07(3)
**C21**	**H21B**	1.071(16)	0.973(14)	1.053(19)	1.058(19)	1.054(19)	1.050(19)	1.056(19)	1.052(19)	1.05(3)	1.05(3)	1.05(3)	1.05(3)	1.05(3)	1.05(3)
**C21**	**H21C**	1.077(15)	0.975(14)	1.092(14)	1.097(14)	1.095(14)	1.089(15)	1.096(15)	1.094(15)	1.101(19)	1.103(19)	1.103(19)	1.101(19)	1.103(19)	1.104(19)
**C22**	**H22**	1.077(12)	0.9984(NA)	1.102(12)	1.105(12)	1.103(12)	1.103(12)	1.106(12)	1.104(12)	1.091(17)	1.090(17)	1.092(17)	1.091(17)	1.091(16)	1.092(16)
**C23**	**H23A**	1.104(13)	0.948(13)	1.076(15)	1.080(15)	1.076(15)	1.074(15)	1.078(15)	1.074(15)	1.07(2)	1.07(2)	1.07(2)	1.07(2)	1.07(2)	1.07(2)
**C23**	**H23B**	1.083(15)	0.948(13)	1.049(15)	1.052(15)	1.048(16)	1.049(16)	1.051(16)	1.048(16)	1.06(2)	1.06(2)	1.06(2)	1.06(2)	1.06(2)	1.06(2)
**C23**	**H23C**	1.073(12)	0.948(13)	1.073(15)	1.076(15)	1.072(15)	1.076(15)	1.080(15)	1.075(15)	1.08(2)	1.084(19)	1.082(19)	1.081(19)	1.084(19)	1.081(19)
**C24**	**H24A**	1.093(13)	0.959(12)	1.116(14)	1.118(14)	1.117(14)	1.120(14)	1.122(14)	1.121(14)	1.110(19)	1.109(19)	1.111(19)	1.110(19)	1.109(19)	1.110(19)
**C24**	**H24B**	1.081(13)	0.959(12)	1.063(15)	1.068(15)	1.065(15)	1.055(16)	1.059(16)	1.055(16)	1.06(2)	1.06(2)	1.06(2)	1.06(2)	1.06(2)	1.06(2)
**C24**	**H24C**	1.076(14)	0.959(12)	1.076(16)	1.078(16)	1.074(16)	1.074(16)	1.075(16)	1.072(17)	1.08(2)	1.08(2)	1.08(2)	1.08(2)	1.08(2)	1.07(2)

Table S19 X-H bond lengths (units: Å) obtained for UJABOX with various experimental methods (neutron and X-ray) and refinement methods (IAM and HAR). HAR was performed with a cluster of charges and dipoles modeling crystal environment (DiSCaMB) and without a cluster (NoSpherA2). DFT calculations for HAR were performed with various functionals in the non-relativistic version (B3LYP, PBE and M06-2X) and with relativistic correction (B3LYP-DKH2, PBE-DKH2 and M06-2X-DKH2). Basis sets used: (a) non-relativistic refinements: cc-pVTZ-DK, (b) relativistic refinements: cc-pVTZ-DK.

				DiSCaMB-HAR						NoSpherA2-HAR					
**bond**		**neutron**	**IAM**	**B3LYP**	**PBE**	**M06-2X**	**B3LYP-DKH2**	**PBE-DKH2**	**M06-2X-DKH2**	**B3LYP**	**PBE**	**M06-2X**	**B3LYP-DKH2**	**PBE-DKH2**	**M06-2X-DKH2**
**Ru1**	**H10**	1.559(7)	1.35(11)	1.31(10)	1.34(10)	1.38(9)	1.28(10)	1.32(10)	1.37(9)	1.27(10)	1.26(10)	1.35(9)	1.31(10)	1.30(10)	1.38(9)
**Si2**	**H11**	1.481(5)	1.41(3)	1.48(3)	1.49(3)	1.49(3)	1.48(3)	1.49(3)	1.49(3)	1.48(3)	1.49(3)	1.49(3)	1.48(3)	1.49(3)	1.49(3)
**C3**	**H3**	1.092(4)	0.88(3)	1.04(3)	1.04(3)	1.04(3)	1.03(3)	1.04(3)	1.03(3)	1.03(3)	1.04(3)	1.04(3)	1.03(3)	1.04(3)	1.03(3)
**C2**	**H2**	1.085(4)	0.87(3)	1.05(3)	1.05(3)	1.05(3)	1.05(4)	1.05(3)	1.05(3)	1.05(3)	1.05(3)	1.05(3)	1.05(3)	1.05(3)	1.05(3)
**C1**	**H1**	1.087(4)	0.91(3)	1.08(4)	1.08(4)	1.08(4)	1.08(4)	1.08(4)	1.08(4)	1.08(4)	1.08(4)	1.08(4)	1.08(4)	1.08(4)	1.07(3)
**C4**	**H4**	1.083(4)	0.90(3)	1.04(3)	1.05(3)	1.04(3)	1.04(3)	1.04(3)	1.04(3)	1.04(3)	1.04(3)	1.04(3)	1.04(3)	1.04(3)	1.04(3)
**C6**	**H6A**	1.094(5)	0.93(4)	1.11(4)	1.11(4)	1.10(4)	1.11(4)	1.11(4)	1.10(4)	1.11(4)	1.11(4)	1.10(4)	1.10(4)	1.10(4)	1.10(4)
**C6**	**H6B**	1.088(5)	0.94(5)	1.10(5)	1.11(5)	1.11(5)	1.10(5)	1.11(5)	1.11(5)	1.10(5)	1.10(5)	1.11(5)	1.10(5)	1.10(5)	1.10(5)
**C6**	**H6C**	1.099(5)	0.93(5)	1.08(4)	1.08(4)	1.08(4)	1.08(4)	1.09(4)	1.08(4)	1.08(4)	1.08(4)	1.08(4)	1.08(4)	1.08(4)	1.08(4)
**C7**	**H7A**	1.091(5)	1.00(4)	1.14(4)	1.15(4)	1.14(4)	1.15(4)	1.15(4)	1.14(4)	1.14(4)	1.15(4)	1.15(4)	1.14(4)	1.14(4)	1.14(4)
**C7**	**H7B**	1.099(5)	1.01(4)	1.08(4)	1.08(4)	1.08(4)	1.08(4)	1.09(4)	1.08(4)	1.08(4)	1.09(4)	1.08(4)	1.08(4)	1.09(4)	1.08(4)
**C7**	**H7C**	1.084(5)	0.87(4)	1.05(4)	1.05(4)	1.05(4)	1.05(4)	1.05(4)	1.05(4)	1.05(4)	1.05(4)	1.05(4)	1.05(4)	1.05(4)	1.05(4)
**C8**	**H8A**	1.081(5)	0.92(5)	1.07(5)	1.08(5)	1.07(5)	1.07(5)	1.07(5)	1.07(5)	1.07(5)	1.07(5)	1.07(5)	1.07(5)	1.07(5)	1.07(5)
**C8**	**H8B**	1.092(5)	0.87(4)	1.01(5)	1.01(5)	1.01(5)	1.01(5)	1.01(5)	1.01(5)	1.01(5)	1.01(5)	1.01(5)	1.01(5)	1.01(5)	1.01(5)
**C8**	**H8C**	1.090(5)	0.98(6)	1.12(6)	1.12(6)	1.12(6)	1.12(6)	1.12(6)	1.12(6)	1.12(6)	1.12(6)	1.12(6)	1.12(6)	1.12(6)	1.12(6)
**C9**	**H9A**	1.086(6)	0.92(6)	1.09(6)	1.10(6)	1.10(6)	1.09(6)	1.10(6)	1.10(6)	1.10(6)	1.10(6)	1.10(6)	1.09(6)	1.09(6)	1.10(6)
**C9**	**H9B**	1.088(5)	0.87(5)	0.97(5)	0.98(5)	0.97(5)	0.97(5)	0.97(5)	0.97(5)	0.97(5)	0.98(5)	0.97(5)	0.97(5)	0.97(5)	0.97(5)
**C9**	**H9C**	1.084(6)	0.96(6)	1.12(6)	1.13(6)	1.13(6)	1.13(6)	1.13(6)	1.13(6)	1.13(6)	1.13(6)	1.13(6)	1.12(6)	1.13(6)	1.13(6)

Table S20 X-H bond lengths (units: Å) obtained for ZEYVAA with various experimental methods (neutron and X-ray) and refinement methods (IAM and HAR). HAR was performed with a cluster of charges and dipoles modeling crystal environment (DiSCaMB) and without a cluster (NoSpherA2). DFT calculations for HAR were performed with various functionals in the non-relativistic version (B3LYP, PBE and M06-2X) and with relativistic correction (B3LYP-DKH2, PBE-DKH2 and M06-2X-DKH2). Basis sets used: (a) non-relativistic refinements: cc-pVTZ-DK, (b) relativistic refinements: cc-pVTZ-DK.

				DiSCaMB-HAR						NoSpherA2-HAR					
**bond**		**neutron**	**IAM**	**B3LYP**	**PBE**	**M06-2X**	**B3LYP-DKH2**	**PBE-DKH2**	**M06-2X-DKH2**	**B3LYP**	**PBE**	**M06-2X**	**B3LYP-DKH2**	**PBE-DKH2**	**M06-2X-DKH2**
**Nb**	**H**	1.816(8)	1.80(8)	1.88(7)	1.87(7)	1.89(7)	1.89(7)	1.88(7)	1.87(7)	1.89(9)	1.89(9)	1.90(9)	1.90(8)	1.89(9)	1.89(9)
**C3**	**H3A**	1.081(6)	0.86(6)	0.99(6)	0.99(6)	0.99(6)	0.99(6)	0.99(6)	0.99(6)	0.99(6)	0.99(6)	0.99(6)	0.99(6)	0.99(6)	0.99(6)
**C2**	**H2A**	1.094(6)	1.00(6)	1.12(6)	1.13(6)	1.12(6)	1.13(6)	1.13(6)	1.12(6)	1.14(6)	1.13(6)	1.14(6)	1.13(6)	1.13(6)	1.13(6)
**C5**	**H5A**	1.095(9)	0.91(6)	1.05(7)	1.05(7)	1.04(7)	1.05(7)	1.04(7)	1.04(7)	1.05(7)	1.05(7)	1.06(7)	1.05(7)	1.05(7)	1.05(7)
**C6**	**H6A**	1.069(7)	0.85(7)	1.03(9)	1.03(9)	1.03(9)	1.03(9)	1.03(9)	1.02(9)	1.04(9)	1.03(9)	1.04(9)	1.03(9)	1.03(9)	1.03(9)
**C4**	**H4A**	1.090(6)	0.96(6)	1.10(7)	1.11(7)	1.10(7)	1.10(7)	1.11(7)	1.09(7)	1.11(7)	1.10(7)	1.11(7)	1.10(7)	1.10(7)	1.10(7)
**C8**	**H8A**	1.087(9)	0.96(8)	1.14(8)	1.13(8)	1.13(8)	1.13(8)	1.12(8)	1.12(8)	1.14(9)	1.13(8)	1.13(8)	1.13(8)	1.13(8)	1.13(8)
**C8**	**H8B**	1.095(7)	1.02(7)	1.13(7)	1.13(7)	1.13(7)	1.14(7)	1.14(7)	1.14(7)	1.16(8)	1.14(7)	1.16(8)	1.14(7)	1.14(7)	1.14(7)
**C8**	**H8C**	1.094(8)	0.95(6)	1.06(8)	1.06(8)	1.06(8)	1.07(8)	1.06(8)	1.06(7)	1.08(8)	1.06(8)	1.07(8)	1.07(8)	1.07(8)	1.07(8)
**C1**	**H1A**	1.083(8)	0.97(6)	1.13(7)	1.13(7)	1.13(7)	1.13(7)	1.13(7)	1.13(7)	1.13(7)	1.13(7)	1.13(7)	1.13(7)	1.13(7)	1.13(7)
**C7**	**H7A**	1.083(7)	0.96(7)	1.09(7)	1.10(7)	1.09(7)	1.09(7)	1.09(7)	1.09(7)	1.10(8)	1.09(7)	1.10(7)	1.09(7)	1.09(7)	1.10(8)
**C7**	**H7B**	1.091(8)	0.92(7)	1.10(8)	1.10(8)	1.10(8)	1.11(8)	1.11(7)	1.09(7)	1.13(8)	1.11(8)	1.12(8)	1.11(8)	1.11(8)	1.11(8)
**C7**	**H7C**	1.081(7)	0.97(7)	1.11(8)	1.11(8)	1.11(8)	1.11(8)	1.11(8)	1.11(8)	1.12(8)	1.12(8)	1.12(8)	1.12(8)	1.12(8)	1.12(8)

Table S21 X-H bond lengths (units: Å) obtained for GOJNIF with various experimental methods (neutron and X-ray) and refinement methods (IAM and HAR). HAR was performed with a cluster of charges and dipoles modeling crystal environment (DiSCaMB) and without a cluster (NoSpherA2). DFT calculations for HAR were performed with various functionals in the non-relativistic version (B3LYP, PBE and M06-2X) and with relativistic correction (B3LYP-DKH2, PBE-DKH2 and M06-2X-DKH2). Basis sets used: (a) non-relativistic refinements: cc-pVTZ-DK, (b) relativistic refinements: cc-pVTZ-DK.

				DiSCaMB-HAR						NoSpherA2-HAR					
**bond**		**neutron**	**IAM**	**B3LYP**	**PBE**	**M06-2X**	**B3LYP-DKH2**	**PBE-DKH2**	**M06-2X-DKH2**	**B3LYP**	**PBE**	**M06-2X**	**B3LYP-DKH2**	**PBE-DKH2**	**M06-2X-DKH2**
**Ni1**	**H1A**	1.61(2)	1.64(2)	1.66(2)	NA	NA	1.52(15)	NA	1.616(14)	1.65(2)	1.66(2)	1.65(2)	1.65(2)	1.66(2)	1.65(2)
**Ni1**	**H1B**	1.61(2)	1.58(2)	1.68(3)	NA	NA	1.626(14)	NA	1.54(13)	1.67(3)	1.67(3)	1.66(2)	1.68(3)	1.68(3)	1.67(2)
**C2**	**H2A**	1.10(3)	0.95	1.069(13)	NA	NA	1.070(13)	NA	1.071(13)	1.071(13)	1.070(13)	1.073(13)	1.068(13)	1.067(13)	1.069(13)
**C3**	**H3A**	1.15(2)	0.95	1.077(13)	NA	NA	1.073(13)	NA	1.067(13)	1.076(14)	1.078(14)	1.070(14)	1.075(13)	1.076(13)	1.069(13)
**C4**	**H4A**	1.13(2)	0.95(NA)	1.098(13)	NA	NA	1.094(13)	NA	1.094(12)	1.097(13)	1.096(13)	1.097(13)	1.096(13)	1.096(13)	1.096(12)
**C5**	**H5A**	1.12(3)	0.95(NA)	1.091(12)	NA	NA	1.081(11)	NA	1.076(11)	1.091(12)	1.090(12)	1.086(12)	1.090(11)	1.090(11)	1.086(11)
**C7**	**H7A**	1.15(2)	0.99(NA)	1.114(13)	NA	NA	1.110(12)	NA	1.106(12)	1.111(13)	1.108(13)	1.106(13)	1.113(13)	1.110(12)	1.109(13)
**C7**	**H7B**	1.14(2)	0.99(NA)	1.110(14)	NA	NA	1.107(14)	NA	1.105(14)	1.113(14)	1.112(14)	1.112(14)	1.111(14)	1.110(14)	1.109(14)
**C8**	**H8A**	1.13(3)	1(NA)	1.102(14)	NA	NA	1.102(14)	NA	1.100(13)	1.102(14)	1.105(14)	1.101(14)	1.101(14)	1.103(14)	1.099(14)
**C9**	**H9A**	1.13(2)	1(NA)	1.120(14)	NA	NA	1.120(13)	NA	1.117(13)	1.122(14)	1.122(14)	1.121(13)	1.122(13)	1.120(13)	1.119(13)
**C10**	**H10A**	1.08(3)	0.98(NA)	1.074(18)	NA	NA	1.068(17)	NA	1.071(17)	1.073(18)	1.079(18)	1.076(18)	1.072(18)	1.077(17)	1.074(18)
**C10**	**H10B**	1.07(4)	0.98(NA)	1.109(16)	NA	NA	1.102(15)	NA	1.105(15)	1.107(17)	1.109(17)	1.099(17)	1.108(17)	1.110(17)	1.100(16)
**C10**	**H10C**	1.11(3)	0.98(NA)	1.105(17)	NA	NA	1.100(17)	NA	1.091(16)	1.112(16)	1.113(16)	1.116(16)	1.111(15)	1.113(15)	1.115(15)
**C11**	**H11A**	1.11(3)	0.98(NA)	1.062(19)	NA	NA	1.073(18)	NA	1.075(18)	1.063(19)	1.065(19)	1.066(19)	1.063(19)	1.064(18)	1.065(18)
**C11**	**H11B**	1.11(3)	0.98(NA)	1.101(18)	NA	NA	1.104(18)	NA	1.104(18)	1.044(17)	1.043(16)	1.044(16)	1.043(16)	1.043(16)	1.044(16)
**C11**	**H11C**	1.10(3)	0.98(NA)	1.047(16)	NA	NA	1.036(16)	NA	1.036(16)	1.099(18)	1.097(18)	1.100(18)	1.098(18)	1.098(18)	1.099(18)
**C12**	**H12A**	1.10(3)	0.98(NA)	1.082(15)	NA	NA	1.068(15)	NA	1.066(15)	1.079(15)	1.081(15)	1.078(15)	1.079(15)	1.080(15)	1.077(15)
**C12**	**H12B**	1.11(3)	0.98(NA)	1.099(16)	NA	NA	1.091(16)	NA	1.087(16)	1.094(16)	1.100(15)	1.098(15)	1.093(15)	1.097(15)	1.096(15)
**C12**	**H12C**	1.09(4)	0.98(NA)	1.092(15)	NA	NA	1.095(15)	NA	1.098(15)	1.100(17)	1.101(16)	1.096(16)	1.101(16)	1.102(16)	1.097(16)
**C13**	**H13A**	1.11(3)	0.98(NA)	1.080(16)	NA	NA	1.068(16)	NA	1.068(16)	1.075(16)	1.075(16)	1.075(16)	1.074(16)	1.075(16)	1.074(16)
**C13**	**H13B**	1.05(4)	0.98(NA)	1.100(17)	NA	NA	1.096(17)	NA	1.091(16)	1.118(16)	1.121(16)	1.119(16)	1.117(16)	1.120(16)	1.117(16)
**C13**	**H13C**	1.12(4)	0.98(NA)	1.117(16)	NA	NA	1.115(16)	NA	1.114(15)	1.108(17)	1.112(17)	1.105(17)	1.105(17)	1.107(17)	1.100(17)
**C15**	**H15A**	1.10(3)	0.95(NA)	1.096(12)	NA	NA	1.095(12)	NA	1.088(12)	1.098(12)	1.097(12)	1.091(12)	1.097(12)	1.096(12)	1.090(12)
**C16**	**H16A**	1.15(2)	0.95(NA)	1.092(13)	NA	NA	1.085(13)	NA	1.084(13)	1.092(13)	1.094(14)	1.091(14)	1.091(13)	1.092(13)	1.090(13)
**C17**	**H17A**	1.13(2)	0.95(NA)	1.091(15)	NA	NA	1.097(14)	NA	1.097(14)	1.089(15)	1.087(15)	1.088(15)	1.089(14)	1.088(14)	1.089(14)
**C18**	**H18A**	1.13(3)	0.95(NA)	1.104(14)	NA	NA	1.104(14)	NA	1.104(14)	1.103(14)	1.104(14)	1.102(14)	1.104(14)	1.103(14)	1.104(14)

**C20**	**H20A**	1.12(2)	0.99(NA)	1.108(14)	NA	NA	1.102(13)	NA	1.100(13)	1.106(14)	1.107(14)	1.104(14)	1.105(14)	1.105(13)	1.104(13)
**C20**	**H20B**	1.13(2)	0.99(NA)	1.111(12)	NA	NA	1.110(12)	NA	1.108(12)	1.113(12)	1.112(12)	1.112(12)	1.112(12)	1.111(12)	1.111(12)
**C21**	**H21A**	1.16(2)	1(NA)	1.116(13)	NA	NA	1.105(13)	NA	1.102(13)	1.116(13)	1.116(13)	1.113(13)	1.115(13)	1.115(13)	1.112(13)
**C22**	**H22A**	1.13(2)	1(NA)	1.101(13)	NA	NA	1.095(13)	NA	1.095(13)	1.100(13)	1.099(14)	1.100(13)	1.098(13)	1.097(13)	1.099(13)
**C23**	**H23A**	1.12(3)	0.98(NA)	1.108(15)	NA	NA	1.105(14)	NA	1.105(14)	1.108(15)	1.109(15)	1.109(15)	1.106(15)	1.106(14)	1.106(14)
**C23**	**H23B**	1.06(4)	0.98(NA)	1.091(15)	NA	NA	1.094(15)	NA	1.089(15)	1.078(16)	1.077(16)	1.080(16)	1.078(16)	1.076(15)	1.078(15)
**C23**	**H23C**	1.08(3)	0.98(NA)	1.079(16)	NA	NA	1.077(15)	NA	1.077(15)	1.095(15)	1.097(15)	1.090(15)	1.092(15)	1.092(15)	1.087(15)
**C24**	**H24A**	1.06(3)	0.98(NA)	1.159(16)	NA	NA	1.153(15)	NA	1.144(15)	1.159(16)	1.151(16)	1.150(16)	1.158(16)	1.153(15)	1.150(15)
**C24**	**H24B**	1.12(3)	0.98(NA)	1.099(15)	NA	NA	1.098(15)	NA	1.096(15)	1.074(14)	1.074(14)	1.072(14)	1.075(14)	1.075(14)	1.074(14)
**C24**	**H24C**	1.08(3)	0.98(NA)	1.073(14)	NA	NA	1.072(14)	NA	1.070(14)	1.099(15)	1.100(15)	1.096(15)	1.099(15)	1.100(15)	1.097(15)
**C25**	**H25A**	1.14(2)	0.98(NA)	1.106(13)	NA	NA	1.098(13)	NA	1.095(13)	1.106(13)	1.106(13)	1.103(13)	1.107(13)	1.107(13)	1.104(13)
**C25**	**H25B**	1.10(3)	0.98(NA)	1.062(15)	NA	NA	1.059(15)	NA	1.061(15)	1.089(13)	1.093(13)	1.087(13)	1.089(13)	1.091(12)	1.086(12)
**C25**	**H25C**	1.11(3)	0.98(NA)	1.089(13)	NA	NA	1.092(12)	NA	1.089(12)	1.063(15)	1.066(15)	1.067(15)	1.063(15)	1.064(15)	1.065(15)
**C26**	**H26A**	1.06(3)	0.98(NA)	1.095(15)	NA	NA	1.093(14)	NA	1.092(14)	1.091(15)	1.089(15)	1.088(15)	1.093(15)	1.093(14)	1.091(14)
**C26**	**H26B**	1.14(3)	0.98(NA)	1.095(15)	NA	NA	1.088(14)	NA	1.084(14)	1.077(15)	1.080(15)	1.076(15)	1.075(15)	1.077(15)	1.073(15)
**C26**	**H26C**	1.13(3)	0.98(NA)	1.073(15)	NA	NA	1.073(15)	NA	1.071(14)	1.093(15)	1.093(15)	1.089(15)	1.093(15)	1.094(15)	1.089(14)
**C28**	**H28A**	1.12(2)	0.95(NA)	1.084(13)	NA	NA	1.080(13)	NA	1.082(13)	1.087(13)	1.090(13)	1.091(13)	1.084(13)	1.085(13)	1.086(13)
**C29**	**H29A**	1.10(3)	0.95(NA)	1.065(15)	NA	NA	1.050(15)	NA	1.050(15)	1.064(15)	1.065(16)	1.065(15)	1.062(15)	1.062(15)	1.062(15)
**C30**	**H30A**	1.13(2)	0.95(NA)	1.091(15)	NA	NA	1.091(14)	NA	1.087(14)	1.093(15)	1.094(15)	1.088(15)	1.093(14)	1.093(14)	1.088(14)
**C31**	**H31A**	1.12(2)	0.95(NA)	1.065(13)	NA	NA	1.063(13)	NA	1.060(13)	1.066(13)	1.066(13)	1.063(13)	1.065(13)	1.064(13)	1.063(13)
**C33**	**H33A**	1.16(2)	0.99(NA)	1.107(12)	NA	NA	1.112(12)	NA	1.110(12)	1.108(12)	1.108(12)	1.106(12)	1.109(12)	1.108(12)	1.107(12)
**C33**	**H33B**	1.12(2)	0.99(NA)	1.104(11)	NA	NA	1.103(11)	NA	1.101(11)	1.105(11)	1.105(11)	1.104(11)	1.104(11)	1.104(11)	1.103(11)
**C34**	**H34A**	1.13(2)	1(NA)	1.105(13)	NA	NA	1.105(12)	NA	1.100(12)	1.108(13)	1.104(13)	1.104(13)	1.107(13)	1.103(12)	1.102(12)
**C35**	**H35A**	1.13(2)	1(NA)	1.109(13)	NA	NA	1.101(13)	NA	1.097(13)	1.106(14)	1.106(14)	1.103(14)	1.105(13)	1.104(13)	1.102(13)
**C36**	**H36A**	1.08(3)	0.98(NA)	1.086(14)	NA	NA	1.080(14)	NA	1.075(14)	1.090(15)	1.089(14)	1.084(14)	1.089(14)	1.089(14)	1.083(14)
**C36**	**H36B**	1.15(3)	0.98(NA)	1.118(15)	NA	NA	1.120(14)	NA	1.117(14)	1.098(14)	1.103(14)	1.097(14)	1.097(14)	1.101(14)	1.095(14)
**C36**	**H36C**	1.08(3)	0.98(NA)	1.100(14)	NA	NA	1.095(14)	NA	1.093(13)	1.117(15)	1.119(15)	1.115(15)	1.118(15)	1.120(15)	1.115(14)
**C37**	**H37A**	1.10(3)	0.98(NA)	1.080(17)	NA	NA	1.083(16)	NA	1.084(16)	1.076(17)	1.078(17)	1.078(17)	1.077(17)	1.078(16)	1.078(16)
**C37**	**H37B**	1.09(3)	0.98(NA)	1.067(14)	NA	NA	1.065(14)	NA	1.064(14)	1.080(15)	1.079(15)	1.079(15)	1.078(15)	1.078(15)	1.077(15)
**C37**	**H37C**	1.09(3)	0.98(NA)	1.078(15)	NA	NA	1.075(15)	NA	1.073(15)	1.067(14)	1.066(14)	1.065(14)	1.068(14)	1.068(14)	1.067(14)
**C38**	**H38A**	1.14(2)	0.98(NA)	1.081(15)	NA	NA	1.071(15)	NA	1.070(15)	1.075(16)	1.074(16)	1.074(16)	1.074(15)	1.074(15)	1.073(15)
**C38**	**H38B**	1.10(3)	0.98(NA)	1.102(14)	NA	NA	1.107(14)	NA	1.107(14)	1.103(15)	1.101(15)	1.095(15)	1.102(15)	1.101(15)	1.095(15)
**C38**	**H38C**	1.11(3)	0.98(NA)	1.099(15)	NA	NA	1.091(15)	NA	1.083(15)	1.107(14)	1.108(14)	1.107(14)	1.107(14)	1.108(14)	1.106(14)
**C39**	**H39A**	1.08(3)	0.98(NA)	1.09(2)	NA	NA	1.092(19)	NA	1.093(19)	1.09(2)	1.10(2)	1.10(2)	1.091(19)	1.093(19)	1.091(19)

**C39**	**H39B**	1.08(3)	0.98(NA)	1.108(16)	NA	NA	1.104(16)	NA	1.101(16)	1.085(16)	1.086(16)	1.082(15)	1.083(15)	1.084(15)	1.080(15)
**C39**	**H39C**	1.12(3)	0.98(NA)	1.081(15)	NA	NA	1.085(15)	NA	1.081(15)	1.108(16)	1.111(16)	1.107(16)	1.107(16)	1.109(15)	1.106(15)

Table S22 X-H bond lengths (units: Å) obtained for GOJNIF with various experimental methods (neutron and X-ray) and refinement methods (IAM and HAR). HAR was performed with a cluster of charges and dipoles modeling crystal environment (DiSCaMB) and without a cluster (NoSpherA2). DFT calculations for HAR were performed with various functionals in the non-relativistic version (B3LYP, PBE and M06-2X) and with relativistic correction (B3LYP-DKH2, PBE-DKH2 and M06-2X-DKH2). Basis sets used: (a) non-relativistic refinements: jorge-DZP, (b) relativistic refinements: jorge-DZP-DKH.

				DiSCaMB-HAR						NoSpherA2-HAR					
**bond**		**neutron**	**IAM**	**B3LYP**	**PBE**	**M06-2X**	**B3LYP-DKH2**	**PBE-DKH2**	**M06-2X-DKH2**	**B3LYP**	**PBE**	**M06-2X**	**B3LYP-DKH2**	**PBE-DKH2**	**M06-2X-DKH2**
**Ni1**	**H1A**	1.61(2)	1.64(2)	1.66(4)	1.66(4)	1.67(4)	1.68(4)	1.67(4)	1.67(4)	1.67(4)	1.67(4)	1.66(4)	1.65(4)	1.65(4)	1.66(4)
**Ni1**	**H1B**	1.61(2)	1.58(2)	1.74(4)	1.73(4)	1.73(4)	1.71(3)	1.72(3)	1.71(3)	1.72(4)	1.72(4)	1.74(4)	1.75(4)	1.74(4)	1.73(4)
**C2**	**H2A**	1.10(3)	0.95	1.057(19)	1.062(19)	1.064(19)	1.060(18)	1.064(18)	1.062(19)	1.060(18)	1.060(19)	1.064(19)	1.059(19)	1.058(18)	1.064(18)
**C3**	**H3A**	1.15(2)	0.95	1.10(2)	1.10(2)	1.10(2)	1.10(2)	1.10(2)	1.10(2)	1.10(2)	1.10(2)	1.10(2)	1.10(2)	1.10(2)	1.10(2)
**C4**	**H4A**	1.13(2)	0.95(NA)	1.09(2)	1.091(19)	1.09(2)	1.088(18)	1.091(18)	1.091(18)	1.087(19)	1.087(19)	1.09(2)	1.090(19)	1.088(19)	1.092(19)
**C5**	**H5A**	1.12(3)	0.95(NA)	1.13(2)	1.126(19)	1.12(2)	1.123(18)	1.121(18)	1.118(18)	1.124(19)	1.124(19)	1.13(2)	1.130(19)	1.130(19)	1.125(19)
**C7**	**H7A**	1.15(2)	0.99(NA)	1.116(19)	1.117(19)	1.116(19)	1.114(18)	1.115(18)	1.115(18)	1.116(18)	1.115(18)	1.117(19)	1.116(18)	1.113(18)	1.116(18)
**C7**	**H7B**	1.14(2)	0.99(NA)	1.09(2)	1.09(2)	1.09(2)	1.10(2)	1.10(2)	1.10(2)	1.09(2)	1.09(2)	1.09(2)	1.10(2)	1.10(2)	1.10(2)
**C8**	**H8A**	1.13(3)	1(NA)	1.13(2)	1.13(2)	1.13(2)	1.13(2)	1.13(2)	1.13(2)	1.13(2)	1.13(2)	1.13(2)	1.13(2)	1.13(2)	1.13(2)
**C9**	**H9A**	1.13(2)	1(NA)	1.14(2)	1.14(2)	1.14(2)	1.14(2)	1.14(2)	1.14(2)	1.14(2)	1.14(2)	1.14(2)	1.14(2)	1.14(2)	1.14(2)
**C10**	**H10A**	1.08(3)	0.98(NA)	1.12(3)	1.12(3)	1.12(3)	1.11(3)	1.12(3)	1.11(3)	1.11(3)	1.11(3)	1.12(3)	1.11(3)	1.11(3)	1.12(3)
**C10**	**H10B**	1.07(4)	0.98(NA)	1.12(2)	1.12(2)	1.12(2)	1.12(2)	1.13(2)	1.13(2)	1.12(2)	1.12(2)	1.12(3)	1.12(3)	1.12(2)	1.12(2)
**C10**	**H10C**	1.11(3)	0.98(NA)	1.12(3)	1.12(3)	1.12(3)	1.12(2)	1.12(2)	1.12(2)	1.12(2)	1.12(2)	1.12(2)	1.12(2)	1.12(2)	1.13(2)
**C11**	**H11A**	1.11(3)	0.98(NA)	1.12(3)	1.12(3)	1.12(3)	1.11(3)	1.11(3)	1.11(3)	1.11(3)	1.11(3)	1.12(3)	1.11(3)	1.11(3)	1.12(3)
**C11**	**H11B**	1.11(3)	0.98(NA)	1.12(3)	1.12(3)	1.12(3)	1.11(3)	1.11(3)	1.11(3)	1.04(3)	1.04(3)	1.04(3)	1.04(3)	1.04(3)	1.04(3)
**C11**	**H11C**	1.10(3)	0.98(NA)	1.04(3)	1.05(3)	1.05(3)	1.04(3)	1.04(3)	1.04(3)	1.11(3)	1.11(3)	1.12(3)	1.11(3)	1.11(3)	1.12(3)
**C12**	**H12A**	1.10(3)	0.98(NA)	1.10(2)	1.10(2)	1.10(2)	1.09(2)	1.09(2)	1.09(2)	1.09(2)	1.09(2)	1.10(2)	1.10(2)	1.10(2)	1.10(2)
**C12**	**H12B**	1.11(3)	0.98(NA)	1.11(3)	1.11(3)	1.11(3)	1.11(2)	1.11(2)	1.11(2)	1.13(2)	1.13(2)	1.13(2)	1.13(2)	1.13(2)	1.13(2)
**C12**	**H12C**	1.09(4)	0.98(NA)	1.13(2)	1.14(2)	1.13(2)	1.13(2)	1.14(2)	1.13(2)	1.11(2)	1.11(2)	1.11(3)	1.11(2)	1.11(2)	1.11(2)
**C13**	**H13A**	1.11(3)	0.98(NA)	1.11(3)	1.11(3)	1.11(3)	1.10(2)	1.10(2)	1.10(2)	1.10(3)	1.10(3)	1.11(3)	1.11(3)	1.11(3)	1.11(3)
**C13**	**H13B**	1.05(4)	0.98(NA)	1.12(3)	1.12(2)	1.12(2)	1.12(2)	1.12(2)	1.12(2)	1.11(2)	1.11(2)	1.12(2)	1.12(2)	1.12(2)	1.12(2)
**C13**	**H13C**	1.12(4)	0.98(NA)	1.11(2)	1.12(2)	1.12(2)	1.12(2)	1.12(2)	1.12(2)	1.12(2)	1.12(2)	1.12(2)	1.12(2)	1.12(2)	1.12(2)
**C15**	**H15A**	1.10(3)	0.95(NA)	1.13(2)	1.13(2)	1.13(2)	1.130(18)	1.129(18)	1.126(18)	1.130(19)	1.130(19)	1.13(2)	1.135(19)	1.132(19)	1.131(19)
**C16**	**H16A**	1.15(2)	0.95(NA)	1.11(2)	1.12(2)	1.11(2)	1.11(2)	1.113(19)	1.11(2)	1.11(2)	1.11(2)	1.11(2)	1.11(2)	1.11(2)	1.11(2)
**C17**	**H17A**	1.13(2)	0.95(NA)	1.11(2)	1.11(2)	1.11(2)	1.11(2)	1.11(2)	1.11(2)	1.11(2)	1.11(2)	1.11(2)	1.11(2)	1.11(2)	1.11(2)
**C18**	**H18A**	1.13(3)	0.95(NA)	1.10(2)	1.10(2)	1.10(2)	1.101(19)	1.102(19)	1.101(19)	1.10(2)	1.10(2)	1.10(2)	1.10(2)	1.099(19)	1.100(19)

**C20**	**H20A**	1.12(2)	0.99(NA)	1.15(2)	1.15(2)	1.15(2)	1.15(2)	1.147(19)	1.15(2)	1.15(2)	1.15(2)	1.15(2)	1.15(2)	1.15(2)	1.15(2)
**C20**	**H20B**	1.13(2)	0.99(NA)	1.102(19)	1.103(19)	1.100(19)	1.105(18)	1.106(18)	1.104(18)	1.103(19)	1.104(19)	1.101(19)	1.106(19)	1.105(18)	1.103(18)
**C21**	**H21A**	1.16(2)	1(NA)	1.11(2)	1.11(2)	1.11(2)	1.12(2)	1.116(19)	1.114(19)	1.11(2)	1.11(2)	1.11(2)	1.11(2)	1.11(2)	1.11(2)
**C22**	**H22A**	1.13(2)	1(NA)	1.11(2)	1.11(2)	1.11(2)	1.109(19)	1.111(19)	1.111(19)	1.110(19)	1.11(2)	1.11(2)	1.11(2)	1.110(19)	1.112(19)
**C23**	**H23A**	1.12(3)	0.98(NA)	1.11(2)	1.11(2)	1.11(2)	1.11(2)	1.11(2)	1.11(2)	1.11(2)	1.11(2)	1.11(2)	1.11(2)	1.11(2)	1.11(2)
**C23**	**H23B**	1.06(4)	0.98(NA)	1.13(2)	1.13(2)	1.13(2)	1.13(2)	1.13(2)	1.12(2)	1.11(2)	1.11(2)	1.11(3)	1.11(3)	1.11(2)	1.11(2)
**C23**	**H23C**	1.08(3)	0.98(NA)	1.11(3)	1.11(3)	1.11(3)	1.11(2)	1.11(2)	1.11(2)	1.13(2)	1.13(2)	1.13(2)	1.13(2)	1.13(2)	1.13(2)
**C24**	**H24A**	1.06(3)	0.98(NA)	1.21(2)	1.20(2)	1.20(2)	1.20(2)	1.20(2)	1.19(2)	1.21(2)	1.21(2)	1.21(2)	1.21(2)	1.20(2)	1.20(2)
**C24**	**H24B**	1.12(3)	0.98(NA)	1.08(2)	1.08(2)	1.07(2)	1.08(2)	1.08(2)	1.08(2)	1.08(2)	1.08(2)	1.08(2)	1.08(2)	1.08(2)	1.08(2)
**C24**	**H24C**	1.08(3)	0.98(NA)	1.08(2)	1.08(2)	1.08(2)	1.08(2)	1.08(2)	1.08(2)	1.08(2)	1.08(2)	1.07(2)	1.08(2)	1.08(2)	1.08(2)
**C25**	**H25A**	1.14(2)	0.98(NA)	1.12(2)	1.12(2)	1.12(2)	1.12(2)	1.12(2)	1.12(2)	1.12(2)	1.12(2)	1.12(2)	1.13(2)	1.13(2)	1.12(2)
**C25**	**H25B**	1.10(3)	0.98(NA)	1.11(3)	1.11(3)	1.11(3)	1.11(2)	1.11(2)	1.11(2)	1.095(19)	1.095(19)	1.10(2)	1.094(19)	1.093(19)	1.094(19)
**C25**	**H25C**	1.11(3)	0.98(NA)	1.09(2)	1.10(2)	1.09(2)	1.095(19)	1.096(19)	1.094(19)	1.11(2)	1.11(2)	1.11(2)	1.11(2)	1.11(2)	1.11(2)
**C26**	**H26A**	1.06(3)	0.98(NA)	1.09(2)	1.09(2)	1.09(2)	1.09(2)	1.09(2)	1.09(2)	1.09(2)	1.09(2)	1.09(2)	1.09(2)	1.09(2)	1.09(2)
**C26**	**H26B**	1.14(3)	0.98(NA)	1.17(3)	1.16(3)	1.16(3)	1.16(2)	1.15(2)	1.15(2)	1.09(2)	1.09(2)	1.09(2)	1.09(2)	1.09(2)	1.09(2)
**C26**	**H26C**	1.13(3)	0.98(NA)	1.09(2)	1.09(2)	1.09(2)	1.09(2)	1.09(2)	1.09(2)	1.16(2)	1.16(2)	1.16(3)	1.16(2)	1.16(2)	1.16(2)
**C28**	**H28A**	1.12(2)	0.95(NA)	1.137(19)	1.139(19)	1.139(19)	1.134(18)	1.138(18)	1.136(18)	1.137(18)	1.137(18)	1.143(19)	1.138(18)	1.136(18)	1.140(18)
**C29**	**H29A**	1.10(3)	0.95(NA)	1.08(2)	1.08(2)	1.08(2)	1.07(2)	1.08(2)	1.08(2)	1.07(2)	1.07(2)	1.08(2)	1.08(2)	1.07(2)	1.08(2)
**C30**	**H30A**	1.13(2)	0.95(NA)	1.11(2)	1.11(2)	1.11(2)	1.11(2)	1.11(2)	1.11(2)	1.11(2)	1.11(2)	1.11(2)	1.11(2)	1.11(2)	1.11(2)
**C31**	**H31A**	1.12(2)	0.95(NA)	1.053(19)	1.056(19)	1.054(19)	1.057(18)	1.059(18)	1.058(18)	1.056(18)	1.056(18)	1.057(19)	1.057(18)	1.057(18)	1.058(18)
**C33**	**H33A**	1.16(2)	0.99(NA)	1.109(17)	1.109(17)	1.107(17)	1.112(16)	1.112(16)	1.110(16)	1.110(17)	1.110(17)	1.107(17)	1.111(17)	1.109(17)	1.109(17)
**C33**	**H33B**	1.12(2)	0.99(NA)	1.100(18)	1.101(18)	1.099(18)	1.102(17)	1.102(17)	1.100(17)	1.099(17)	1.099(17)	1.097(18)	1.102(17)	1.101(17)	1.100(17)
**C34**	**H34A**	1.13(2)	1(NA)	1.089(19)	1.089(19)	1.086(19)	1.095(18)	1.094(18)	1.092(18)	1.091(18)	1.090(18)	1.087(19)	1.094(18)	1.091(18)	1.091(18)
**C35**	**H35A**	1.13(2)	1(NA)	1.11(2)	1.11(2)	1.11(2)	1.11(2)	1.11(2)	1.10(2)	1.11(2)	1.11(2)	1.10(2)	1.11(2)	1.11(2)	1.10(2)
**C36**	**H36A**	1.08(3)	0.98(NA)	1.06(2)	1.07(2)	1.06(2)	1.07(2)	1.07(2)	1.07(2)	1.07(2)	1.07(2)	1.06(2)	1.07(2)	1.07(2)	1.07(2)
**C36**	**H36B**	1.15(3)	0.98(NA)	1.15(3)	1.15(3)	1.15(3)	1.15(2)	1.15(2)	1.15(2)	1.10(2)	1.10(2)	1.10(2)	1.10(2)	1.10(2)	1.10(2)
**C36**	**H36C**	1.08(3)	0.98(NA)	1.10(2)	1.11(2)	1.10(2)	1.10(2)	1.10(2)	1.10(2)	1.14(2)	1.14(2)	1.15(3)	1.15(3)	1.15(2)	1.15(2)
**C37**	**H37A**	1.10(3)	0.98(NA)	1.11(3)	1.11(3)	1.11(3)	1.10(2)	1.10(2)	1.10(3)	1.10(3)	1.10(3)	1.11(3)	1.10(3)	1.10(3)	1.10(3)
**C37**	**H37B**	1.09(3)	0.98(NA)	1.07(2)	1.07(2)	1.07(2)	1.07(2)	1.07(2)	1.07(2)	1.07(2)	1.07(2)	1.07(2)	1.07(2)	1.08(2)	1.08(2)
**C37**	**H37C**	1.09(3)	0.98(NA)	1.07(3)	1.08(2)	1.07(2)	1.08(2)	1.08(2)	1.08(2)	1.07(2)	1.07(2)	1.07(2)	1.07(2)	1.07(2)	1.07(2)
**C38**	**H38A**	1.14(2)	0.98(NA)	1.10(2)	1.11(2)	1.11(2)	1.09(2)	1.10(2)	1.10(2)	1.10(2)	1.10(2)	1.10(2)	1.10(2)	1.10(2)	1.10(2)
**C38**	**H38B**	1.10(3)	0.98(NA)	1.12(2)	1.12(2)	1.12(2)	1.125(19)	1.128(19)	1.127(19)	1.12(2)	1.12(2)	1.12(2)	1.12(2)	1.12(2)	1.12(2)
**C38**	**H38C**	1.11(3)	0.98(NA)	1.12(2)	1.11(2)	1.11(2)	1.12(2)	1.11(2)	1.11(2)	1.12(2)	1.12(2)	1.13(2)	1.13(2)	1.13(2)	1.13(2)
**C39**	**H39A**	1.08(3)	0.98(NA)	1.09(3)	1.10(3)	1.10(3)	1.10(3)	1.10(3)	1.10(3)	1.10(3)	1.10(3)	1.10(3)	1.09(3)	1.09(3)	1.10(3)

**C39**	**H39B**	1.08(3)	0.98(NA)	1.13(2)	1.13(2)	1.13(2)	1.13(2)	1.13(2)	1.13(2)	1.08(2)	1.08(2)	1.08(3)	1.08(2)	1.08(2)	1.08(2)
**C39**	**H39C**	1.12(3)	0.98(NA)	1.08(3)	1.08(3)	1.07(3)	1.08(2)	1.08(2)	1.08(2)	1.13(2)	1.13(2)	1.13(2)	1.13(2)	1.13(2)	1.13(2)

Table S23 X-H bond lengths (units: Å) obtained for GOJNIF with various experimental methods (neutron and X-ray) and refinement methods (IAM and HAR). HAR was performed with a cluster of charges and dipoles modeling crystal environment (DiSCaMB) and without a cluster (NoSpherA2). DFT calculations for HAR were performed with various functionals in the non-relativistic version (B3LYP, PBE and M06-2X) and with relativistic correction (B3LYP-DKH2, PBE-DKH2 and M06-2X-DKH2). Basis sets used: (a) non-relativistic refinements: jorge-TZP, (b) relativistic refinements: jorge-TZP-DKH.

				DiSCaMB-HAR						NoSpherA2-HAR					
**bond**		**neutron**	**IAM**	**B3LYP**	**PBE**	**M06-2X**	**B3LYP-DKH2**	**PBE-DKH2**	**M06-2X-DKH2**	**B3LYP**	**PBE**	**M06-2X**	**B3LYP-DKH2**	**PBE-DKH2**	**M06-2X-DKH2**
**Ni1**	**H1A**	1.61(2)	1.64(2)	1.68(4)	1.67(4)	1.67(4)	1.68(4)	1.67(4)	1.68(4)	1.68(4)	1.65(4)	1.66(4)	1.68(4)	1.65(4)	1.66(4)
**Ni1**	**H1B**	1.61(2)	1.58(2)	1.71(4)	1.72(4)	1.71(4)	1.70(3)	1.71(3)	1.70(3)	1.70(3)	1.73(4)	1.72(4)	1.70(3)	1.73(4)	1.72(4)
**C2**	**H2A**	1.10(3)	0.95	1.055(19)	1.062(19)	1.059(19)	1.057(18)	1.064(18)	1.063(18)	1.059(18)	1.058(19)	1.063(19)	1.058(18)	1.057(18)	1.062(19)
**C3**	**H3A**	1.15(2)	0.95	1.10(2)	1.11(2)	1.10(2)	1.101(19)	1.101(19)	1.096(19)	1.10(2)	1.10(2)	1.10(2)	1.099(19)	1.10(2)	1.10(2)
**C4**	**H4A**	1.13(2)	0.95(NA)	1.09(2)	1.092(19)	1.090(19)	1.088(18)	1.092(18)	1.090(18)	1.087(19)	1.088(19)	1.09(2)	1.087(18)	1.089(19)	1.091(19)
**C5**	**H5A**	1.12(3)	0.95(NA)	1.130(19)	1.127(19)	1.125(19)	1.124(18)	1.121(17)	1.119(17)	1.126(18)	1.134(19)	1.131(19)	1.122(18)	1.130(18)	1.126(18)
**C7**	**H7A**	1.15(2)	0.99(NA)	1.116(19)	1.118(18)	1.117(19)	1.115(18)	1.116(17)	1.115(17)	1.116(18)	1.116(18)	1.118(19)	1.115(18)	1.115(18)	1.117(18)
**C7**	**H7B**	1.14(2)	0.99(NA)	1.09(2)	1.10(2)	1.09(2)	1.10(2)	1.10(2)	1.10(2)	1.09(2)	1.09(2)	1.09(2)	1.10(2)	1.10(2)	1.10(2)
**C8**	**H8A**	1.13(3)	1(NA)	1.13(2)	1.13(2)	1.13(2)	1.13(2)	1.13(2)	1.13(2)	1.13(2)	1.13(2)	1.13(2)	1.13(2)	1.13(2)	1.13(2)
**C9**	**H9A**	1.13(2)	1(NA)	1.14(2)	1.14(2)	1.14(2)	1.14(2)	1.14(2)	1.14(2)	1.14(2)	1.14(2)	1.14(2)	1.14(2)	1.14(2)	1.14(2)
**C10**	**H10A**	1.08(3)	0.98(NA)	1.11(3)	1.11(3)	1.11(3)	1.10(3)	1.11(3)	1.10(3)	1.10(3)	1.11(3)	1.11(3)	1.10(3)	1.10(3)	1.11(3)
**C10**	**H10B**	1.07(4)	0.98(NA)	1.11(2)	1.12(2)	1.12(2)	1.12(2)	1.12(2)	1.12(2)	1.12(2)	1.13(3)	1.12(3)	1.12(2)	1.13(2)	1.12(2)
**C10**	**H10C**	1.11(3)	0.98(NA)	1.12(3)	1.12(3)	1.12(3)	1.12(2)	1.12(2)	1.12(2)	1.12(2)	1.12(2)	1.12(2)	1.12(2)	1.12(2)	1.12(2)
**C11**	**H11A**	1.11(3)	0.98(NA)	1.11(3)	1.12(3)	1.11(3)	1.10(3)	1.11(3)	1.11(2)	1.11(3)	1.11(3)	1.11(3)	1.11(3)	1.11(3)	1.11(3)
**C11**	**H11B**	1.11(3)	0.98(NA)	1.11(3)	1.11(3)	1.11(3)	1.11(3)	1.11(3)	1.11(3)	1.04(3)	1.05(3)	1.05(3)	1.04(2)	1.04(3)	1.05(3)
**C11**	**H11C**	1.10(3)	0.98(NA)	1.04(3)	1.05(3)	1.05(3)	1.04(2)	1.05(2)	1.05(2)	1.11(3)	1.11(3)	1.11(3)	1.11(3)	1.11(3)	1.11(3)
**C12**	**H12A**	1.10(3)	0.98(NA)	1.10(2)	1.10(2)	1.10(2)	1.09(2)	1.10(2)	1.10(2)	1.10(2)	1.10(2)	1.10(2)	1.09(2)	1.10(2)	1.10(2)
**C12**	**H12B**	1.11(3)	0.98(NA)	1.11(3)	1.12(3)	1.11(3)	1.11(2)	1.12(2)	1.11(2)	1.13(2)	1.13(2)	1.13(2)	1.13(2)	1.13(2)	1.13(2)
**C12**	**H12C**	1.09(4)	0.98(NA)	1.13(2)	1.13(2)	1.13(2)	1.13(2)	1.13(2)	1.13(2)	1.11(2)	1.12(3)	1.12(3)	1.11(2)	1.12(2)	1.12(2)
**C13**	**H13A**	1.11(3)	0.98(NA)	1.11(3)	1.11(3)	1.11(3)	1.10(2)	1.10(2)	1.10(2)	1.10(2)	1.11(2)	1.11(3)	1.10(2)	1.11(2)	1.11(2)
**C13**	**H13B**	1.05(4)	0.98(NA)	1.12(2)	1.12(2)	1.12(2)	1.12(2)	1.12(2)	1.12(2)	1.12(2)	1.12(2)	1.12(2)	1.12(2)	1.12(2)	1.12(2)
**C13**	**H13C**	1.12(4)	0.98(NA)	1.12(2)	1.12(2)	1.12(2)	1.12(2)	1.12(2)	1.12(2)	1.13(2)	1.13(2)	1.13(2)	1.12(2)	1.13(2)	1.12(2)
**C15**	**H15A**	1.10(3)	0.95(NA)	1.13(2)	1.131(19)	1.128(19)	1.129(18)	1.128(18)	1.124(18)	1.130(19)	1.133(19)	1.13(2)	1.128(18)	1.131(19)	1.129(19)
**C16**	**H16A**	1.15(2)	0.95(NA)	1.11(2)	1.11(2)	1.11(2)	1.106(19)	1.110(19)	1.107(19)	1.11(2)	1.11(2)	1.11(2)	1.106(19)	1.109(19)	1.11(2)
**C17**	**H17A**	1.13(2)	0.95(NA)	1.11(2)	1.11(2)	1.11(2)	1.11(2)	1.11(2)	1.11(2)	1.11(2)	1.11(2)	1.11(2)	1.10(2)	1.11(2)	1.11(2)
**C18**	**H18A**	1.13(3)	0.95(NA)	1.11(2)	1.11(2)	1.10(2)	1.106(19)	1.106(19)	1.105(19)	1.11(2)	1.10(2)	1.11(2)	1.106(19)	1.104(19)	1.104(19)

**C20**	**H20A**	1.12(2)	0.99(NA)	1.15(2)	1.15(2)	1.15(2)	1.14(2)	1.146(19)	1.145(19)	1.15(2)	1.15(2)	1.15(2)	1.14(2)	1.15(2)	1.15(2)
**C20**	**H20B**	1.13(2)	0.99(NA)	1.104(19)	1.105(19)	1.102(19)	1.106(18)	1.107(17)	1.104(17)	1.105(18)	1.105(19)	1.102(19)	1.106(18)	1.107(18)	1.104(19)
**C21**	**H21A**	1.16(2)	1(NA)	1.11(2)	1.11(2)	1.11(2)	1.115(19)	1.117(19)	1.114(19)	1.11(2)	1.11(2)	1.11(2)	1.115(19)	1.12(2)	1.11(2)
**C22**	**H22A**	1.13(2)	1(NA)	1.11(2)	1.11(2)	1.11(2)	1.108(19)	1.111(18)	1.109(18)	1.111(19)	1.11(2)	1.11(2)	1.108(19)	1.110(19)	1.111(19)
**C23**	**H23A**	1.12(3)	0.98(NA)	1.11(2)	1.11(2)	1.11(2)	1.11(2)	1.11(2)	1.11(2)	1.11(2)	1.10(2)	1.11(2)	1.11(2)	1.10(2)	1.11(2)
**C23**	**H23B**	1.06(4)	0.98(NA)	1.14(2)	1.14(2)	1.13(2)	1.13(2)	1.13(2)	1.12(2)	1.10(2)	1.10(2)	1.10(3)	1.10(2)	1.10(2)	1.10(2)
**C23**	**H23C**	1.08(3)	0.98(NA)	1.10(3)	1.11(2)	1.10(3)	1.10(2)	1.10(2)	1.10(2)	1.13(2)	1.13(2)	1.13(2)	1.13(2)	1.13(2)	1.13(2)
**C24**	**H24A**	1.06(3)	0.98(NA)	1.21(2)	1.20(2)	1.20(2)	1.20(2)	1.20(2)	1.19(2)	1.21(2)	1.21(2)	1.21(2)	1.20(2)	1.20(2)	1.20(2)
**C24**	**H24B**	1.12(3)	0.98(NA)	1.08(2)	1.08(2)	1.08(2)	1.08(2)	1.09(2)	1.08(2)	1.08(2)	1.08(2)	1.08(2)	1.08(2)	1.08(2)	1.08(2)
**C24**	**H24C**	1.08(3)	0.98(NA)	1.08(2)	1.08(2)	1.08(2)	1.08(2)	1.08(2)	1.08(2)	1.08(2)	1.08(2)	1.07(2)	1.08(2)	1.08(2)	1.08(2)
**C25**	**H25A**	1.14(2)	0.98(NA)	1.12(2)	1.12(2)	1.12(2)	1.12(2)	1.12(2)	1.12(2)	1.12(2)	1.13(2)	1.12(2)	1.12(2)	1.13(2)	1.12(2)
**C25**	**H25B**	1.10(3)	0.98(NA)	1.11(3)	1.11(2)	1.11(2)	1.10(2)	1.11(2)	1.10(2)	1.096(19)	1.10(2)	1.10(2)	1.094(18)	1.094(19)	1.093(19)
**C25**	**H25C**	1.11(3)	0.98(NA)	1.09(2)	1.096(19)	1.09(2)	1.094(18)	1.096(18)	1.094(18)	1.11(2)	1.11(2)	1.11(2)	1.10(2)	1.11(2)	1.11(2)
**C26**	**H26A**	1.06(3)	0.98(NA)	1.09(2)	1.09(2)	1.09(2)	1.09(2)	1.09(2)	1.09(2)	1.09(2)	1.09(2)	1.09(2)	1.09(2)	1.09(2)	1.09(2)
**C26**	**H26B**	1.14(3)	0.98(NA)	1.16(2)	1.15(2)	1.15(2)	1.15(2)	1.14(2)	1.14(2)	1.10(2)	1.10(2)	1.09(2)	1.09(2)	1.10(2)	1.09(2)
**C26**	**H26C**	1.13(3)	0.98(NA)	1.09(3)	1.09(2)	1.09(2)	1.09(2)	1.09(2)	1.09(2)	1.15(2)	1.15(2)	1.15(2)	1.14(2)	1.15(2)	1.15(2)
**C28**	**H28A**	1.12(2)	0.95(NA)	1.127(19)	1.131(18)	1.129(19)	1.125(18)	1.130(17)	1.127(17)	1.127(18)	1.130(19)	1.134(19)	1.124(18)	1.126(18)	1.131(18)
**C29**	**H29A**	1.10(3)	0.95(NA)	1.07(2)	1.08(2)	1.08(2)	1.07(2)	1.07(2)	1.07(2)	1.07(2)	1.07(2)	1.07(2)	1.07(2)	1.07(2)	1.07(2)
**C30**	**H30A**	1.13(2)	0.95(NA)	1.11(2)	1.11(2)	1.11(2)	1.11(2)	1.11(2)	1.10(2)	1.11(2)	1.11(2)	1.11(2)	1.11(2)	1.11(2)	1.11(2)
**C31**	**H31A**	1.12(2)	0.95(NA)	1.056(19)	1.059(19)	1.056(19)	1.060(18)	1.062(18)	1.060(18)	1.058(18)	1.059(19)	1.058(19)	1.059(18)	1.060(18)	1.059(18)
**C33**	**H33A**	1.16(2)	0.99(NA)	1.108(17)	1.110(17)	1.107(17)	1.111(16)	1.112(16)	1.109(16)	1.109(17)	1.107(17)	1.106(17)	1.111(16)	1.109(16)	1.109(17)
**C33**	**H33B**	1.12(2)	0.99(NA)	1.101(18)	1.102(18)	1.100(18)	1.102(17)	1.103(16)	1.100(16)	1.100(17)	1.099(17)	1.098(18)	1.102(17)	1.101(17)	1.100(17)
**C34**	**H34A**	1.13(2)	1(NA)	1.094(19)	1.093(19)	1.090(19)	1.098(18)	1.097(17)	1.094(17)	1.096(18)	1.093(19)	1.091(19)	1.098(18)	1.095(18)	1.093(18)
**C35**	**H35A**	1.13(2)	1(NA)	1.11(2)	1.11(2)	1.11(2)	1.11(2)	1.108(19)	1.105(19)	1.11(2)	1.11(2)	1.11(2)	1.11(2)	1.11(2)	1.11(2)
**C36**	**H36A**	1.08(3)	0.98(NA)	1.07(2)	1.07(2)	1.07(2)	1.07(2)	1.08(2)	1.07(2)	1.07(2)	1.07(2)	1.07(2)	1.07(2)	1.07(2)	1.07(2)
**C36**	**H36B**	1.15(3)	0.98(NA)	1.14(3)	1.15(2)	1.14(2)	1.14(2)	1.14(2)	1.14(2)	1.10(2)	1.11(2)	1.10(2)	1.10(2)	1.11(2)	1.10(2)
**C36**	**H36C**	1.08(3)	0.98(NA)	1.10(2)	1.11(2)	1.10(2)	1.10(2)	1.106(19)	1.103(19)	1.14(2)	1.15(3)	1.14(3)	1.14(2)	1.15(2)	1.14(2)
**C37**	**H37A**	1.10(3)	0.98(NA)	1.10(3)	1.11(3)	1.11(3)	1.10(2)	1.10(2)	1.10(2)	1.10(3)	1.10(3)	1.11(3)	1.10(2)	1.10(2)	1.10(3)
**C37**	**H37B**	1.09(3)	0.98(NA)	1.07(2)	1.07(2)	1.07(2)	1.07(2)	1.07(2)	1.07(2)	1.07(2)	1.07(2)	1.07(2)	1.08(2)	1.08(2)	1.08(2)
**C37**	**H37C**	1.09(3)	0.98(NA)	1.07(3)	1.08(2)	1.08(2)	1.08(2)	1.08(2)	1.08(2)	1.07(2)	1.07(2)	1.07(2)	1.07(2)	1.07(2)	1.07(2)
**C38**	**H38A**	1.14(2)	0.98(NA)	1.10(2)	1.11(2)	1.11(2)	1.10(2)	1.10(2)	1.10(2)	1.10(2)	1.10(2)	1.10(2)	1.09(2)	1.10(2)	1.10(2)
**C38**	**H38B**	1.10(3)	0.98(NA)	1.12(2)	1.12(2)	1.12(2)	1.121(19)	1.125(19)	1.121(19)	1.12(2)	1.13(2)	1.12(2)	1.12(2)	1.13(2)	1.12(2)
**C38**	**H38C**	1.11(3)	0.98(NA)	1.12(2)	1.12(2)	1.12(2)	1.12(2)	1.12(2)	1.12(2)	1.12(2)	1.12(2)	1.12(2)	1.121(19)	1.12(2)	1.12(2)
**C39**	**H39A**	1.08(3)	0.98(NA)	1.10(3)	1.10(3)	1.10(3)	1.10(3)	1.10(3)	1.10(3)	1.10(3)	1.09(3)	1.10(3)	1.10(3)	1.10(3)	1.10(3)

**C39**	**H39B**	1.08(3)	0.98(NA)	1.13(2)	1.13(2)	1.13(2)	1.13(2)	1.13(2)	1.13(2)	1.08(2)	1.09(3)	1.08(3)	1.08(2)	1.09(2)	1.08(2)
**C39**	**H39C**	1.12(3)	0.98(NA)	1.08(3)	1.08(2)	1.08(3)	1.08(2)	1.08(2)	1.08(2)	1.13(2)	1.13(2)	1.13(2)	1.13(2)	1.13(2)	1.13(2)

Table S24 X-H bond lengths (units: Å) obtained for TIWXOP with various experimental methods (neutron and X-ray) and refinement methods (IAM and HAR). HAR was performed with a cluster of charges and dipoles modeling crystal environment (DiSCaMB) and without a cluster (NoSpherA2). DFT calculations for HAR were performed with various functionals in the non-relativistic version (B3LYP, PBE and M06-2X) and with relativistic correction (B3LYP-DKH2, PBE-DKH2 and M06-2X-DKH2). Basis sets used: (a) non-relativistic refinements: cc-pVTZ-DK, (b) relativistic refinements: cc-pVTZ-DK.

				**DiSCaMB-HAR**						**NoSpherA2-HAR**					
**bond**		**neutron**	**IAM**	**B3LYP**	**PBE**	**M06-2X**	**B3LYP-DKH2**	**PBE-DKH2**	**M06-2X-DKH2**	**B3LYP**	**PBE**	**M06-2X**	**B3LYP-DKH2**	**PBE-DKH2**	**M06-2X-DKH2**
**Sb**	**H1A**	1.73(2)	1.74(3)	1.81(2)	1.81(2)	1.81(2)	1.80(2)	1.81(2)	1.80(2)	1.82(3)	1.81(3)	1.82(3)	1.81(3)	1.81(3)	1.81(3)
**C3**	**H3**	1.11(3)	0.88(3)	1.08(3)	1.08(3)	1.07(3)	1.07(3)	1.07(3)	1.03(3)	1.07(3)	1.07(3)	1.07(3)	1.08(3)	1.07(3)	1.07(3)
**C4**	**H4**	1.10(3)	0.96(3)	1.14(3)	1.14(3)	1.13(3)	1.13(3)	1.13(3)	1.11(3)	1.13(3)	1.13(3)	1.13(3)	1.13(3)	1.13(3)	1.13(3)
**C5**	**H5**	1.03(3)	0.92(3)	1.04(2)	1.04(2)	1.04(2)	1.03(2)	1.03(2)	1.00(2)	1.04(2)	1.04(2)	1.05(2)	1.03(2)	1.04(2)	1.03(2)
**C7**	**H7A**	1.10(3)	0.92(3)	1.06(3)	1.07(3)	1.06(3)	1.06(3)	1.06(3)	1.05(3)	1.06(3)	1.06(3)	1.07(3)	1.06(3)	1.06(3)	1.07(3)
**C7**	**H7B**	1.08(4)	0.90(4)	1.04(4)	1.05(4)	1.04(4)	1.04(4)	1.04(4)	1.02(4)	1.04(4)	1.05(4)	1.04(4)	1.05(4)	1.05(4)	1.05(4)
**C7**	**H7C**	1.04(5)	1.01(4)	1.10(4)	1.10(4)	1.10(4)	1.09(4)	1.09(4)	1.08(4)	1.08(4)	1.09(4)	1.07(4)	1.09(4)	1.10(4)	1.09(4)
**C8**	**H8A**	1.08(NA)	0.85(5)	0.99(5)	1.00(5)	0.99(5)	0.97(5)	0.98(5)	0.91(5)	0.99(6)	0.99(6)	0.99(6)	0.99(5)	0.99(6)	0.98(5)
**C8**	**H8B**	1.08(NA)	0.90(4)	1.10(4)	1.10(4)	1.10(4)	1.08(4)	1.08(4)	1.05(4)	1.09(4)	1.09(4)	1.10(4)	1.09(4)	1.08(4)	1.09(4)
**C8**	**H8C**	1.08(NA)	0.96(4)	1.07(4)	1.07(4)	1.07(4)	1.07(4)	1.07(3)	1.07(4)	1.07(4)	1.07(4)	1.07(4)	1.06(4)	1.06(4)	1.06(4)
**C9**	**H9A**	1.06(3)	0.95(4)	1.08(3)	1.08(3)	1.08(3)	1.08(3)	1.08(3)	1.08(3)	1.09(3)	1.09(3)	1.09(3)	1.08(3)	1.08(3)	1.08(3)
**C9**	**H9B**	1.09(6)	0.96(4)	1.11(4)	1.12(4)	1.12(4)	1.11(4)	1.11(4)	1.09(4)	1.12(4)	1.12(4)	1.13(4)	1.12(4)	1.12(4)	1.12(4)
**C9**	**H9C**	1.09(4)	0.95(4)	1.09(4)	1.09(4)	1.09(4)	1.08(4)	1.08(4)	1.07(4)	1.08(4)	1.08(4)	1.07(4)	1.08(4)	1.08(4)	1.08(4)
**C10**	**H10A**	1.07(3)	0.90(4)	1.05(4)	1.06(4)	1.05(4)	1.05(4)	1.05(3)	1.06(4)	1.06(4)	1.05(4)	1.06(4)	1.05(4)	1.05(4)	1.05(4)
**C10**	**H10B**	1.08(4)	1.04(5)	1.18(4)	1.18(4)	1.18(4)	1.17(4)	1.18(4)	1.16(4)	1.17(5)	1.18(5)	1.17(5)	1.18(4)	1.18(4)	1.18(4)
**C10**	**H10C**	0.97(4)	0.89(4)	1.04(3)	1.04(3)	1.04(3)	1.03(3)	1.03(3)	1.01(3)	1.05(4)	1.05(4)	1.04(4)	1.04(4)	1.04(4)	1.04(4)
**C11**	**H11A**	1.07(4)	0.94(3)	1.11(3)	1.11(3)	1.11(3)	1.09(3)	1.09(3)	1.08(3)	1.10(3)	1.10(3)	1.10(3)	1.10(3)	1.10(3)	1.10(3)
**C11**	**H11B**	1.05(4)	0.95(3)	1.06(3)	1.06(3)	1.06(3)	1.05(3)	1.06(3)	1.04(3)	1.06(3)	1.06(4)	1.06(4)	1.06(3)	1.06(3)	1.06(3)
**C11**	**H11C**	1.11(3)	0.95(3)	1.08(3)	1.08(3)	1.07(3)	1.08(3)	1.08(3)	1.07(3)	1.08(3)	1.08(3)	1.08(3)	1.08(3)	1.08(3)	1.08(3)
**C12**	**H12A**	1.05(4)	0.91(4)	1.09(3)	1.09(3)	1.09(3)	1.08(3)	1.08(3)	1.05(3)	1.09(3)	1.09(3)	1.08(3)	1.09(3)	1.09(3)	1.09(3)
**C12**	**H12B**	1.01(6)	0.93(4)	1.09(4)	1.09(4)	1.08(4)	1.09(4)	1.09(4)	1.07(4)	1.09(4)	1.09(4)	1.09(4)	1.10(4)	1.10(4)	1.09(4)
**C12**	**H12C**	1.02(5)	0.89(4)	1.05(4)	1.05(4)	1.05(4)	1.05(4)	1.05(4)	1.04(4)	1.05(4)	1.05(4)	1.04(4)	1.05(4)	1.04(4)	1.05(4)
**C15**	**H15**	1.06(4)	0.92(3)	1.07(3)	1.07(3)	1.07(3)	1.07(3)	1.07(3)	1.06(3)	1.08(3)	1.08(3)	1.08(3)	1.08(3)	1.08(3)	1.07(3)
**C16**	**H16**	1.07(4)	0.93(3)	1.09(3)	1.10(3)	1.09(3)	1.08(3)	1.09(3)	1.07(3)	1.08(3)	1.08(3)	1.08(3)	1.09(3)	1.09(3)	1.09(3)
**C17**	**H17**	1.07(3)	0.94(3)	1.08(3)	1.08(3)	1.08(3)	1.07(3)	1.07(3)	1.05(3)	1.08(3)	1.08(3)	1.08(3)	1.07(3)	1.08(3)	1.07(3)
**C19**	**H19A**	1.08(4)	0.96(3)	1.08(3)	1.09(3)	1.09(3)	1.07(3)	1.08(3)	1.05(3)	1.09(4)	1.08(4)	1.09(3)	1.08(3)	1.08(3)	1.08(3)

**C19**	**H19B**	1.10(4)	0.95(3)	1.10(3)	1.10(3)	1.10(3)	1.09(3)	1.09(3)	1.07(3)	1.10(3)	1.10(3)	1.11(3)	1.10(3)	1.10(3)	1.10(3)
**C19**	**H19C**	1.01(5)	0.92(4)	1.06(4)	1.06(4)	1.06(4)	1.05(4)	1.05(4)	1.03(4)	1.04(4)	1.05(4)	1.02(4)	1.05(4)	1.06(4)	1.05(4)
**C20**	**H20A**	1.15(4)	0.96(4)	1.10(4)	1.10(4)	1.09(4)	1.08(4)	1.09(4)	1.07(4)	1.09(4)	1.09(4)	1.09(4)	1.09(4)	1.09(4)	1.09(4)
**C20**	**H20B**	1.02(5)	0.92(4)	1.09(4)	1.09(4)	1.08(4)	1.07(4)	1.07(4)	1.04(4)	1.08(4)	1.08(4)	1.08(4)	1.08(4)	1.08(4)	1.08(4)
**C20**	**H20C**	1.11(4)	0.91(4)	1.04(4)	1.05(4)	1.04(4)	1.04(4)	1.04(4)	1.02(4)	1.04(4)	1.04(4)	1.04(5)	1.04(4)	1.05(4)	1.04(4)

Table S25 X-H bond lengths (units: Å) obtained for TIWXOP with various experimental methods (neutron and X-ray) and refinement methods (IAM and HAR). HAR was performed with a cluster of charges and dipoles modeling crystal environment (DiSCaMB) and without a cluster (NoSpherA2). DFT calculations for HAR were performed with various functionals in the non-relativistic version (B3LYP, PBE and M06-2X) and with relativistic correction (B3LYP-DKH2, PBE-DKH2 and M06-2X-DKH2). Basis sets used: (a) non-relativistic refinements: jorge-DZP, (b) relativistic refinements: jorge-DZP-DKH.

				DiSCaMB-HAR						NoSpherA2-HAR					
**bond**		**neutron**	**IAM**	**B3LYP**	**PBE**	**M06-2X**	**B3LYP-DKH2**	**PBE-DKH2**	**M06-2X-DKH2**	**B3LYP**	**PBE**	**M06-2X**	**B3LYP-DKH2**	**PBE-DKH2**	**M06-2X-DKH2**
**Sb**	**H1A**	1.73(2)	1.74(3)	1.82(2)	1.82(2)	1.81(2)	1.81(2)	1.81(2)	1.81(2)	1.81(2)	1.76(3)	1.81(2)	1.81(2)	1.76(3)	1.81(2)
**C3**	**H3**	1.11(3)	0.88(3)	1.08(3)	1.08(3)	1.08(3)	1.08(3)	1.08(3)	1.08(3)	1.09(3)	0.95(3)	1.09(3)	1.09(3)	0.94(3)	1.08(3)
**C4**	**H4**	1.10(3)	0.96(3)	1.14(3)	1.14(3)	1.14(3)	1.13(3)	1.13(3)	1.13(3)	1.14(3)	1.05(3)	1.14(3)	1.14(3)	1.04(3)	1.13(3)
**C5**	**H5**	1.03(3)	0.92(3)	1.04(2)	1.04(2)	1.04(2)	1.04(2)	1.04(2)	1.04(2)	1.05(2)	0.99(3)	1.05(2)	1.04(2)	0.98(3)	1.04(2)
**C7**	**H7A**	1.10(3)	0.92(3)	1.06(3)	1.07(3)	1.06(3)	1.06(3)	1.06(3)	1.06(3)	1.06(3)	0.97(4)	1.07(3)	1.06(3)	0.97(4)	1.06(3)
**C7**	**H7B**	1.08(4)	0.90(4)	1.05(4)	1.05(4)	1.04(4)	1.05(4)	1.05(4)	1.05(4)	1.05(4)	1.00(4)	1.05(4)	1.05(4)	1.00(4)	1.05(4)
**C7**	**H7C**	1.04(5)	1.01(4)	1.12(4)	1.12(4)	1.12(4)	1.11(4)	1.11(4)	1.11(4)	1.12(4)	1.07(4)	1.12(4)	1.12(4)	1.07(4)	1.11(4)
**C8**	**H8A**	1.08(NA)	0.85(5)	0.99(5)	0.99(5)	0.98(5)	0.98(5)	0.98(5)	0.98(5)	1.01(6)	0.95(6)	1.00(5)	1.00(5)	0.94(6)	0.99(5)
**C8**	**H8B**	1.08(NA)	0.90(4)	1.09(4)	1.10(4)	1.09(4)	1.09(4)	1.09(4)	1.09(4)	1.09(4)	0.97(4)	1.10(4)	1.09(4)	0.97(4)	1.09(4)
**C8**	**H8C**	1.08(NA)	0.96(4)	1.07(4)	1.08(3)	1.07(3)	1.07(4)	1.07(3)	1.07(3)	1.06(4)	1.03(4)	1.07(4)	1.06(4)	1.03(4)	1.07(4)
**C9**	**H9A**	1.06(3)	0.95(4)	1.08(3)	1.08(3)	1.08(3)	1.08(3)	1.09(3)	1.08(3)	1.09(3)	1.02(4)	1.09(3)	1.09(3)	1.02(4)	1.09(3)
**C9**	**H9B**	1.09(6)	0.96(4)	1.11(4)	1.11(4)	1.11(4)	1.11(4)	1.11(4)	1.11(4)	1.12(4)	0.98(5)	1.12(4)	1.12(4)	0.98(5)	1.12(4)
**C9**	**H9C**	1.09(4)	0.95(4)	1.09(4)	1.09(4)	1.09(4)	1.09(4)	1.09(4)	1.09(4)	1.09(4)	0.96(4)	1.09(4)	1.09(4)	0.96(4)	1.09(4)
**C10**	**H10A**	1.07(3)	0.90(4)	1.06(4)	1.06(4)	1.06(4)	1.06(4)	1.06(3)	1.06(3)	1.06(4)	0.96(4)	1.06(4)	1.06(4)	0.96(4)	1.06(4)
**C10**	**H10B**	1.08(4)	1.04(5)	1.19(4)	1.20(4)	1.20(4)	1.19(4)	1.19(4)	1.19(4)	1.19(4)	1.07(5)	1.20(4)	1.19(4)	1.06(5)	1.19(4)
**C10**	**H10C**	0.97(4)	0.89(4)	1.04(3)	1.03(3)	1.03(3)	1.03(3)	1.03(3)	1.03(3)	1.05(4)	0.92(4)	1.04(4)	1.04(4)	0.92(4)	1.04(4)
**C11**	**H11A**	1.07(4)	0.94(3)	1.10(3)	1.10(3)	1.10(3)	1.09(3)	1.09(3)	1.09(3)	1.10(3)	0.99(3)	1.10(3)	1.10(3)	0.99(3)	1.10(3)
**C11**	**H11B**	1.05(4)	0.95(3)	1.07(3)	1.07(3)	1.07(3)	1.06(3)	1.07(3)	1.06(3)	1.08(3)	1.00(4)	1.08(3)	1.07(3)	0.99(4)	1.07(3)
**C11**	**H11C**	1.11(3)	0.95(3)	1.08(3)	1.08(3)	1.08(3)	1.08(3)	1.08(3)	1.08(3)	1.08(3)	1.00(4)	1.08(3)	1.08(3)	0.99(4)	1.08(3)
**C12**	**H12A**	1.05(4)	0.91(4)	1.10(3)	1.10(3)	1.10(3)	1.09(3)	1.09(3)	1.09(3)	1.10(3)	0.97(4)	1.10(3)	1.10(3)	0.97(4)	1.10(3)
**C12**	**H12B**	1.01(6)	0.93(4)	1.10(4)	1.10(4)	1.10(4)	1.10(4)	1.10(4)	1.10(4)	1.11(4)	1.03(4)	1.11(4)	1.11(4)	1.02(4)	1.11(4)
**C12**	**H12C**	1.02(5)	0.89(4)	1.04(4)	1.04(4)	1.04(4)	1.04(4)	1.04(4)	1.04(4)	1.04(4)	0.93(4)	1.04(4)	1.04(4)	0.93(4)	1.04(4)
**C15**	**H15**	1.06(4)	0.92(3)	1.08(3)	1.08(3)	1.08(3)	1.08(3)	1.08(3)	1.08(3)	1.08(3)	1.00(3)	1.08(3)	1.08(3)	1.00(3)	1.08(3)
**C16**	**H16**	1.07(4)	0.93(3)	1.10(3)	1.11(3)	1.10(3)	1.10(3)	1.10(3)	1.09(3)	1.10(3)	0.99(3)	1.10(3)	1.10(3)	0.99(3)	1.10(3)
**C17**	**H17**	1.07(3)	0.94(3)	1.08(3)	1.08(3)	1.08(3)	1.08(3)	1.08(3)	1.08(3)	1.08(3)	0.99(3)	1.08(3)	1.08(3)	0.99(3)	1.08(3)
**C19**	**H19A**	1.08(4)	0.96(3)	1.07(3)	1.08(3)	1.08(3)	1.07(3)	1.08(3)	1.08(3)	1.08(4)	1.03(4)	1.08(3)	1.08(4)	1.03(4)	1.08(3)

**C19**	**H19B**	1.10(4)	0.95(3)	1.10(3)	1.10(3)	1.10(3)	1.10(3)	1.10(3)	1.10(3)	1.10(3)	1.00(4)	1.10(3)	1.10(3)	1.00(4)	1.10(3)
**C19**	**H19C**	1.01(5)	0.92(4)	1.08(4)	1.08(4)	1.07(4)	1.07(4)	1.07(4)	1.07(4)	1.08(4)	0.98(4)	1.07(4)	1.07(4)	0.98(4)	1.07(4)
**C20**	**H20A**	1.15(4)	0.96(4)	1.10(4)	1.10(4)	1.10(4)	1.09(4)	1.09(4)	1.09(4)	1.10(4)	1.01(4)	1.10(4)	1.10(4)	1.01(4)	1.10(4)
**C20**	**H20B**	1.02(5)	0.92(4)	1.09(4)	1.09(4)	1.09(4)	1.08(4)	1.09(4)	1.08(4)	1.09(4)	0.99(4)	1.09(4)	1.09(4)	0.99(4)	1.09(4)
**C20**	**H20C**	1.11(4)	0.91(4)	1.04(4)	1.04(4)	1.04(4)	1.04(4)	1.04(4)	1.04(4)	1.05(4)	0.98(5)	1.05(4)	1.05(4)	0.98(5)	1.05(4)

Table S26 X-H bond lengths (units: Å) obtained for GOJNIF with various experimental methods (neutron and X-ray) and refinement methods (IAM and HAR). HAR was performed with a cluster of charges and dipoles modeling crystal environment (DiSCaMB) and without a cluster (NoSpherA2). DFT calculations for HAR were performed with various functionals in the non-relativistic version (B3LYP, PBE and M06-2X) and with relativistic correction (B3LYP-DKH2, PBE-DKH2 and M06-2X-DKH2). Basis sets used: (a) non-relativistic refinements: jorge-TZP, (b) relativistic refinements: jorge-TZP-DKH.

				DiSCaMB-HAR						NoSpherA2-HAR					
**bond**		**neutron**	**IAM**	**B3LYP**	**PBE**	**M06-2X**	**B3LYP-DKH2**	**PBE-DKH2**	**M06-2X-DKH2**	**B3LYP**	**PBE**	**M06-2X**	**B3LYP-DKH2**	**PBE-DKH2**	**M06-2X-DKH2**
**Sb**	**H1A**	1.73(2)	1.74(3)	1.84(2)	1.84(2)	1.84(2)	1.84(2)	1.84(2)	1.83(2)	1.77(3)	1.77(3)	1.77(3)	1.77(3)	1.77(3)	1.77(3)
**C3**	**H3**	1.11(3)	0.88(3)	1.09(3)	1.09(3)	1.08(3)	1.09(3)	1.09(3)	1.08(3)	0.95(3)	0.95(3)	0.95(3)	0.95(3)	0.95(3)	0.95(3)
**C4**	**H4**	1.10(3)	0.96(3)	1.15(3)	1.15(3)	1.15(3)	1.14(3)	1.15(3)	1.14(3)	1.05(3)	1.05(3)	1.05(3)	1.05(3)	1.05(3)	1.05(3)
**C5**	**H5**	1.03(3)	0.92(3)	1.05(2)	1.05(2)	1.04(2)	1.04(2)	1.05(2)	1.04(2)	0.99(3)	0.99(3)	0.99(3)	0.99(3)	0.99(3)	0.99(3)
**C7**	**H7A**	1.10(3)	0.92(3)	1.06(3)	1.07(3)	1.06(3)	1.06(3)	1.06(3)	1.06(3)	0.97(4)	0.97(4)	0.97(4)	0.97(4)	0.97(4)	0.97(4)
**C7**	**H7B**	1.08(4)	0.90(4)	1.04(4)	1.05(4)	1.04(4)	1.04(4)	1.05(4)	1.04(4)	1.00(4)	1.00(4)	1.00(4)	1.00(4)	1.00(4)	1.00(4)
**C7**	**H7C**	1.04(5)	1.01(4)	1.13(4)	1.13(4)	1.13(4)	1.12(4)	1.13(4)	1.12(4)	1.08(4)	1.08(4)	1.08(4)	1.08(4)	1.08(4)	1.08(4)
**C8**	**H8A**	1.08(NA)	0.85(5)	1.00(6)	1.00(5)	0.99(5)	0.99(6)	1.00(5)	0.99(5)	0.96(6)	0.96(6)	0.95(6)	0.96(6)	0.96(6)	0.95(6)
**C8**	**H8B**	1.08(NA)	0.90(4)	1.09(4)	1.10(4)	1.09(4)	1.09(4)	1.09(4)	1.09(4)	0.97(4)	0.97(4)	0.97(4)	0.97(4)	0.97(4)	0.97(4)
**C8**	**H8C**	1.08(NA)	0.96(4)	1.07(4)	1.07(4)	1.07(4)	1.07(4)	1.07(4)	1.07(4)	1.03(4)	1.03(5)	1.03(4)	1.03(4)	1.03(5)	1.03(4)
**C9**	**H9A**	1.06(3)	0.95(4)	1.08(3)	1.08(3)	1.08(3)	1.08(3)	1.08(3)	1.08(3)	1.02(4)	1.02(4)	1.01(4)	1.02(4)	1.02(4)	1.01(4)
**C9**	**H9B**	1.09(6)	0.96(4)	1.11(4)	1.12(4)	1.11(4)	1.11(4)	1.11(4)	1.11(4)	0.99(5)	0.99(5)	0.98(5)	0.99(5)	0.99(5)	0.98(5)
**C9**	**H9C**	1.09(4)	0.95(4)	1.10(4)	1.11(4)	1.10(4)	1.10(4)	1.10(4)	1.10(4)	0.97(4)	0.97(4)	0.97(4)	0.97(4)	0.97(4)	0.97(4)
**C10**	**H10A**	1.07(3)	0.90(4)	1.06(4)	1.06(4)	1.06(4)	1.06(4)	1.06(4)	1.06(4)	0.96(4)	0.96(4)	0.96(4)	0.96(4)	0.96(4)	0.96(4)
**C10**	**H10B**	1.08(4)	1.04(5)	1.19(4)	1.20(4)	1.19(4)	1.19(4)	1.19(4)	1.19(4)	1.07(5)	1.07(5)	1.07(5)	1.07(5)	1.07(5)	1.07(5)
**C10**	**H10C**	0.97(4)	0.89(4)	1.04(4)	1.04(4)	1.03(4)	1.04(4)	1.03(3)	1.03(3)	0.93(4)	0.93(4)	0.92(4)	0.93(4)	0.93(4)	0.92(4)
**C11**	**H11A**	1.07(4)	0.94(3)	1.09(3)	1.09(3)	1.09(3)	1.08(3)	1.09(3)	1.08(3)	0.99(3)	0.99(3)	0.99(3)	0.99(3)	0.99(3)	0.99(3)
**C11**	**H11B**	1.05(4)	0.95(3)	1.07(3)	1.07(3)	1.07(3)	1.07(3)	1.07(3)	1.07(3)	1.00(4)	1.00(4)	1.00(4)	1.00(4)	1.00(4)	1.00(4)
**C11**	**H11C**	1.11(3)	0.95(3)	1.08(3)	1.08(3)	1.08(3)	1.08(3)	1.08(3)	1.08(3)	1.00(4)	1.00(4)	1.00(4)	1.00(4)	1.00(4)	1.00(4)
**C12**	**H12A**	1.05(4)	0.91(4)	1.10(3)	1.10(3)	1.10(3)	1.10(3)	1.10(3)	1.10(3)	0.98(4)	0.98(4)	0.97(4)	0.98(4)	0.98(4)	0.97(4)
**C12**	**H12B**	1.01(6)	0.93(4)	1.11(4)	1.11(4)	1.10(4)	1.11(4)	1.11(4)	1.11(4)	1.03(4)	1.03(4)	1.03(4)	1.03(4)	1.03(4)	1.03(4)
**C12**	**H12C**	1.02(5)	0.89(4)	1.04(4)	1.04(4)	1.04(4)	1.04(4)	1.04(4)	1.04(4)	0.93(5)	0.93(5)	0.93(5)	0.93(5)	0.93(5)	0.93(5)
**C15**	**H15**	1.06(4)	0.92(3)	1.08(3)	1.09(3)	1.08(3)	1.08(3)	1.08(3)	1.08(3)	1.00(3)	1.00(3)	1.00(3)	1.00(3)	1.00(3)	1.00(3)
**C16**	**H16**	1.07(4)	0.93(3)	1.10(3)	1.11(3)	1.10(3)	1.10(3)	1.10(3)	1.10(3)	0.99(3)	0.99(3)	0.99(3)	0.99(3)	0.99(3)	0.99(3)
**C17**	**H17**	1.07(3)	0.94(3)	1.08(3)	1.08(3)	1.08(3)	1.08(3)	1.08(3)	1.08(3)	0.99(3)	0.99(3)	0.99(3)	0.99(3)	0.99(3)	0.99(3)
**C19**	**H19A**	1.08(4)	0.96(3)	1.07(4)	1.07(4)	1.07(3)	1.07(4)	1.07(3)	1.07(3)	1.02(4)	1.02(4)	1.02(4)	1.02(4)	1.02(4)	1.02(4)

**C19**	**H19B**	1.10(4)	0.95(3)	1.10(3)	1.10(3)	1.10(3)	1.10(3)	1.10(3)	1.10(3)	1.00(4)	1.00(4)	1.00(4)	1.00(4)	1.00(4)	1.00(4)
**C19**	**H19C**	1.01(5)	0.92(4)	1.09(4)	1.09(4)	1.09(4)	1.09(4)	1.09(4)	1.08(4)	0.99(4)	0.99(4)	0.99(4)	0.99(4)	0.99(4)	0.99(4)
**C20**	**H20A**	1.15(4)	0.96(4)	1.09(4)	1.10(4)	1.09(4)	1.09(4)	1.09(4)	1.09(4)	1.01(4)	1.01(4)	1.01(4)	1.01(4)	1.01(4)	1.01(4)
**C20**	**H20B**	1.02(5)	0.92(4)	1.09(4)	1.10(4)	1.09(4)	1.09(4)	1.09(4)	1.08(4)	0.99(4)	0.99(4)	0.99(4)	0.99(4)	0.99(4)	0.99(4)
**C20**	**H20C**	1.11(4)	0.91(4)	1.05(4)	1.05(4)	1.05(4)	1.05(4)	1.05(4)	1.05(4)	0.99(5)	0.99(5)	0.98(5)	0.99(5)	0.99(5)	0.98(5)

Table S27 X-H bond lengths (units: Å) obtained for XAXMEP with various experimental methods (neutron and X-ray) and refinement methods (IAM and HAR). HAR was performed with a cluster of charges and dipoles modeling crystal environment (DiSCaMB) and without a cluster (NoSpherA2). DFT calculations for HAR were performed with various functionals in the non-relativistic version (B3LYP, PBE and M06-2X) and with relativistic correction (B3LYP-DKH2, PBE-DKH2 and M06-2X-DKH2). Basis sets used: (a) non-relativistic refinements: jorge-DZP, (b) relativistic refinements: jorge-DZP-DKH.

				DiSCaMB-HAR						NoSpherA2-HAR					
**bond**		**neutron**	**IAM**	**B3LYP**	**PBE**	**M06-2X**	**B3LYP-DKH2**	**PBE-DKH2**	**M06-2X-DKH2**	**B3LYP**	**PBE**	**M06-2X**	**B3LYP-DKH2**	**PBE-DKH2**	**M06-2X-DKH2**
**Os**	**H1**	1.606(17)	1.63(4)	1.68(3)	1.68(3)	1.68(3)	1.68(3)	1.68(3)	1.69(3)	1.70(4)	1.70(5)	1.71(5)	1.70(4)	1.70(4)	1.71(4)
**Os**	**H2**	1.632(15)	1.61(5)	1.66(5)	1.66(4)	1.66(4)	1.66(5)	1.67(5)	1.67(5)	1.67(5)	1.67(5)	1.67(5)	1.69(5)	1.68(5)	1.69(5)
**Os**	**H3**	1.599(21)	1.60(6)	1.68(5)	1.68(5)	1.68(5)	1.68(5)	1.68(5)	1.68(5)	1.69(6)	1.69(6)	1.69(6)	1.68(6)	1.68(6)	1.69(6)
**Os**	**H4**	1.626(19)	1.60(7)	1.66(5)	1.66(5)	1.66(5)	1.65(5)	1.65(5)	1.65(5)	1.72(7)	1.72(7)	1.72(7)	1.70(7)	1.70(7)	1.70(7)
**C6**	**H6A**	1.092(25)	0.980(6)	1.10(7)	1.10(7)	1.10(7)	1.08(7)	1.08(7)	1.08(7)	1.10(9)	1.10(9)	1.10(8)	1.09(8)	1.09(8)	1.09(8)
**C6**	**H6B**	1.092(26)	0.979(6)	1.05(6)	1.05(6)	1.05(6)	1.04(6)	1.04(6)	1.04(6)	1.09(8)	1.09(8)	1.08(8)	1.09(8)	1.09(8)	1.09(8)
**C6**	**H6C**	1.093(23)	0.980(6)	1.14(6)	1.14(6)	1.14(6)	1.14(6)	1.14(6)	1.14(6)	1.14(9)	1.14(9)	1.14(9)	1.13(9)	1.14(8)	1.13(8)
**C8**	**H8A**	1.092(25)	0.981(8)	1.08(9)	1.09(9)	1.08(9)	1.09(9)	1.09(9)	1.09(9)	1.13(11)	1.13(11)	1.13(11)	1.14(11)	1.14(10)	1.14(11)
**C8**	**H8B**	1.091(28)	0.980(6)	1.16(10)	1.17(10)	1.16(10)	1.15(10)	1.15(10)	1.15(10)	1.11(15)	1.11(14)	1.10(15)	1.09(14)	1.09(14)	1.08(14)
**C8**	**H8C**	1.096(23)	0.981(7)	1.12(8)	1.12(8)	1.12(8)	1.11(8)	1.11(8)	1.11(8)	1.13(10)	1.13(10)	1.13(10)	1.13(10)	1.13(10)	1.13(10)
**C7**	**H7A**	1.093(25)	0.979(6)	1.00(8)	1.00(8)	1.00(8)	1.00(8)	1.00(7)	0.99(7)	1.00(10)	1.01(10)	1.00(9)	1.01(9)	1.01(9)	1.01(9)
**C7**	**H7B**	1.084(27)	0.981(6)	1.02(8)	1.03(8)	1.02(8)	1.01(8)	1.02(8)	1.01(8)	1.07(12)	1.07(12)	1.07(12)	1.06(12)	1.06(11)	1.06(12)
**C7**	**H7C**	1.092(27)	0.979(7)	1.12(9)	1.12(8)	1.11(8)	1.10(8)	1.10(8)	1.09(8)	1.10(11)	1.10(11)	1.09(11)	1.08(11)	1.08(10)	1.08(10)
**C9**	**H9A**	1.094(26)	0.980(6)	1.12(6)	1.12(6)	1.11(6)	1.10(6)	1.10(6)	1.10(6)	1.11(8)	1.12(8)	1.11(8)	1.10(8)	1.10(8)	1.10(8)
**C9**	**H9B**	1.092(25)	0.980(7)	1.04(7)	1.03(7)	1.03(7)	1.04(7)	1.04(6)	1.04(6)	1.08(8)	1.08(8)	1.08(8)	1.07(8)	1.07(8)	1.07(8)
**C9**	**H9C**	1.093(25)	0.979(7)	1.18(7)	1.18(7)	1.18(7)	1.17(7)	1.17(7)	1.17(7)	1.15(10)	1.15(10)	1.15(10)	1.15(9)	1.15(9)	1.15(9)
**C10**	**H10A**	1.096(27)	0.981(6)	1.06(8)	1.06(8)	1.05(8)	1.05(8)	1.05(8)	1.04(8)	1.10(10)	1.11(10)	1.10(10)	1.10(9)	1.10(9)	1.10(9)
**C10**	**H10B**	1.089(24)	0.980(6)	1.11(8)	1.11(8)	1.11(8)	1.10(8)	1.10(8)	1.09(8)	1.13(11)	1.13(11)	1.12(11)	1.12(10)	1.12(10)	1.12(10)
**C10**	**H10C**	1.093(29)	0.980(7)	1.18(7)	1.19(7)	1.19(7)	1.18(7)	1.18(7)	1.18(7)	1.13(8)	1.13(8)	1.13(8)	1.13(8)	1.13(7)	1.13(8)
**C11**	**H11**	1.093(26)	0.999(4)	1.14(5)	1.14(5)	1.13(5)	1.14(5)	1.14(5)	1.14(5)	1.12(6)	1.12(6)	1.12(6)	1.12(6)	1.12(6)	1.12(6)
**C12**	**H12A**	1.096(20)	0.989(5)	1.16(6)	1.16(6)	1.16(6)	1.16(6)	1.16(5)	1.16(5)	1.16(7)	1.16(7)	1.16(6)	1.16(6)	1.16(6)	1.16(6)
**C12**	**H12B**	1.092(26)	0.990(5)	1.16(5)	1.16(5)	1.15(5)	1.13(5)	1.13(4)	1.13(4)	1.10(6)	1.10(6)	1.10(6)	1.09(6)	1.09(6)	1.09(6)
**C13**	**H13A**	1.096(26)	0.989(6)	1.12(5)	1.12(5)	1.12(5)	1.12(5)	1.12(5)	1.12(5)	1.08(7)	1.08(7)	1.08(7)	1.08(7)	1.08(6)	1.08(6)
**C13**	**H13B**	1.094(21)	0.990(5)	1.10(7)	1.11(7)	1.10(7)	1.10(7)	1.10(7)	1.10(7)	1.14(9)	1.14(9)	1.14(9)	1.14(9)	1.14(9)	1.14(9)
**C14**	**H14A**	1.094(26)	0.990(6)	1.13(6)	1.13(6)	1.12(6)	1.12(6)	1.12(6)	1.12(6)	1.13(8)	1.13(8)	1.12(8)	1.12(8)	1.12(8)	1.12(8)
**C14**	**H14B**	1.093(20)	0.990(5)	1.09(7)	1.09(7)	1.09(7)	1.09(7)	1.09(7)	1.09(7)	1.07(8)	1.07(8)	1.07(8)	1.05(8)	1.05(8)	1.05(8)

**C15**	**H15A**	1.089(21)	0.990(6)	1.11(5)	1.11(5)	1.11(5)	1.11(5)	1.11(5)	1.11(5)	1.12(7)	1.12(7)	1.12(7)	1.11(7)	1.11(7)	1.11(7)
**C15**	**H15B**	1.097(26)	0.990(6)	1.16(5)	1.16(5)	1.15(5)	1.16(5)	1.16(5)	1.16(5)	1.17(7)	1.17(7)	1.17(7)	1.17(7)	1.17(6)	1.17(7)
**C16**	**H16A**	1.095(20)	0.990(5)	1.10(6)	1.10(6)	1.10(6)	1.08(6)	1.09(6)	1.09(6)	1.11(8)	1.11(8)	1.11(8)	1.11(8)	1.11(8)	1.11(8)
**C16**	**H16B**	1.098(25)	0.990(5)	1.11(5)	1.11(5)	1.11(5)	1.11(5)	1.10(5)	1.10(5)	1.10(6)	1.10(6)	1.10(6)	1.10(6)	1.10(6)	1.10(6)
**C21**	**H21**	1.097(21)	1.000(4)	1.10(5)	1.09(5)	1.09(5)	1.09(5)	1.08(5)	1.08(5)	1.13(6)	1.13(6)	1.13(6)	1.13(6)	1.13(6)	1.13(6)
**C22**	**H22A**	1.095(27)	0.990(5)	1.18(5)	1.18(5)	1.17(5)	1.17(5)	1.17(5)	1.16(5)	1.13(7)	1.13(7)	1.13(7)	1.13(7)	1.13(7)	1.13(7)
**C22**	**H22B**	1.100(20)	0.990(5)	1.12(6)	1.12(5)	1.11(5)	1.11(5)	1.11(5)	1.11(5)	1.10(7)	1.10(7)	1.10(7)	1.09(7)	1.09(7)	1.09(7)
**C23**	**H23A**	1.102(21)	0.990(5)	1.18(5)	1.18(5)	1.17(5)	1.17(5)	1.17(5)	1.17(5)	1.15(7)	1.15(7)	1.15(6)	1.15(6)	1.15(6)	1.14(6)
**C23**	**H23B**	1.099(21)	0.989(5)	1.13(5)	1.14(5)	1.13(5)	1.12(5)	1.13(5)	1.13(5)	1.15(7)	1.15(7)	1.15(7)	1.15(7)	1.15(7)	1.15(7)
**C24**	**H24A**	1.094(21)	0.991(5)	1.12(5)	1.12(5)	1.12(5)	1.12(5)	1.12(5)	1.12(5)	1.12(6)	1.12(6)	1.12(6)	1.11(6)	1.11(6)	1.11(6)
**C24**	**H24B**	1.100(20)	0.989(5)	1.18(6)	1.19(6)	1.18(6)	1.18(6)	1.18(6)	1.18(6)	1.21(7)	1.21(7)	1.21(7)	1.21(7)	1.21(7)	1.21(7)
**C25**	**H25A**	1.091(27)	0.989(5)	1.10(5)	1.10(5)	1.10(5)	1.10(5)	1.10(5)	1.10(5)	1.11(8)	1.11(8)	1.12(7)	1.11(7)	1.11(7)	1.11(7)
**C25**	**H25B**	1.098(21)	0.989(5)	1.17(6)	1.17(6)	1.16(6)	1.15(6)	1.14(5)	1.14(5)	1.12(7)	1.12(7)	1.11(7)	1.12(7)	1.12(7)	1.12(7)
**C26**	**H26A**	1.093(21)	0.989(5)	1.03(4)	1.03(4)	1.03(4)	1.03(4)	1.04(4)	1.03(4)	1.04(6)	1.04(6)	1.04(6)	1.04(6)	1.04(6)	1.04(6)
**C26**	**H26B**	1.097(20)	0.989(5)	1.16(5)	1.16(5)	1.16(5)	1.16(5)	1.16(5)	1.16(5)	1.17(6)	1.17(6)	1.17(6)	1.17(6)	1.17(6)	1.17(6)
**C31**	**H31**	1.099(22)	1.002(4)	1.17(4)	1.17(4)	1.17(4)	1.17(4)	1.17(4)	1.17(4)	1.19(4)	1.19(5)	1.19(4)	1.19(4)	1.18(4)	1.19(4)
**C32**	**H32A**	1.092(19)	0.989(5)	1.12(6)	1.12(6)	1.12(6)	1.11(6)	1.12(6)	1.11(6)	1.13(8)	1.13(7)	1.13(7)	1.12(7)	1.12(7)	1.12(7)
**C32**	**H32B**	1.102(22)	0.990(5)	1.07(5)	1.07(5)	1.07(5)	1.07(5)	1.08(5)	1.07(5)	1.10(6)	1.10(6)	1.10(6)	1.10(6)	1.10(6)	1.10(6)
**C33**	**H33A**	1.092(21)	0.991(5)	1.17(5)	1.17(5)	1.17(5)	1.15(5)	1.16(5)	1.16(5)	1.19(6)	1.19(6)	1.19(6)	1.18(6)	1.18(6)	1.18(6)
**C33**	**H33B**	1.091(24)	0.988(5)	1.14(6)	1.14(6)	1.14(6)	1.15(6)	1.15(6)	1.15(6)	1.13(7)	1.13(7)	1.13(7)	1.12(7)	1.12(7)	1.12(7)
**C34**	**H34A**	1.090(21)	0.991(5)	1.10(6)	1.10(6)	1.10(6)	1.10(5)	1.11(5)	1.10(5)	1.11(7)	1.11(7)	1.11(7)	1.11(7)	1.11(7)	1.11(7)
**C34**	**H34B**	1.091(21)	0.989(5)	1.06(5)	1.06(5)	1.06(5)	1.06(5)	1.06(4)	1.06(4)	1.04(6)	1.04(6)	1.04(6)	1.04(6)	1.04(6)	1.04(6)
**C35**	**H35A**	1.097(19)	0.990(5)	1.21(5)	1.22(5)	1.21(5)	1.21(5)	1.21(5)	1.21(5)	1.20(7)	1.20(7)	1.20(7)	1.20(7)	1.20(6)	1.20(6)
**C35**	**H35B**	1.102(21)	0.990(5)	1.17(6)	1.18(6)	1.18(6)	1.16(6)	1.17(6)	1.17(6)	1.19(8)	1.19(8)	1.19(8)	1.19(7)	1.19(7)	1.19(7)
**C36**	**H36A**	1.100(21)	0.991(5)	1.20(5)	1.21(5)	1.21(5)	1.19(5)	1.20(5)	1.20(5)	1.15(6)	1.15(6)	1.15(6)	1.15(6)	1.15(6)	1.15(6)
**C36**	**H36B**	1.098(24)	0.990(4)	1.06(5)	1.06(5)	1.05(5)	1.05(5)	1.05(5)	1.05(5)	1.08(6)	1.08(6)	1.08(6)	1.07(6)	1.07(6)	1.07(6)

Table S28 X-H bond lengths (units: Å) obtained for XAXMEP with various experimental methods (neutron and X-ray) and refinement methods (IAM and HAR). HAR was performed with a cluster of charges and dipoles modeling crystal environment (DiSCaMB) and without a cluster (NoSpherA2). DFT calculations for HAR were performed with various functionals in the non-relativistic version (B3LYP, PBE and M06-2X) and with relativistic correction (B3LYP-DKH2, PBE-DKH2 and M06-2X-DKH2). Basis sets used: (a) non-relativistic refinements: jorge-TZP, (b) relativistic refinements: jorge-TZP-DKH.

				DiSCaMB-HAR						NoSpherA2-HAR					
**bond**		**neutron**	**IAM**	**B3LYP**	**PBE**	**M06-2X**	**B3LYP-DKH2**	**PBE-DKH2**	**M06-2X-DKH2**	**B3LYP**	**PBE**	**M06-2X**	**B3LYP-DKH2**	**PBE-DKH2**	**M06-2X-DKH2**
**Os**	**H1**	1.606(17)	1.63(4)	1.67(3)	1.68(4)	1.68(4)	1.68(3)	1.68(3)	1.68(4)	1.69(5)	1.69(5)	1.70(5)	1.70(4)	1.70(4)	1.71(5)
**Os**	**H2**	1.632(15)	1.61(5)	1.66(5)	1.66(4)	1.66(4)	1.66(5)	1.66(4)	1.66(4)	1.65(5)	1.65(5)	1.66(5)	1.68(5)	1.68(5)	1.68(5)
**Os**	**H3**	1.599(21)	1.60(6)	1.68(5)	1.68(5)	1.68(5)	1.68(5)	1.68(5)	1.69(5)	1.68(6)	1.68(6)	1.68(6)	1.68(6)	1.68(6)	1.69(6)
**Os**	**H4**	1.626(19)	1.60(7)	1.65(5)	1.65(5)	1.66(5)	1.64(5)	1.65(5)	1.65(5)	1.70(7)	1.70(7)	1.71(7)	1.69(7)	1.69(7)	1.70(7)
**C6**	**H6A**	1.092(25)	0.980(6)	1.09(7)	1.09(7)	1.09(7)	1.07(7)	1.08(7)	1.07(7)	1.09(9)	1.09(9)	1.09(9)	1.08(8)	1.08(8)	1.08(8)
**C6**	**H6B**	1.092(26)	0.979(6)	1.05(6)	1.04(6)	1.04(6)	1.04(6)	1.04(6)	1.03(6)	1.08(8)	1.08(8)	1.08(8)	1.09(8)	1.09(8)	1.08(8)
**C6**	**H6C**	1.093(23)	0.980(6)	1.13(6)	1.14(6)	1.14(6)	1.14(6)	1.14(6)	1.14(6)	1.13(9)	1.13(9)	1.13(9)	1.13(9)	1.13(8)	1.13(8)
**C8**	**H8A**	1.092(25)	0.981(8)	1.08(9)	1.08(9)	1.08(9)	1.09(9)	1.09(9)	1.08(9)	1.13(11)	1.13(11)	1.12(11)	1.14(11)	1.14(11)	1.13(11)
**C8**	**H8B**	1.091(28)	0.980(6)	1.14(10)	1.14(10)	1.13(10)	1.12(10)	1.13(10)	1.12(10)	1.08(14)	1.09(14)	1.08(14)	1.07(14)	1.07(14)	1.07(14)
**C8**	**H8C**	1.096(23)	0.981(7)	1.11(8)	1.12(8)	1.11(8)	1.11(8)	1.11(8)	1.11(8)	1.12(10)	1.12(10)	1.12(10)	1.13(10)	1.13(10)	1.13(10)
**C7**	**H7A**	1.093(25)	0.979(6)	0.99(8)	0.99(8)	0.99(8)	0.99(8)	0.99(7)	0.98(7)	1.00(10)	1.00(10)	0.99(10)	1.01(10)	1.01(9)	1.00(9)
**C7**	**H7B**	1.084(27)	0.981(6)	1.02(8)	1.03(8)	1.03(8)	1.02(8)	1.02(8)	1.02(8)	1.06(12)	1.07(12)	1.06(12)	1.05(12)	1.06(12)	1.05(12)
**C7**	**H7C**	1.092(27)	0.979(7)	1.11(9)	1.10(8)	1.09(8)	1.09(8)	1.08(8)	1.08(8)	1.08(11)	1.08(11)	1.08(11)	1.08(11)	1.08(11)	1.07(10)
**C9**	**H9A**	1.094(26)	0.980(6)	1.11(6)	1.11(6)	1.11(6)	1.09(6)	1.10(6)	1.09(6)	1.11(8)	1.11(8)	1.11(8)	1.10(8)	1.10(8)	1.10(8)
**C9**	**H9B**	1.092(25)	0.980(7)	1.03(7)	1.03(7)	1.03(7)	1.04(6)	1.04(6)	1.03(6)	1.07(8)	1.07(8)	1.07(8)	1.07(8)	1.07(8)	1.06(8)
**C9**	**H9C**	1.093(25)	0.979(7)	1.17(7)	1.17(7)	1.17(7)	1.16(7)	1.16(7)	1.16(7)	1.14(10)	1.14(10)	1.13(10)	1.14(9)	1.14(9)	1.14(9)
**C10**	**H10A**	1.096(27)	0.981(6)	1.04(8)	1.05(8)	1.04(8)	1.03(8)	1.04(8)	1.03(8)	1.09(10)	1.09(10)	1.09(10)	1.09(9)	1.10(9)	1.09(9)
**C10**	**H10B**	1.089(24)	0.980(6)	1.11(8)	1.11(8)	1.10(8)	1.09(7)	1.10(8)	1.09(8)	1.11(11)	1.11(11)	1.11(11)	1.11(10)	1.11(10)	1.11(10)
**C10**	**H10C**	1.093(29)	0.980(7)	1.18(7)	1.18(7)	1.18(7)	1.17(7)	1.18(7)	1.18(7)	1.12(8)	1.12(8)	1.12(8)	1.13(8)	1.13(8)	1.13(8)
**C11**	**H11**	1.093(26)	0.999(4)	1.13(5)	1.13(5)	1.13(5)	1.14(5)	1.13(5)	1.13(5)	1.11(6)	1.11(6)	1.11(6)	1.12(6)	1.11(6)	1.11(6)
**C12**	**H12A**	1.096(20)	0.989(5)	1.16(5)	1.16(5)	1.15(5)	1.16(5)	1.16(5)	1.15(5)	1.16(7)	1.16(6)	1.15(6)	1.16(6)	1.16(6)	1.16(6)
**C12**	**H12B**	1.092(26)	0.990(5)	1.16(5)	1.15(5)	1.15(5)	1.13(4)	1.13(4)	1.13(4)	1.10(6)	1.10(6)	1.10(6)	1.09(6)	1.09(6)	1.09(6)
**C13**	**H13A**	1.096(26)	0.989(6)	1.11(5)	1.12(5)	1.11(5)	1.12(5)	1.12(5)	1.11(5)	1.07(7)	1.07(7)	1.07(7)	1.07(7)	1.07(7)	1.07(7)
**C13**	**H13B**	1.094(21)	0.990(5)	1.10(7)	1.10(7)	1.10(7)	1.09(7)	1.10(7)	1.09(7)	1.14(9)	1.14(9)	1.14(9)	1.14(9)	1.14(9)	1.14(9)
**C14**	**H14A**	1.094(26)	0.990(6)	1.12(6)	1.12(6)	1.12(6)	1.11(6)	1.12(6)	1.11(6)	1.12(9)	1.12(8)	1.12(8)	1.12(8)	1.12(8)	1.12(8)
**C14**	**H14B**	1.093(20)	0.990(5)	1.08(7)	1.08(7)	1.08(7)	1.08(7)	1.08(7)	1.08(7)	1.06(8)	1.06(8)	1.06(8)	1.05(8)	1.05(8)	1.05(8)

**C15**	**H15A**	1.089(21)	0.990(6)	1.11(5)	1.11(5)	1.11(5)	1.11(5)	1.11(5)	1.10(5)	1.11(7)	1.11(7)	1.11(7)	1.11(7)	1.11(7)	1.11(7)
**C15**	**H15B**	1.097(26)	0.990(6)	1.15(5)	1.15(5)	1.15(5)	1.16(5)	1.16(5)	1.16(5)	1.17(7)	1.17(7)	1.17(7)	1.17(7)	1.17(6)	1.16(7)
**C16**	**H16A**	1.095(20)	0.990(5)	1.10(6)	1.10(6)	1.10(6)	1.08(6)	1.09(6)	1.09(6)	1.11(8)	1.11(8)	1.11(8)	1.11(8)	1.11(8)	1.11(8)
**C16**	**H16B**	1.098(25)	0.990(5)	1.11(5)	1.11(5)	1.10(5)	1.10(5)	1.10(5)	1.10(5)	1.10(6)	1.10(6)	1.10(6)	1.10(6)	1.10(6)	1.10(6)
**C21**	**H21**	1.097(21)	1.000(4)	1.09(5)	1.09(5)	1.09(5)	1.08(5)	1.08(5)	1.08(5)	1.13(6)	1.13(6)	1.12(6)	1.13(6)	1.13(6)	1.13(6)
**C22**	**H22A**	1.095(27)	0.990(5)	1.17(5)	1.17(5)	1.16(5)	1.16(5)	1.16(5)	1.16(5)	1.13(7)	1.13(7)	1.12(7)	1.13(7)	1.13(7)	1.13(7)
**C22**	**H22B**	1.100(20)	0.990(5)	1.11(5)	1.11(5)	1.10(5)	1.10(5)	1.10(5)	1.10(5)	1.09(7)	1.09(7)	1.09(7)	1.09(7)	1.09(7)	1.08(7)
**C23**	**H23A**	1.102(21)	0.990(5)	1.18(5)	1.18(5)	1.17(5)	1.17(5)	1.17(5)	1.17(5)	1.15(7)	1.15(7)	1.14(7)	1.15(6)	1.14(6)	1.14(6)
**C23**	**H23B**	1.099(21)	0.989(5)	1.12(5)	1.13(5)	1.13(5)	1.11(5)	1.12(5)	1.12(5)	1.15(7)	1.15(7)	1.15(7)	1.15(7)	1.15(7)	1.15(7)
**C24**	**H24A**	1.094(21)	0.991(5)	1.11(5)	1.12(5)	1.11(5)	1.11(5)	1.12(5)	1.11(5)	1.11(7)	1.11(6)	1.11(6)	1.10(6)	1.11(6)	1.10(6)
**C24**	**H24B**	1.100(20)	0.989(5)	1.18(6)	1.18(6)	1.18(6)	1.18(6)	1.18(6)	1.18(6)	1.21(7)	1.21(7)	1.21(7)	1.20(7)	1.21(7)	1.21(7)
**C25**	**H25A**	1.091(27)	0.989(5)	1.09(5)	1.09(5)	1.09(5)	1.10(5)	1.10(5)	1.09(5)	1.11(8)	1.11(8)	1.11(8)	1.11(7)	1.11(7)	1.11(7)
**C25**	**H25B**	1.098(21)	0.989(5)	1.16(6)	1.16(6)	1.15(6)	1.14(6)	1.14(5)	1.13(5)	1.11(7)	1.11(7)	1.11(7)	1.11(7)	1.11(7)	1.11(7)
**C26**	**H26A**	1.093(21)	0.989(5)	1.03(5)	1.03(5)	1.03(5)	1.03(4)	1.03(4)	1.03(5)	1.04(7)	1.04(7)	1.03(7)	1.04(6)	1.04(6)	1.03(6)
**C26**	**H26B**	1.097(20)	0.989(5)	1.16(5)	1.16(5)	1.16(5)	1.16(5)	1.16(5)	1.16(5)	1.17(6)	1.17(6)	1.16(6)	1.16(6)	1.16(6)	1.16(6)
**C31**	**H31**	1.099(22)	1.002(4)	1.17(3)	1.17(3)	1.17(3)	1.16(3)	1.17(3)	1.17(3)	1.18(4)	1.18(4)	1.18(4)	1.18(4)	1.18(4)	1.18(4)
**C32**	**H32A**	1.092(19)	0.989(5)	1.12(6)	1.12(6)	1.12(6)	1.11(6)	1.12(6)	1.11(6)	1.12(8)	1.13(7)	1.12(7)	1.12(7)	1.12(7)	1.12(7)
**C32**	**H32B**	1.102(22)	0.990(5)	1.07(5)	1.07(5)	1.07(5)	1.07(5)	1.07(5)	1.07(5)	1.10(6)	1.10(6)	1.10(6)	1.10(6)	1.10(6)	1.10(6)
**C33**	**H33A**	1.092(21)	0.991(5)	1.16(5)	1.17(5)	1.16(5)	1.15(5)	1.15(5)	1.15(5)	1.18(6)	1.19(6)	1.19(6)	1.18(6)	1.18(6)	1.18(6)
**C33**	**H33B**	1.091(24)	0.988(5)	1.13(6)	1.13(6)	1.13(6)	1.14(6)	1.14(5)	1.14(5)	1.13(7)	1.13(7)	1.12(7)	1.12(7)	1.12(7)	1.12(7)
**C34**	**H34A**	1.090(21)	0.991(5)	1.10(6)	1.10(6)	1.10(6)	1.10(5)	1.10(5)	1.10(5)	1.11(7)	1.11(7)	1.11(7)	1.11(7)	1.11(7)	1.11(7)
**C34**	**H34B**	1.091(21)	0.989(5)	1.05(5)	1.05(5)	1.05(5)	1.05(5)	1.05(4)	1.05(5)	1.04(6)	1.04(6)	1.04(6)	1.04(6)	1.04(6)	1.04(6)
**C35**	**H35A**	1.097(19)	0.990(5)	1.21(5)	1.21(5)	1.21(5)	1.21(5)	1.21(5)	1.21(5)	1.20(7)	1.19(7)	1.19(7)	1.20(7)	1.20(6)	1.20(6)
**C35**	**H35B**	1.102(21)	0.990(5)	1.17(6)	1.18(6)	1.18(6)	1.17(6)	1.17(6)	1.17(6)	1.19(8)	1.19(8)	1.19(8)	1.19(7)	1.19(7)	1.19(7)
**C36**	**H36A**	1.100(21)	0.991(5)	1.20(5)	1.20(5)	1.20(5)	1.19(5)	1.20(5)	1.20(5)	1.15(6)	1.15(6)	1.15(6)	1.15(6)	1.15(6)	1.15(6)
**C36**	**H36B**	1.098(24)	0.990(4)	1.06(5)	1.06(5)	1.06(5)	1.05(5)	1.05(5)	1.05(5)	1.08(7)	1.08(7)	1.08(7)	1.07(6)	1.07(6)	1.07(6)

S1. Ranking of structures by data quality (neutron and X-ray):

S1.1. Ranking of structures by completeness (from the worst to the best):

Neutron: 1-NEBNEO, 1-UJABOX, 1-XAXMEP, 1-ZEYVAA, 2-GOJNIF, 3-SITKUB, 4-QOSZON, 4- TIWXOP, 5-MIGKIY, 6-MIGKIY, 7-NOBBOX

X-ray: 1-ZEYVAA, 2-MIGKIY, 3-UJABOX, 4-XAXMEP, 5-QOSZON, 6-NEBNEO, 7-TIWXOP, 8-SITKUB, 9-GOJNIF, 10-NOBBOX

S1.2. Ranking of structures by R_{int} (from the worst to the best):

Neutron: 1-GOJNIF, 1-TIWXOP, 1-NEBNEO, 1-XAXMEP, 2-MIGKIY, 3-UJABOX, 4-ZEYVAA, 5-SITKUB, 6-NOBBOX, 7-QOSZON

X-ray: 1-GOJNIF, 1-TIWXOP, 1-NEBNEO, 2-ZEYVAA, 3-UJABOX, 4-MIGKIY, 5-QOSZON, 6-XAXMEP, 7-SITKUB, 8-NOBBOX

S1.3. Ranking of structures by resolution (from the worst to the best):

Neutron: 1-GOJNIF, 1-ZEYVAA, 1-XAXMEP, 2-SITKUB, 3-QOSZON, 4-NEBNEO, 5-NOBBOX, 6-MIGKIY, 6-UJABOX, 7-TIWXOP

X-ray: 1-QOSZON, 1-TIWXOP, 2-XAXMEP, 3-SITKUB, 4-MIGKIY, 5-ZEYVAA, 6-NEBNEO, 6-UJABOX, 7-NOBBOX, 7-GOJNIF

S1.4. Overall ranking of structures (from the worst to the best):

Neutron: 3-XAXMEP, 4-GOJNIF, 6-NEBNEO, 6-ZEYVAA, 10-SITKUB, 10-UJABOX, 12-TIWXOP, 13-MIGKIY, 14-QOSZON, 18-NOBBOX

X-ray: 8-ZEYVAA, 9-TIWXOP, 10-MIGKIY, 11-QOSZON, 12-XAXMEP, 12-UJABOX, 13-NEBNEO, 17-GOJNIF, 18-SITKUB, 25-NOBBOX

Neutron-X-ray: 14-ZEYVAA, 15-XAXMEP, 19-NEBNEO, 21-GOJNIF, 21-TIWXOP, 23-MIGKIY, 25-QOSZON, 28-UJABOX, 28-SITKUB, 43-NOBBOX

S2. Ranking of structures by refinement quality (neutron, IAM and HAR):

S2.1. Ranking of structures by goodness of fit (from the worst to the best):

Neutron: 1-XAXMEP, 2-TIWXOP, 3-GOJNIF, 4-QOSZON, 5-ZEYVAA, 6-NOBBOX, 6-UJABOX, 7-SITKUB, 8-NEBNEO, 9-MIGKIY

X-ray (IAM): 1- NEBNEO, 2- UJABOX, 3-XAXMEP, 4-SITKUB, 5-MIGKIY, 6-GOJNIF, 7-TIWXOP, 8-ZEYVAA, 9-NOBBOX, 10-QOSZON

X-ray (HAR): 1-SITKUB, 2-XAXMEP, 3-UJABOX, 4-NEBNEO, 5-TIWXOP, 6-MIGKIY, 7-GOJNIF, 8-NOBBOX, 9-QOSZON, 10-ZEYVAA

S2.2. Ranking of structures by R (from the worst to the best):

Neutron: 1-UJABOX, 2-XAXMEP, 3-TIWXOP, 4-SITKUB, 5-ZEYVAA, 5-GOJNIF, 6-MIGKIY, 7-NEBNEO, 8-NOBBOX, 9-QOSZON

X-ray (IAM): 1-ZEYVAA, 2-UJABOX, 3-MIGKIY, 4-NEBNEO, 5-XAXMEP, 6-TIWXOP, 7-QOSZON, 8- NOBBOX, 9-SITKUB, 10-GOJNIF

X-ray (HAR): 1-ZEYVAA, 2- MIGKIY, 3- UJABOX, 4-NEBNEO, 5-XAXMEP, 6-TIWXOP, 7-NOBBOX, 8-SITKUB, 9-QOSZON, 10-GOJNIF

S2.3. Ranking of structures by wR2 (from the worst to the best):

Neutron: 1-SITKUB, 2-UJABOX, 3-GOJNIF, 4-NEBNEO, 5-MIGKIY, 6-ZEYVAA, 7-NOBBOX, 8-XAXMEP, 9-QOSZON, 10-TIWXOP

X-ray (IAM): 1-ZEYVAA, 2- MIGKIY, 3-XAXMEP, 4- UJABOX, 5-QOSZON, 6-TIWXOP, 7-SITKUB, 8-NOBBOX, 9-NEBNEO, 10-GOJNIF

X-ray (HAR): 1-ZEYVAA, 2-MIGKIY, 3-XAXMEP, 4-UJABOX, 5-TIWXOP, 6-NEBNEO, 7-QOSZON, 8-NOBBOX, 9-SITKUB, 10-GOJNIF

S2.4. Ranking of structures by residual density Δρ range (from the worst to the best):

Neutron: 1-ZEYVAA, 1-XAXMEP, 2-MIGKIY, 3-UJABOX, 4-NOBBOX, 5-GOJNIF, 6-SITKUB, 7-TIWXOP, 8-QOSZON, 9-NEBNEO

X-ray (IAM): 1-ZEYVAA, 2-XAXMEP, 3-NEBNEO, 4-TIWXOP, 5-MIGKIY, 6-SITKUB, 7-NOBBOX, 8-UJABOX, 9-GOJNIF, 10-QOSZON

X-ray (HAR): 1-ZEYVAA, 2-XAXMEP, 3-NEBNEO, 4-TIWXOP, 5-MIGKIY, 6-SITKUB, 7-UJABOX, 8-NOBBOX, 9-GOJNIF, 10-QOSZON

S2.5. Overall ranking of structures (from the worst to the best):

Neutron: 12-XAXMEP, 12-UJABOX, 16-GOJNIF, 17-ZEYVAA, 18-SITKUB, 22-TIWXOP, 22-MIGKIY, 25-NOBBOX, 30-QOSZON, 28-NEBNEO

X-ray (IAM): 11-ZEYVAA, 13-XAXMEP, 15-MIGKIY, 16-UJABOX, 17-NEBNEO, 23-TIWXOP, 26-SITKUB, 32-QOSZON, 32-NOBBOX, 35-GOJNIF

X-ray (HAR): 12-XAXMEP, 13-ZEYVAA, 15-MIGKIY, 17-UJABOX, 17-NEBNEO, 20-TIWXOP, 24-SITKUB, 31-NOBBOX, 35-QOSZON, 36-GOJNIF

Neutron-X-ray (IAM): 25-XAXMEP, 28-UJABOX, 28-ZEYVAA, 37-MIGKIY, 44-SITKUB, 45-TIWXOP, 45-NEBNEO, 51-GOJNIF, 57-NOBBOX, 62-QOSZON

Neutron-X-ray (HAR): 24-XAXMEP, 29-UJABOX, 30-ZEYVAA, 37-MIGKIY, 42-TIWXOP, 42-SITKUB, 45-NEBNEO, 52-GOJNIF, 56-NOBBOX, 65-QOSZON

S2.6. Overall ranking of structures by data-refinement quality (neutron, IAM and HAR) (from the worst to the best):

Neutron: 15-XAXMEP, 20-GOJNIF, 22-UJABOX, 23-ZEYVAA, 28-SITKUB, 34-TIWXOP, 34-NEBNEO, 35-MIGKIY, 43-NOBBOX, 44-QOSZON

X-ray (IAM): 19-ZEYVAA, 25-XAXMEP, 25-MIGKIY, 28-UJABOX, 30-NEBNEO, 32-TIWXOP, 43-QOSZON, 44-SITKUB, 52-GOJNIF, 57-NOBBOX

X-ray (HAR): 21-ZEYVAA, 24-XAXMEP, 25-MIGKIY, 29-UJABOX, 29-TIWXOP, 30-NEBNEO, 42-SITKUB, 46-QOSZON, 53-GOJNIF, 56-NOBBOX

Neutron-X-ray (IAM): 40-XAXMEP, 42-ZEYVAA, 56-UJABOX, 60-MIGKIY, 64-NEBNEO, 66-TIWXOP, 72-GOJNIF, 72-SITKUB, 87-QOSZON, 100-NOBBOX

Neutron-X-ray (HAR): 39-XAXMEP, 44-ZEYVAA, 57-UJABOX, 60-MIGKIY, 63-TIWXOP, 64-NEBNEO, 70-SITKUB, 73-GOJNIF, 90-QOSZON, 99-NOBBOX

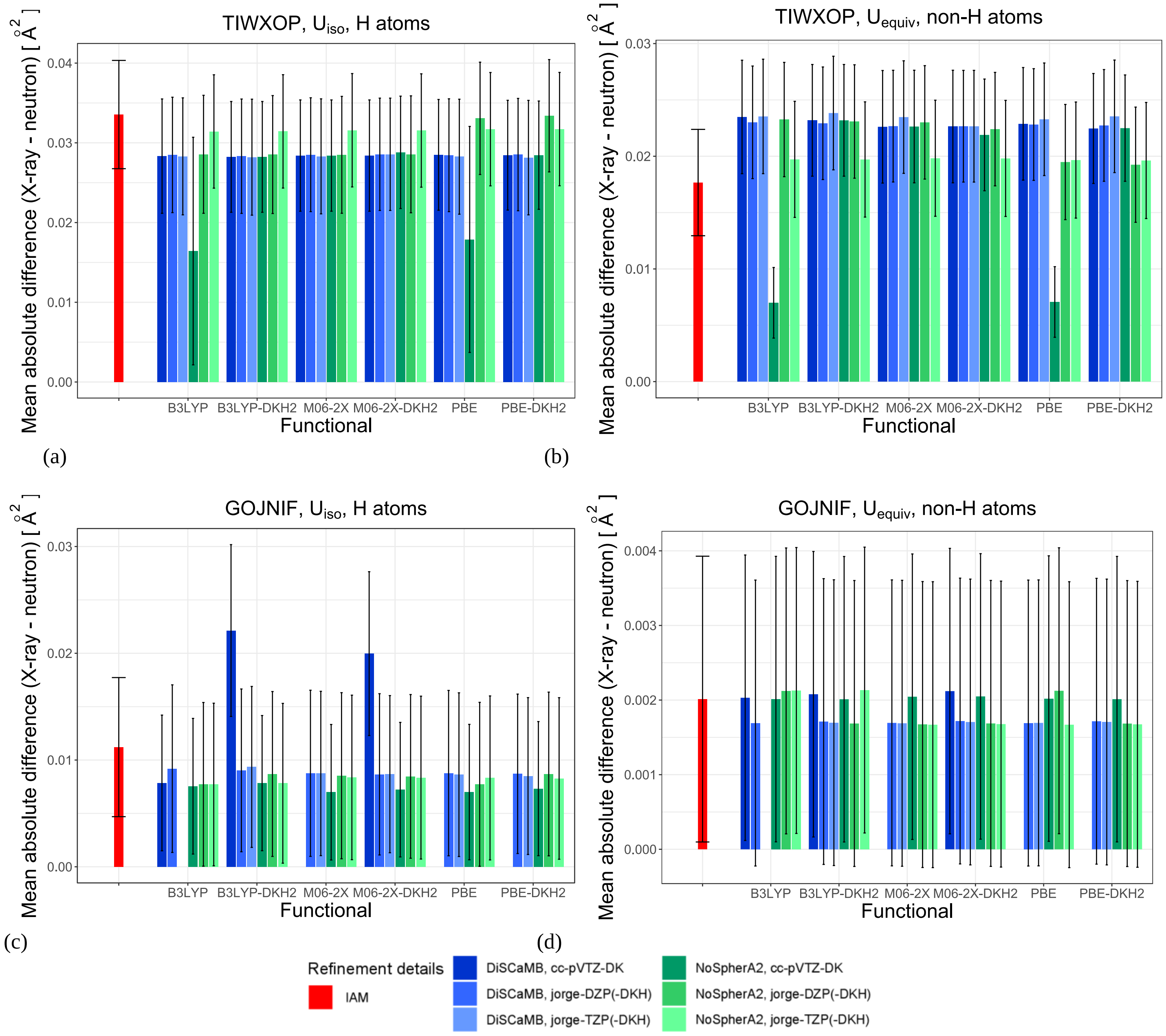

Figure S1. Mean absolute difference (MAD) between the X-ray-derived values of U_{iso} of hydrogen atoms (U_{equiv} of non-hydrogen atoms) and the neutron-derived values of U_{equiv}: (a) TIWXOP, hydrogen atoms, (b) TIWXOP, non-hydrogen atoms, (c) GOJNIF, hydrogen atoms, (d) GOJNIF, non-hydrogen atoms. Error bars depict the mean combined neutron and X-ray standard deviation.
